# Magnetic DNA Origami Nanorotors

**DOI:** 10.1002/adma.74245

**Published:** 2026-07-30

**Authors:** Lennart J. K. Weiß, Florian Rothfischer, Yihao Wang, Christoph Pauer, Xin Yin, Kevin Lang, Rabia Amin, Thomas Tsalos, Susanne Kempter, Jan Lipfert, Tim Liedl, Friedrich C. Simmel, Joe Tavacoli, Aidin Lak

**Affiliations:** ^1^ Department of Bioscience TUM School of Natural Sciences Technical University Munich Garching Germany; ^2^ Institute For Electrical Measurement Science and Fundamental Electrical Engineering and Laboratory for Emerging Nanometrology (LENA) Braunschweig Germany; ^3^ Department of Physics Ludwig Maximilians Universität München Munich Germany; ^4^ Institute of Physics University of Augsburg Augsburg Germany

**Keywords:** collective properties, DNA origami, magnetic actuation, magnetic nanocubes, magnetic nanorotors, molecular devices, monte carlo simulations

## Abstract

Self‐assembled DNA nanostructures show great promise as functional devices, highly configurable materials, and in nanorobotics. Magnetic control provides a powerful and broadly applicable actuation mechanism due to its programmability, compatibility with biological entities, and orthogonality to chemical or electrical stimuli. Here we demonstrate magnetic nanoactuators by leveraging the unique site‐specificity of DNA origami to assemble magnetic nanocubes with high magnetization and magnetic anisotropy on high‐aspect ratio DNA origami bundles. We trace and control 100s of our DNA origami nanorotors at the single‐rotor level and demonstrate their magnetic clamping and controlled rotation under uniform and rotating magnetic fields. By varying the population and inter‐particle spacing of the nanocubes, magnetic torque values on the order of 10‐100 pN nm are calculated at field strengths < 10 mT. Monte Carlo simulations reveal that the assembly of nanocubes on DNA origami rotors leads to collective magnetic properties, with numerically estimated torque values in good agreement with the experiments. Our work demonstrates a proof‐of‐concept of nanoscale magnetic actuators for potential uses as programmable torque nano‐probes and in nanorobotics.

## Graphical abstract

Magnetic actuation is a powerful and broadly applicable actuation mechanism due to its programmability and compatibility with biological entities. Here we demonstrate magnetic DNA origami nanorotors (MADONAs) by assembling magnetic nanocubes on DNA origami. Our MADONAs exert biological torques at low magnetic fields, setting the foundation for working toward new biorthogonal nanoactuators where the actuation is decoupled from the structure.

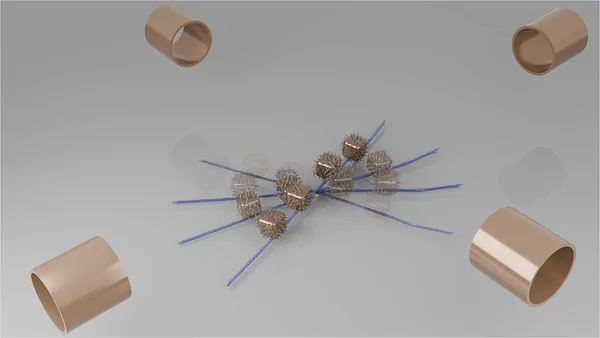

## 1 Introduction

The application of forces and torques through magnetic manipulation at the nano‐ and microscales has enabled a broad range of measurement modalities and critically advanced the understanding of molecular and cellular mechanics as well as cell migration, proliferation, differentiation, and downstream signaling pathways [1, 2, 3, 4, 5, 6, 7, 8, 9, 10, 11, 12, 13, 14]. Magnetic particles provide a handle to control motion on the microscale and below via their coupling to external magnetic fields [15]. Accordingly, they have been employed in numerous micro‐ and nanorobotic systems, particularly those directed toward the study of single‐molecule biological systems and healthcare applications based on targeted drug delivery [12, 16, 17, 18, 19, 20, 21, 22, 23, 24]. Early studies mainly used micron‐sized magnetic beads in magnetic tweezers assays and have provided key insights into the mechanics of nucleic acids, cells, and recently proteins [13, 25, 26, 27, 28, 29, 30]. However, micron‐sized particles provide only limited control at the molecular scale and often bind to several molecules simultaneously due to their size and multivalency [6]. To address the bead's multivalency issue, magnetic nanoparticles with monovalent binding ability in combination with magnetic micro‐tweezers have been developed [31, 32]. However, magnetic nanoparticles with sizes < 25 nm have, inherently, a small magnetic moment *m* of ≈ 1‐2 × 10^−18^ A m^2^, and are only able to generate ≈ fN forces even at hundreds of mT [33, 34]. This force regime is orders of magnitudes too low to manipulate cellular processes, such as molecular motors and cell surface receptors that typically require 1‐100 pN [19].

Nature solves this challenge by exploiting self‐assembly. Magnetotactic bacteria biomineralize a chain of iron oxide nanoparticles within their magnetosome organelles, thereby increasing their net magnetic moment by 20‐fold to navigate their microenvironment following the Earth's magnetic field [35, 36, 37]. Such architectures can be mimicked by patterning nanoparticles on an external template and have been achieved by utilizing lithographical molding techniques, colloidal chemistry, and soft synthetic templates [16, 17, 38, 39, 40, 41, 42, 43, 44, 45, 46, 47]. However, control over the number, patterning, and orientation of magnetic nanoparticles using soft and polymeric templates remains a challenge, limiting in turn the ability to precisely define the properties and functionalities of the resulting assemblies.

DNA origami, where single‐stranded DNA (ssDNA) scaffolds are folded into precisely defined 3D‐shapes via hybridization of a complementary set of ssDNA oligomers, holds promise for overcoming these limitations due to its site‐specific addressability [48, 49, 50, 51, 52]. DNA origami can capture and organize proteins, inorganic nanoparticles, and fluorescent dyes, when tagged with complementary base sequences, with a high spatial resolution < 1 nm [53, 54, 55, 56, 57, 58, 59, 60]. Moreover, DNA origami scaffolds such as six‐helix‐bundles (6HB) with persistence lengths on the scale of 1.6‐1.9 µm [29, 61] are sufficiently mechanically rigid to enable efficient force and torque transfer for actuation in defined degrees of freedom [62, 63, 64, 65, 66, 67]. Recently, dynamic DNA origami nanomachines have been developed that utilize chemical and physical triggers such as strand hybridization and displacement, thermal melting, and electric fields [62, 67, 68, 69, 70, 71, 72, 73]. Salt concentration, pH, and temperature have been employed to switch DNA origami nanomachines between pre‐programmed states [74, 75, 76, 77], but as these variables are tightly regulated in biological settings, this class of DNA origami nanomachines does not function under biological conditions. Electric fields have been used as an efficient physical control mechanism for DNA origami arms and rotors by coupling to their inherent charges and have shown adjustable, rapid, and repeatable unidirectional motion [67, 78]. In this manner, torque values on the order of tens of pN nm, comparable to biological rotary motors, have been achieved [65, 79, 80, 81]. However, in electrically driven DNA origami, the applied electric fields act directly on the charges of the origami structure, thus offering limited degrees of motional freedom and actuation. Moreover, the electrophoretic actuation of a 400 nm origami lever requires a 200 V bias voltage, a voltage that can cause local heating and damage both DNA nanostructures and any biologically probed material [82].

In contrast to electric fields, magnetic fields offer critical advantages for biological and biomedical applications, as they are innocuous for biological tissue and only minimally attenuated. Consequently, magnetic micro‐ and nano‐devices are attractive for remote manipulation of biological processes and in vivo targeted drug delivery. Additionally, integrating magnetic functionality into DNA origami nanorotors decouples function from structure, thus opening a biorthogonal means of actuating without introducing heat and damaging living beings. Previously, DNA origami has been used to enable single‐molecule actuation with magnetic beads [83] and as a fiducial marker [12]. Hybrid magnetic micro‐actuators (∼3‐10 µm) were constructed by attaching a magnetic bead to a DNA origami micro‐lever [83] and rod‐like DNA origami were used as fiducial markers to track the rotation of magnetic DNA clutches [12].

In this study, we present magnetic DNA origami nanorotors (MADONAs) that we engineer by decorating six‐helix‐bundles (6HB) DNA origami nanostructures with highly anisotropic cobalt‐and zinc‐doped ferrite magnetic nanocubes (MNCs). Our ≈ 17 nm engineered MNCs can be efficiently patterned on DNA origami and are much more magnetic than iron oxide NPs [84, 85]. Here, we demonstrate a programmable torque response by changing field strengths, particle populations, and spacing. Through rational design, torque values of 10‐100s of pN nm at single‐digit mT field strengths are achieved, thus opening new opportunities to interface with biological processes in the relevant torque regime on the nanoscale. Our genuinely nanoscale MADONAs will accelerate the application of nano‐mechanical devices to real‐world scenarios.

## 2 Results and Discussion

### 2.1 MADONAs With Modular Design

**FIGURE 1:**
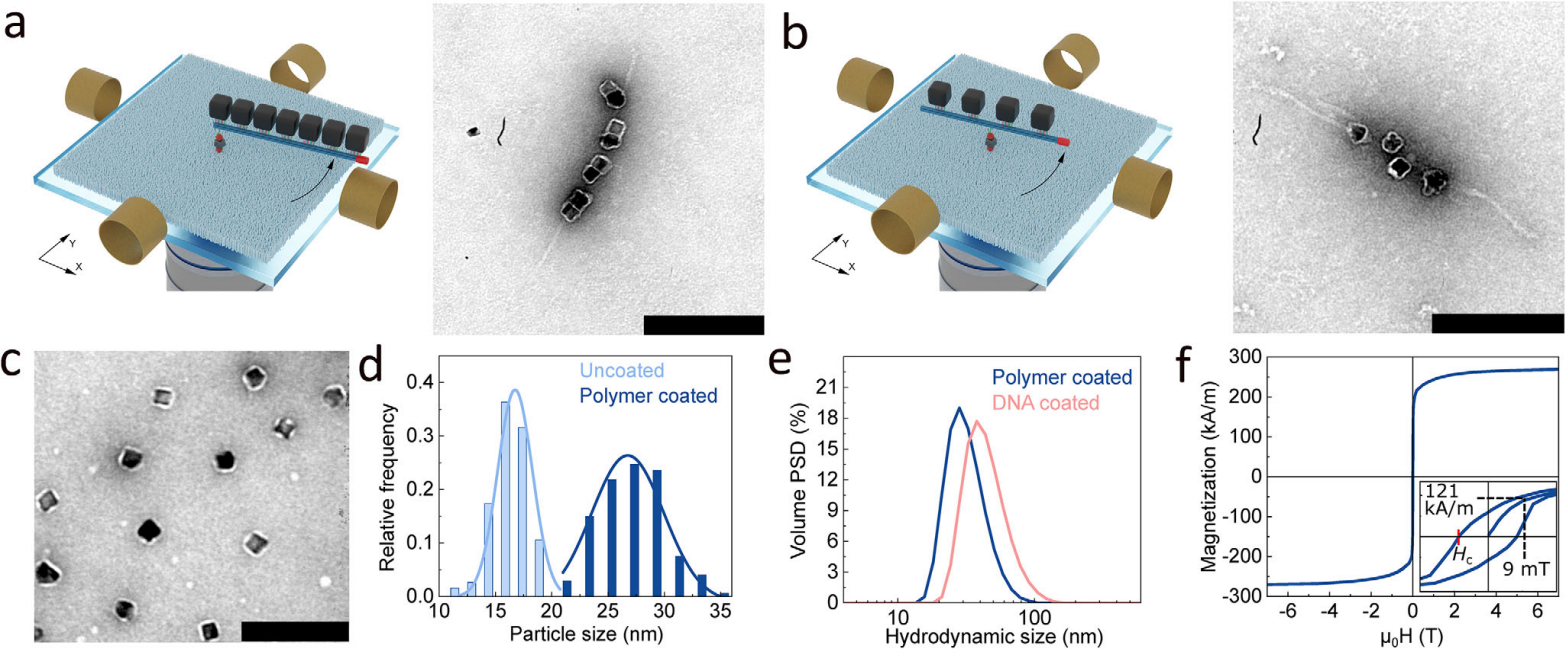
Designs of magnetic DNA origami nanorotors (MADONAs) and structural and magnetic characteristics of MNCs. (a,b) Schematic of the magnetic actuation assays at the single‐rotor level (left) and negative‐stain transmission electron microscopy (TEM) micrograph (right) of a 6HB rotor (a) patterned with multiple MNCs (multi‐MNC design) with an end‐pivot modification and (b) patterned with 4 MNCs (4‐MNC design) and a centered pivot point for reduced motion in z‐direction. The pivot point consists of a single biotin‐modification. The fluorescent tips of the 6HB are shown in red. (c) TEM image of polymer‐coated MNCs. The scale bar is 100 nm. (d) Particle sizes of MNCs prior (uncoated) and after polymer coating (polymer coated) obtained from TEM analyses. (e) Volume‐weighted particle hydrodynamic diameters (DLS) of MNCs after polymer coating and after DNA labeling. (f) Magnetic hysteresis loops measured on MNC in suspension at 298 K. Magnetization values were converted to A m^−1^ using a density ρ = 5.28 g cm^−3^, specific to the chemical composition of our particles. The particles exhibit a coercive field *H*_c_ = 8 mT in suspension; at 9 mT, *M* = 121 kA m^−1^, as determined from the virgin magnetization curve.

We build magnetic nanorotors using a ≈ 415 nm‐long 6HB DNA origami as scaffold. The 6HB are designed with a fluorescently‐labeled tip with 42 Atto655 dyes to track their rotational motion using total internal reflection fluorescence microscopy (TIRFM) (Figure 1 and Supporting Information). Further, the MADONAs feature a single ssDNA‐biotin extension that is tethered to the measurement glass surface via a NeutrAvidin‐biotin interaction (Figure 1a,b) that serves as a pivot point for free hemispherical/in‐plane rotation. Finally, the designs have multiple binding sites for MNCs, each one consists of six 8 nucleotide (nt)‐long poly‐dA extensions that hybridize with 20 nt‐long poly‐dT oligos on the MNCs.

We created two different MADONA designs that vary in number, placement, and separation of their MNC‐binding sites to study how magnetic couplings between neighboring MNCs affect their magnetic response. In the first design (referred to as multi‐MNC MADONA), binding sites are placed ≈ 6‐8 nm from each other over a total length of ≈ 216 nm (see details in Figure S1–S3). The multi‐MNC MADONA can accommodate on average 7‐8 MNCs with a particle center‐to‐center distance (R_c‐c_) of ≈ 17 nm (Figure 1a). The continuous binding of eight MNCs along the whole structure can be seen in negative‐stain TEM micrograph (Figure 1a, right panel). In the multi‐MNC MADONA design, the pivot point (i.e., the center of rotation) is placed on one end of the rotor (Figure 1a). The second design (termed 4‐MNC MADONA) features four defined binding sites corresponding to R_c‐c_ of ≈ 64 nm and a centered pivot point. Quantitative binding of four MNCs to the second design can be seen in negative‐stain TEM micrograph (Figure 1b, right panel). In the 4‐MNC MADONA design, we placed the pivot point at the center of the structure to restrict its movement along the z‐axis for quantitative torque calculations (Figure 1b).

### 2.2 Synthesis of Colloidal MNCs With Functional DNA Brushes

We use custom colloidal MNCs with a dense ssDNA shell and a high magnetic moment and anisotropy to build magnetic responsive 6HB rotors. We synthesized the MNCs following our published procedures (see the Supporting Information for protocols) [84, 85, 86]. The MNCs have the chemical composition Co_0.4_Zn_0.2_Fe_2.4_O_4_ and a mean edge length *L* of 16.7 ± 3.7 nm (N = 190), as determined from the analysis of TEM images (Figure 1d). After coating the MNCs with a thin poly‐(maleic anhydride‐alt‐1‐octadecene)‐PEG(3)‐azide block‐co‐polymer shell [84], the particle size *L* increases to 26.7 ± 3.2 nm (N = 176), as deduced from size analysis of negatively stained TEM images (Figure 1c,d). Finally, grafting 20 nt oligo(deoxythymidine) ssDNA strands on the MNCs (≈ 0.12 strands/nm^2^) increases the particle hydrodynamic size from 32.6 ± 0.6 nm to 45.8 ± 1.0 nm (Figure 1e, see the Supporting Information for protocols). Magnetic hysteresis loops measured on MNCs in buffer show a saturation magnetization of *M*_s_ = 269 kA m^−1^, corresponding to an average particle magnetic moment of *m* = 1.25 × 10^−18^ A m^2^ (Figure 1f), which is 8.5‐fold larger than the magnetic moment of commercial 15 nm spherical iron oxide NPs [87]. Our MNCs have an effective anisotropy constant of *K*_eff_ = 50200 J m^−3^ (details in Figure S4), which is an order of magnitude larger than typical iron oxide NPs^88^ and also significantly larger than 1 µm MyOne beads [89, 90].

### 2.3 Multi‐MNC MADONAs Can be Clamped at Single‐Digit mT Magnetic Fields and Rotated up to Few Hz Synchronously

**FIGURE 2:**
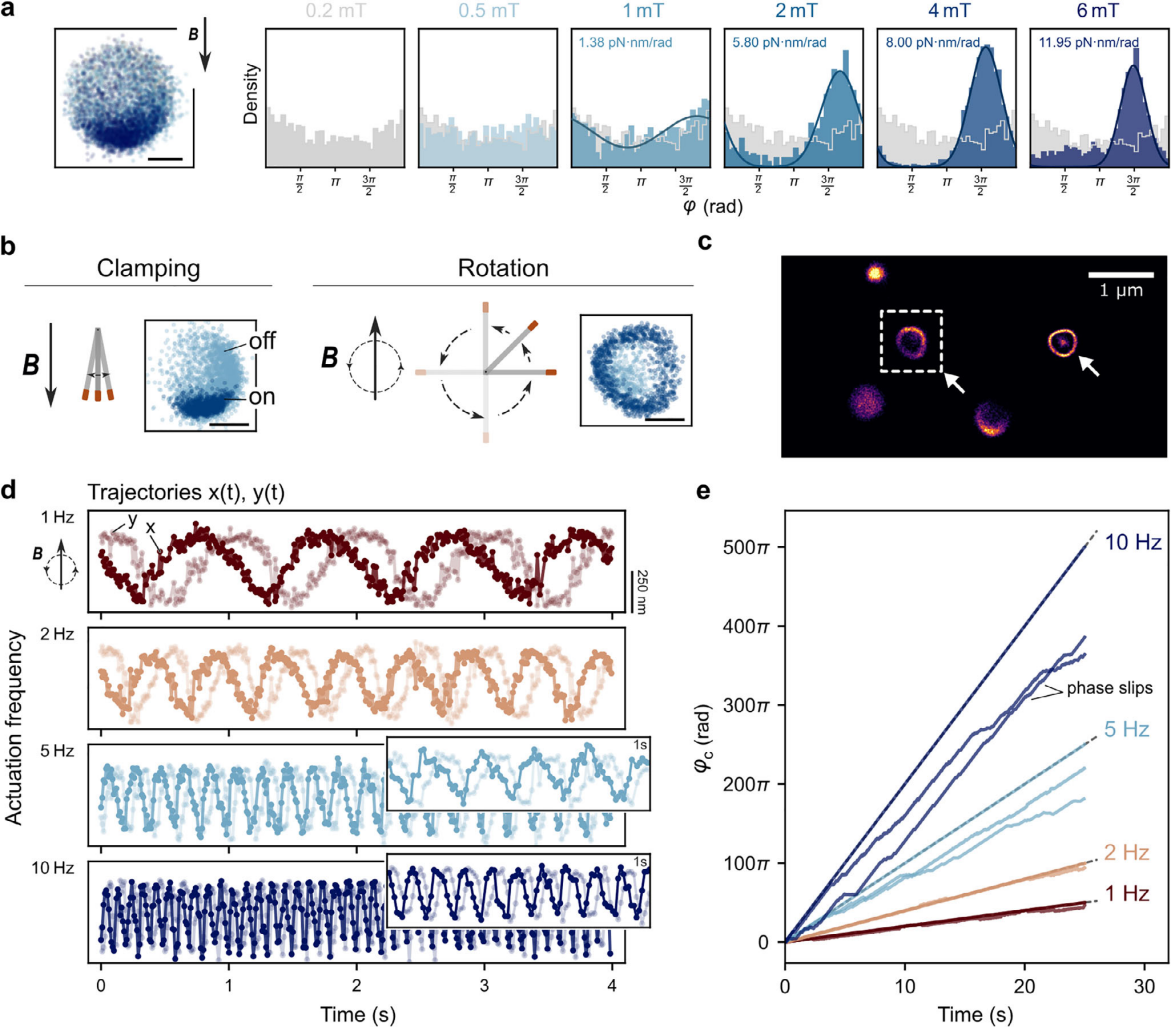
Multi‐MNC MADONAs can be controlled by static and rotating magnetic fields. (a) Tracked positions of a multi‐MNC MADONA rotor and histograms of the tracked rotation angle during magnetic clamping experiments at different magnetic field strengths (see also Figures S5–S7). (b) Clamping and rotation actuation modes and their effects on rotor motion. Light blue shows the localizations during diffusion with the field off and dark blue upon magnetic actuation. Scale bars are 250 nm. (c) Localization heatmap of a TIRFM‐RMF experiment in false colors. Arrows indicate functional rotors. The dashed box corresponds to the best‐performing rotor that follows the RMF synchronously with its rotation trajectories shown in panels (d, e). (d) Particle trajectories in Cartesian coordinates of an exemplary rotor during actuation at 2.7 mT and 1, 2, 5, and 10 Hz rotating field frequencies. The insets show 1 s zoom‐ins. (e) Particle trajectories are shown as cumulative angle for three different rotors when driven by RFMs at 2.7 mT with the rotation frequencies indicated in the figure legend.

We demonstrate magnetic actuation and control of our MADONAs by attaching their biotin‐NeutrAvidin pivot points to a biotin‐PEG silanized flow cell on a total internal reflection fluorescence microscope (TIRFM) that is equipped with two pairs of pseudo‐Helmholtz coils (Figure 1a,b) [67]. The Helmholtz coils enable full field control in the (*x,y*)‐plane, including the application of static or rotating magnetic fields (RMFs) of the form *B(t)* = *B*_x_(cos*(2π ft))—B*_y_(sin*(2π ft))*, with *B*_x_ = *B*_y_ the field strength, *f* the field frequency, and *t* time. Upon binding of the MADONAs to the surface, we incubated them with an excess of MNCs to saturate all binding sites (Section S3). After washing out unbound MNCs, uniform magnetic fields are applied and the rotors' angular position is recorded in high‐viscosity buffer (*η* ≈ 12.5 mPa s, Figure 2a,b) to track the MADONAs reliably at 5 ms frame time. At field amplitudes above 2 mT, the multi‐MNC MADONAs are magnetically restricted in their diffusive motion at the single‐rotor level (Figure 2a and Figure S5). As the field strength is increased, the angular fluctuations are further restricted, and we quantify the magnetic trap stiffnesses from the magnitude of angular fluctuations (Figure S6). When the external field is switched off, the MADONAs return to free Brownian motion (Figure S7).

After clamping the multi‐MNC MADONAs along a single axis successfully, we investigated their rotational dynamics under in‐plane RMFs (Figure 2b). Looking at the localization heatmap (Figure 2c) recorded under the RMFs, we observed a population of MADONAs that follow the RMF and make a full rotation circle (marked with arrows). We traced the magnetic rotations of 100s of MADONAs within a single field of view for different actuation frequencies (*x, y*‐locations of a single rotor in Figure 2d, cumulative angles for three rotors in Figure 2e). While some of the multi‐MNC MADONAs synchronously follow the RMF up to 10 Hz (Figure 2e, ideal trajectories shown as dashed lines), others experience phase slips and only incompletely follow the external field, in particular at higher rotation speeds. These so‐called drop‐out points, where the MADONAs cannot follow the external field anymore, appear when the exerted magnetic torque, *τ* = *m*_eff_ *B*, is less than the viscous drag and the restoring torques from surface‐origami interactions that the rotors experience (Section S7). We expect that MADONAs with low effective magnetic moments cannot sustain co‐rotation with the RMF, in particular at low field strengths and fast rotation speeds.

In general, we observe three rotor behaviors: (1) rotors that follow the field synchronously under the conditions tested, (2) rotors that are actuated by the field but also experience phase slips resulting in a lower average velocity, and (3) rotors that remain freely diffusing. The freely diffusing rotors are presumably non‐laden with MNCs, while driven and synchronous rotors likely span from being partially to fully loaded with MNCs. Indeed, TEM images of multi‐MNC MADONAs support the non‐quantitative binding of MNCs due to tightly placed binding sites (Figure S8). Furthermore, rotors laden with the same number of MNCs can have different net magnetizations depending on the initial binding orientation of the MNCs that may frustrate the system from reaching its lowest energy state (i.e., where the particle magnetic moments are aligned head to tail along the external field). Nevertheless, our measurements clearly demonstrate that we can magnetically actuate the MADONAs.

### 2.4 Controlled Actuation by Patterning Four MNCs (4‐MNC MADONAs) at Specific Inter‐Particle Distances

Our data for multi‐MNC MADONAs suggest that placing the particle binding sites close to each other leads to large variations in MNC binding occupancy and thus rotor properties, likely due to steric hinderance between adjacent MNCs that leads to fewer MNCs per rotor than would theoretically possible (Figures S1 and S8). Accordingly, we next focused on 4‐MNC MADONA design, with four attachment sites spaced ≈ 64 nm apart, which is significantly larger than the size of the DNA‐functionalized MNCs. For this design, we observe ≈ 54% fully assembled MADONAs with 4 MNCs, ≈ 21% MADONAs with 3 or less MNCs and 25% rotors with 1‐2 dimer or 5 MNCs (see Figure S9 for AFM images and Figure S10 for a statistical distribution analysis based on TEM micrographs).

**FIGURE 3:**
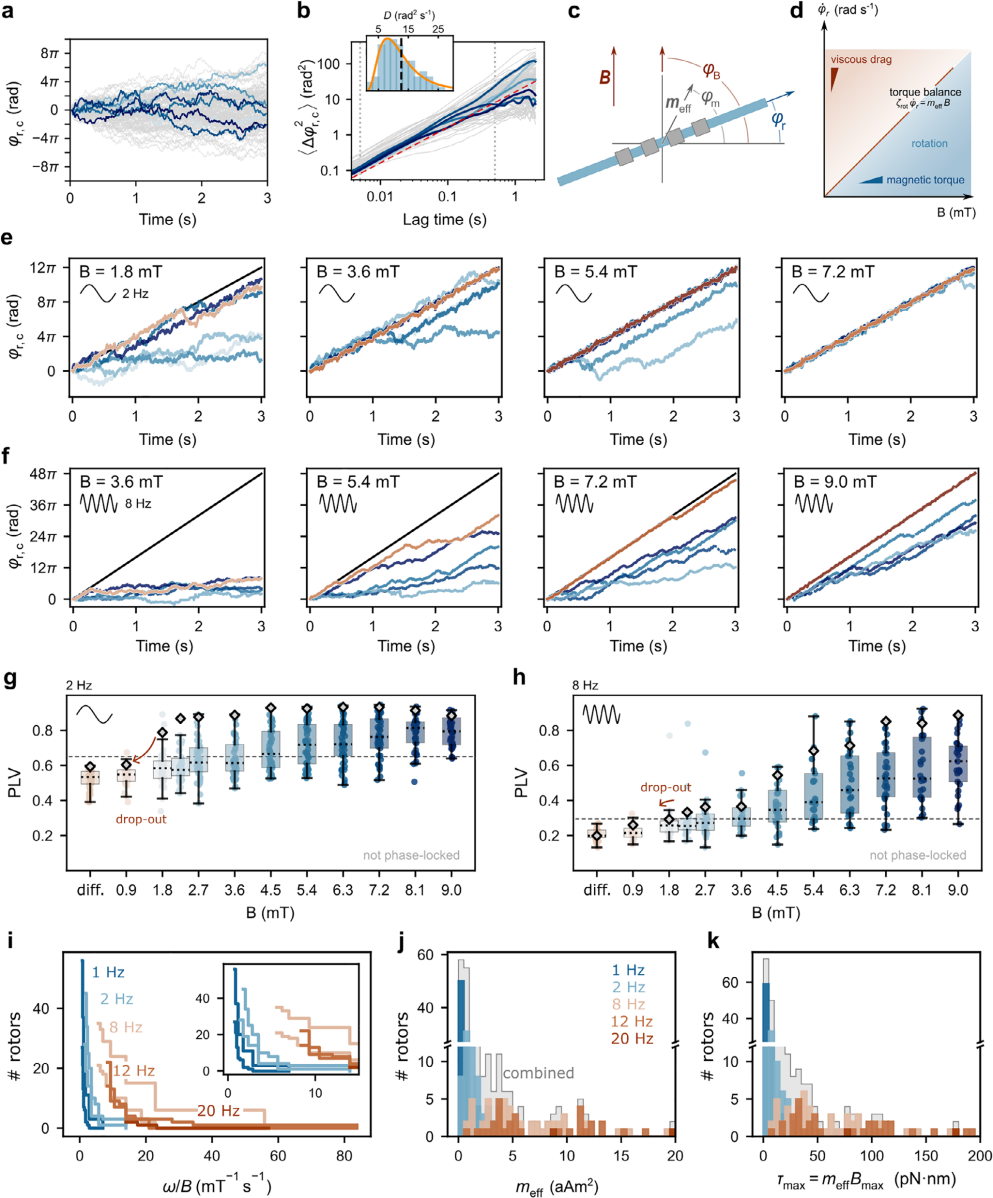
4‐MNC MADONAs driven by rotating magnetic fields. (a) Diffusive rotor trajectories with the magnetic field off. (b) Mean‐squared angular displacement 〈Δφ^2^〉 of the rotor ensemble during diffusion (highlighted curves correspond to trajectories in (a)). The dashed red line is the mean power spectrum. Inset: Histogram of extracted rotational diffusion coefficients (*n* = 128) and fit of a log‐normal distribution (R^2^ = 0.94), yielding an experimental mean *D*_rot_ = 12.7 rad^2^ s^−1^ and fitted most likely value *D*_rot_ = 8 rad^2^ s^−1^. (c) Coordinate system for analysis of rotor motion; *φ*_r_ is the rotor orientation, *φ*_m_ is the axis of the effective magnetic moment *m*_eff_, and *φ*_B_ is the orientation of the external magnetic field. (d) Co‐rotation requires an effective magnetic moment sufficient to overcome viscous drag under external actuation. (e,f) Exemplary trajectories of nanorotors during actuation with 2 Hz and 8 Hz and different field strengths, respectively. (g,h) Phase‐locking values (PLVs) for rotors actuated by RMFs of increasing strength at 2 Hz (g, *n* = 49 rotors) and 8 Hz (h, *n* = 29 rotors). Box plots show the ensemble distribution, with individual rotors shown as points. Diamond markers denote the two representative rotors highlighted in orange in (e) and (f). The dashed line indicates the phase‐locking threshold, defined as > PLV_diff_, where PLV_diff_  is the PLV of freely diffusing rotors evaluated against a virtual RMF. Rotors with PLVs below this threshold were classified as not field‐driven (see Figure S22). The orange arrow marks the drop‐out field strength of the highlighted rotor. (i) Fraction of driven 4‐MNC MADONAs as a function of normalized inverse magnetic field strength for different driving frequencies based on overlays from 9 independent drop‐out assays (see Figures S16–S20). (j) Lower‐bound *m*_eff_ as obtained from the drop‐out field strengths shown in panel (i) using torque balance. (k) Histograms of maximum torques exerted by 4‐MNC MADONAs, as calculated from the *m*_eff_ in panel (j) at the maximum field strength of 9 mT.

Similar to the multi‐MNC MADONAs, the 4‐MNC MADONA undergo diffusive motion if the external field is off (Figure 3a), allowing us to assess the rotational diffusion coefficient and drag coefficient *ζ* of the rotors from the mean squared angular displacements (Figure 3b). The diffusion coefficients were log‐normally distributed (*µ* = 2.39, *σ* = 0.55, R^2^ = 0.94) with a most probable value of *D*_rot_ = 8 rad^2^ s^−1^, which corresponds to a most likely drag coefficient of *ζ*_rot_ = 2.7 × 10^−22^ N m s. This value is in good agreement with the bulk rotational drag coefficient of 2.4 × 10^−22^ N m s for a rod with a length of 415 nm and width of 8 nm in a medium with viscosity *η* ≈ 12.5 mPa s [65], the approximate dimensions of our construct and conditions of our experiments.

To understand the rotational behaviors of the rotors when they are driven by the external field, we developed a rotation model (Figure 3c, details in the Supporting Information). Here, we considered a 6HB rotor that carries four MNCs with magnetic moments *m_i_*, resulting in an effective magnetic moment *m*_eff_ per rotor. Upon external actuation, this effective magnetic moment experiences a torque *τ*_m_ = *m*_eff_ *B* sin(*φ*_B_—*φ*_m_), which is dependent on its axis' orientation *φ*_m_ relative to the external field *φ*_B_. Since the MNCs are, in first approximation, rigidly coupled to the DNA origami, we assume a constant angle offset between *m*_eff_ and the rotor axis, *φ*_m_ = *φ*_r_ + *Δφ* (Figure 3c), which allows the rotors to co‐rotate with the external field. The rotor's motion under a RMF with constant angular velocity, *φ*_B_ (t) = *ω*_B_ *t*, can be described by a torque balance in the overdamped regime, where the induced magnetic torque must compensate for th viscous drag, the local restoring torque stemming from rotor‐surface interactions, and thermal fluctuations:

(1) $$\zeta \dot{\varphi_{r}}+U^{\prime }\left(\varphi_{r}\right)+\xi \left(t\right)=m_{r}B\sin\left(\omega t-\varphi_{r}+\Delta \varphi \right)$$

Here, *ζ* is the drag coefficient, *U'*(φ_r_) is the spatial derivative of the energy landscape, and *ξ* is Gaussian white noise with 〈ξ(*t*)ξ(*t*′)〉 = 2ζ*k_B_T*δ(*t* − *t*′). By neglecting thermal fluctuations, the rotors are synchronous to the external drive when the angular offset between the external actuation and the rotor, θ =  ω_*B*_ *t* −  φ_*r*_,  stays constant (Figures S12 and S13 for simulated rotor trajectories in the absence and presence of thermal fluctuations). Synchronous motion is possible as long as the magnetic torque compensates (at least) the viscous drag, *m*_eff_ *B* ≥  ζω_*B*_ (Figure 3d). For smaller torques, the phase offset θ no longer locks but drifts and the rotor lags behind the magnetic field. However, if we take local rotor‐surface interactions and noise into account (Section S7 and Figure S14), the critical limit above provides only a lower bound for the magnetic torque needed to sustain co‐rotation.

We now probe the *m*_eff_ (Figure 3d) of the 4‐MNC MADONAs by actuating rotor ensembles at fixed angular speed and with increasing field strength (Figure 3e for 2 Hz and Figure 3f for 8 Hz). Our TIRFM‐RMF assays provide several insights into the rotors dynamics: the fraction of rotors that rotate synchronously with the RMF increases with field strength and decreases with frequency (Figure 3e,f). This concurs with the torque balance described in Equation (1) and its magnetic moment and rotational frequency dependence (Figure 3d). The orange‐colored trajectories (Figure 3e,f) belong to two rotors that can follow the RMF synchronously at high field strengths but show phase‐slips at low field amplitudes (e.g., 5.4 mT at 8 Hz). The blue trajectories correspond to rotors that are clearly driven by the RMF at high field strengths but rotate at a lower angular velocity than the external drive as phase slipping occurs frequently. Rotors that exhibit imperfect synchronization with the external field likely have a low *m*_eff_ and/or a rugged surface‐associated energy landscape that introduces additional restoring torques and impedes phase‐locking. We note that insufficient magnetic torques generate periodic phase slips and eventually lead to complete loss of synchronization under increased viscous drag (Figure S12), but thermal fluctuations (Figure S13) and rugged intrinsic energy landscapes (Figures S14, S15) can produce similar effects. We, therefore, classified all rotors exhibiting rotary trajectories beyond free diffusion as field‐driven, although they are not necessarily all the time perfectly synchronized with the RMF.

To quantify the rotation trajectories of MADONAs, we used the phase‐locking value (PLV; with PLV = 1 perfect synchronization, PLV = 0 unsynchronized trajectories; see also Supporting Information) [91], defined as the temporal average of the angular offset between the rotor orientation and the external drive in the complex plane. In practice, finite acquisition times and thermal fluctuations can yield PLVs above zero even for freely diffusing rotors (PLV_diff_) when evaluated against a virtual RMF, particularly for low frequencies. Therefore, PLV_diff_ provides a relative, frequency‐dependent threshold rather than an absolute synchronization criterion (see Figures S21–S23).

The PLV criterion allowed us to determine, for each rotor and driving frequency, the field strength below which driven rotation could no longer be sustained. The frequency‐resolved drop‐out assays (Figure 3g–i; smoothed raw trajectories in Figures S16–S20) are then used to estimate *m*_eff_ (Figure 3j). The resulting estimates of *m*_eff_ reveal a broad distribution (Figure 3j), corresponding to field‐induced torques from a few to ≈ 100 pN nm at 9 mT (Figure 3k). This broad range likely reflects both rotor‐to‐rotor heterogeneity (in terms of magnetic moments and restoring torques) and the intrinsic ambiguity of defining drop‐out criteria in the rotation assay. Accordingly, we switch to clamping experiments where the said ambiguity of the drop‐out criteria is removed and hence a more accurate value of *m*_eff_ can be obtained.

### 2.5 4‐MNC MADONAs Exert 10s of pN Nm Magnetic Torques at Single‐Digit mT Magnetic Fields: Clamping Experiments and Monte Carlo Simulations

**FIGURE 4:**
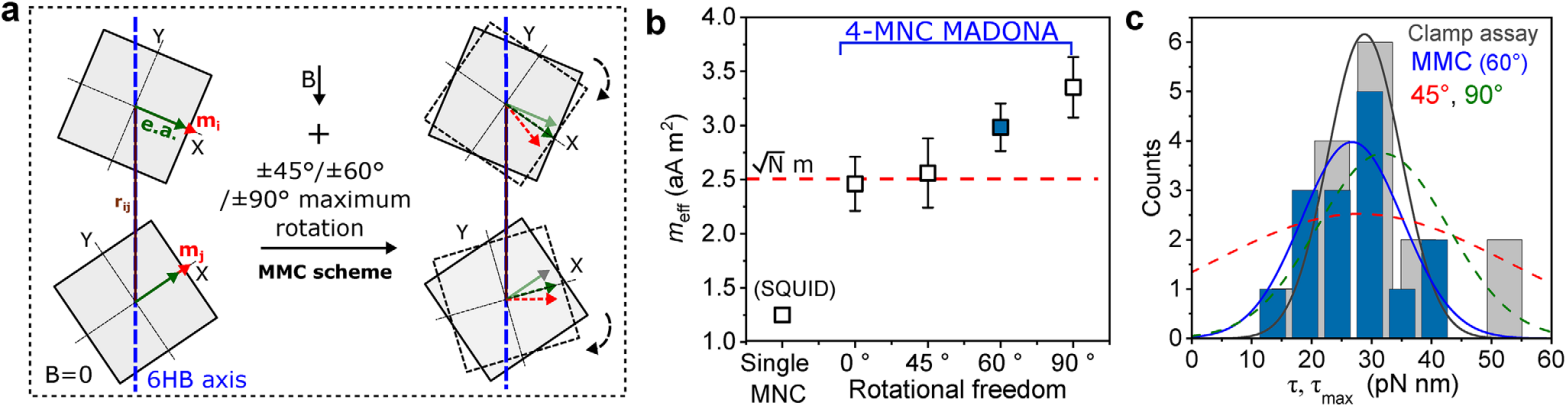
Magnetic torques of 4‐MNC MADONAs from magnetic clamping and Monte Carlo simulations. (a) Physical model of magnetic interactions between two neighboring MNCs and with the external field used in Metropolis Monte Carlo (MMC) simulations. **m_i_** and **e.a**. are the *i* ^th^ particle's magnetic moment and anisotropy axis unit vectors. (b) Effective magnetic moment *m*_eff_ of 4‐MNC MADONA at 9 mT field amplitudes at 0°, 45°, 60°, and 90° rotational freedom obtained from 15 independent MMC simulations. The symbols and error bars are the mean ± S.E.M from the independent simulations. The mean moment *m*_mean_ of a single MNC measured in suspension using a superconducting quantum interference device (SQUID) magnetometer is shown for comparison. The red dashed line shows *m*_eff_ of 4 non‐interacting MNCs in a random cluster. Particle parameters used in the MMC simulations are given in the Supporting Information. (c) Maximum magnetic torques obtained from the clamping assays and MMC simulations at 9 mT with 60° rotational freedom. The solid lines are the best Gaussian fits to the histograms. The red and green dashed lines are the Gaussian fits to the histograms of 45° and 90° rotations, with the Bhattacharyya coefficient of 0.67 and 0.90, indicating a partial and strong overlap with the experimental torque histogram, respectively. Same color coding is applied in (b) and (c).

Next, we developed a 2D dynamic particle‐coupling model for our ferrimagnetic MNCs (Figure 4a) to better understand how the magnetization *m* of individual MNCs interacts to produce *m*_eff_ and to provide a microscopic understanding of the clamping experiments on 4‐MNC MADONAs (Figures S24–S30). We used the Metropolis Monte Carlo (MMC) algorithm to minimize the total free magnetic energy by considering the Zeeman and magnetic anisotropy energies, and dipole‐dipole interactions (for details see Figure S30). For these simulations, we focused on 4‐MNC MADONAs, as this design offers at most binding of 4 MNCs to the 6HB (see Figure S10 for TEM micrographs). We assumed that the bound MNCs can rotate physically up to a maximum of 90° to align with the field (Figure 4a). From the MMC simulation results (average of 15 MMC runs), we computed the rotor's *m*_eff_ at 9 mT field strength by the vector sum of magnetic moments of four MNCs (Figure 4b). The MMC results show that the simulated *m*_eff_ of 4‐MNC MADONAs is larger than the *m* of a single MNC in suspension (which was experimentally determined via SQUID magnetometry, Figures 1f and 4b). To check if there is collective couplings between MNCs in nanorotors, we compared the simulation data with the analytical expression of *m*_eff_ for a random cluster of *N* non‐interacting MNCs, given by [92].

(2) $$m_{\mathrm{eff}}=\sqrt{N}m\;\left[1+\frac{\left(N-1\right)}{2}{\left(\frac{mB}{3k_{B}T}\right)}^{2}\right]$$

The Equation (2) becomes $m_{\mathrm{eff}}=\sqrt{N}m$, as $\frac{\left(N-1\right)}{2}{\left(\frac{mB}{3k_{B}T}\right)}^{2}$ < 1 at *N* = 4, *B* = 9 mT, and *m*_mean_ = 0.56 aA m^2^ (9 mT). We can observe that the simulated *m*_eff_ values are above $\sqrt{N}m$ limit (dashed red line in Figure 4b) when a nominal particle rotation ≥ 60° is considered in the simulations. At 60° rotation freedom, the *m*_eff_ = 2.98 ± 0.2 aA m^2^ corresponds to a ≈ 60% of the perfect alignment of four MNCs (i.e., *m*_eff_ = 5 aA m^2^, Figure 4b). Comparing this level of alignment with ≈ 45% alignment of single MNCs in suspension at 9 mT (measured by SQUID magnetometry, Figure 1f), there is a 15% alignment gain as a result of assembling of MNCs on the 6HB bundles. Altogether, our experimental and numerical results clearly demonstrate collective magnetic interactions between MNCs on 4‐MNC MADONAs.

We then computed the maximum torques *τ*_max_ by taking the simulated *m*_eff_ and compared it with *τ* values obtained from the magnetic clamping experiments, as both simulations and clamping assays represent the static magnetic spin configuration of the MADONAs (exemplary clamping data in Figures S24–S29, ensemble data in Figure S30). The best agreement between experiments (*τ* = 28.8 ± 6.1 pN nm) and simulations (*τ* = 26.7 ± 8.4 pN nm) was obtained at the 60° rotational freedom by comparing the similarity between two histograms using the Bhattacharyya coefficient (BC) [93]. The BC = 0.96 indicates a very strong overlap between experimental and simulated torque histograms (Figure 4c). Both experiments and simulations suggest that MNCs bound to the bundles are able to rotate to a certain angle in the direction of external fields, resulting in an overall stable magnetization axis of rotors.

Next, we simulated magnetic torques of all non‐stoichiometric rotor configurations we observed in our TEM investigations and statistical analysis (Figure S10). We obtained torque values from ≈ 10 to 80 pN nm for MADONAs with only one MNC and for the rotors fully occupied but having two dimers instead of monomeric MNCs, respectively (Figure S33). Dimers would contribute disproportionately to the total *m*_eff_ of the rotor, as the magnetic moment of their constituting MNCs are well aligned during polymer coating process. Conversely, the subset of rotors with torques below 10 pN nm likely corresponds to rotors carrying fewer than four MNCs, rotors in which the magnetic moments of attached MNCs are partially opposed, or rotors in which one or more MNC moments have an out‐of‐plane magnetization, thus contributing less to the in‐plane torque generation. Our simulated torque values match the experimental torques from the rotation assays (Figure 3k), spanning from a few to ≈100 pN nm, indicating the contribution of different populations to the overall torques, yet with a statistically dominant contribution from perfectly formed MADONAs with a 54% population. Both experimental and numerical values are on the same order of 80 pN nm rad^−1^ torque values reported by Lauback et al. [83] for a magnetic microbead attached to a DNA origami lever, yet these micro‐actuators only operate on the microscale. In contrast, our MADONAs function on the nanoscale by matching the dimension of magnetic torque probes with 6HB nanorotors via using highly engineered MNCs instead of microbeads.

Considering potential biological applications of MADONAs, our nanorotors are an innovative and promising class of magnetic torque nano‐probes that combine three unique features. First, the torque values a single rotor can exert with only four MNCs are adequate to stall and manipulate molecular motors such as the F_0_F_1_‐ATPase, requiring ≈ 40 pN nm [94]. Second, our MADONAs can target and manipulate single molecules/receptors on membranes, here showcased by docking/rotating on a single biotin‐streptavidin pivot complex. Third, due to their strong magnetic responsiveness and high anisotropy, they can be manipulated in different magnetic modes at single‐digit mT field strengths, a field regime that can be achieved with a pair of low‐cost permanent magnets. Notably, under low‐viscosity conditions (η ≈ 1 mPa s) comparable to biological settings, we observed driven rotation of MADONAs at frequencies up to 40 Hz at 9 mT (Figure S34), highlighting their potential for high‐speed actuation in future biological and cellular applications.

## 3 Conclusions

We present nanoscale magnetic DNA origami nanoactuators that are able to exert biologically relevant magnetic torques at single‐digit mT magnetic fields. This is achieved by patterning highly magnetic and anisotropic custom MNCs on rigid 6HB DNA origami rotors. Our TIRFM‐RMF setup enables programmable nano‐rotation assays, in which the torque values were determined by balancing the drag and magnetic torques. By comparing two different rotor designs, differing in particle population and inter‐particle distance, we found that quantitative binding of MNCs on the 6HB origami and thus adequate torques are achieved when the inter‐binding site (particle distance) is set to more than one particle size. At this separation, there is a good tradeoff between quantitative binding and dipole‐dipole interactions between MNCs. Furthermore, Monte Carlo simulations show that attaching highly magnetic MNCs at 64 nm particle center‐to‐center distance leads to collective magnetic couplings and increased effective magnetic moment of the rotors. Our numerical simulations back up our TIRFM‐RMF assays with a good agreement between experimental and numerical torque values. Our 4‐MNC MADONAs can generate torques sufficient to stall and manipulate rotary molecular motors such as F_0_F_1_‐ATPase.

Endowing DNA origami with magnetic properties by assembling bespoke MNCs on them will lead to innovative magnetic nanoactuators that can be driven into several distinct configurations within milliseconds in response to time‐varying magnetic fields. Here, we show that such fast varying dynamics can be traced at the single rotor level with TIRFM by tracking several dyes. In terms of understanding magnetic movements, by harnessing the binding addressability of DNA origami, our MADONAs are an ideal platform to explore and understand how complex magnetic movements can be achieved depending on the particles properties and assembly of magnetic field setup. Our study demonstrates a proof‐of‐concept of genuine magnetic nanorotors and sets a solid foundation for working toward new class of biorthogonal nanoactuators in which the actuation is truly decoupled from the structure.

## Associated Content

Supporting Information (SI) contains: Supplementary Figures S1–S34 and Supplementary Tables S1–S4. Further contains: Supplementary Sections S1–S14 including: Materials and Methods, Synthesis Procedures, Description of rotor's rotation dynamics, Discussion on magnetic torque determination from frequency‐resolved drop‐out analyses on 4‐MNC MADONAs, the phase‐locking value, and MMC simulations.

## Author Contributions

A.L. and J.T. conceived the research idea. L.J.K.W. prepared DNA origami bundles, performed TIRFM measurements, analyzed data, conceptualized the dropout study and the synchronization model, simulated the rotor trajectories, and wrote the manuscript. F.R. designed and folded DNA origami bundles, performed TIRFM magnetic assays, analyzed data, and participated in the writing of the manuscript. Y.W. polymer coated and DNA labelled MNCs, performed magnetic and colloidal measurements, built Helmholtz coils, and analyzed particle characterization data. C.P. synthesized DNA origami, performed MNC binding to DNA origami, built Helmholtz coils, and performed magnetic actuation assays. X.Y. folded DNA origami and performed particle binding assays and TEM imaging. K.L. performed initial magnetic actuation assays. R.A. synthesized MNCs. T.T. prepared DNA origami structures, performed TIRFM assays and helped with the measurement setup. S.K performed TEM imaging. J.L. analyzed data. T.L. analyzed data and provided resources. F.C.S. analyzed data and provided resources. J.T. conceptualized, analyzed data and participated in the writing of the manuscript. A.L. developed MNCs and DNA labelling chemistries, performed MMC simulations, analyzed data, supervised the study, and wrote the manuscript.

## Funding

German Research Foundation: LA 4923/3‐1, TA1375/2‐1, SFB1032 (Project ID 201269156 TPA2 and TPA6). Germany's Excellence Strategy—EXC 3092/1 ‐ BioSysteM (Project No. 533751719). European Research Council: ProForce (101002656). BMBF: 6G‐life initiative. Free State of Bavaria: ONE MUNICH Project Munich Multiscale Biofabrication. Konrad‐Adenauer‐Stiftung (KAS).

## Conflicts of Interest

The authors claim no conflicts of interest.

## Code Availability

The source code of the data analysis routines and simulation files employed in this study is available from the corresponding authors upon reasonable request.

## Supporting information




**Supporting File**: adma74245‐sup‐0001‐SuppMat.pdf.

## Data Availability

Source data and all other data that support the plots within this paper and other findings of this study are available from the corresponding authors upon reasonable request.
