# Supplementary material for: Magnetic DNA Origami Nanorotors
Source: Adv Mater. 2026 Jul 30;38(52):e74245. doi: 10.1002/adma.74245 (PMC13579214; doi:10.1002/adma.74245)
Supplement: Supplementary file 1 [file ADMA-38-e74245-s001.pdf]

Supporting Information for: Magnetic DNA origami nanorotors

Lennart J. K. Weiß[1,‡], Florian Rothfischer[1,‡], Yihao Wang[2,‡], Christoph Pauer[3], Xin Yin[3], Kevin Lang[3], Rabia Amin[2], Thomas Tsalos[1], Susanne kempter[3], Jan Lipfert[4], Tim Liedl[3], Friedrich C. Simmel[1], Joe Tavacoli[3,*], Aidin Lak[2,*]

[1]Department of Bioscience, TUM School of Natural Sciences, Technical University Munich, 85748 Garching, Germany
[2]Institute for Electrical Measurement Science and Fundamental Electrical Engineering and Laboratory for Emerging Nanometrology (LENA), Technische Universität Braunschweig, 38106 Braunschweig, Germany
[3]Department of Physics, Ludwig Maximilians Universität München, Geschwister-Scholl-Platz 1, 80539 Munich, Germany
[4]Institute of Physics, University of Augsburg, 86159 Augsburg, Germany

* Corresponding authors: a.lak@tu-braunschweig.de, pholise@gmail.com

‡ These authors contributed equally to this work.

Contents

1. Synthesis, DNA labeling, and size fractionation of colloidal magnetic nanocubes 3
2. Design, folding and analysis of DNA origami structures 5
3. Binding of MNCs to rotors on surface and in solution 8
4. Structural and magnetic characterization of colloidal MNCs 9
5. Additional experimental data for multi-MNC MADONAs 11
6. Analysis of MNC binding to MADONAs 14
7. Co-rotation as phase-locking phenomena 17
8. Intrinsic energy landscapes of 4-MNC MADONAs 22
9. Rotor trajectories used in TIRFM-RMF assays 23
10. Magnetic torques of 4-MNC MADONAs from frequency-resolved drop-out analyses 28
11. Trap stiffness data for 4-MNC MADONAs 32
12. Monte Carlo simulations of magnetic properties of MADONAs 39
13. 4-MNC MADONAs driven in a low-viscosity buffer 43
14. Scaffold and staple sequences 44

References 54

1. Synthesis, DNA labeling, and size fractionation of colloidal magnetic nanocubes

Magnetic nanoparticle synthesis

Cobalt and zinc-doped iron oxide nanocubes (MNCs) were synthesized via thermal decomposition of iron (III) acetylacetonate, cobalt (II) acetylacetonate, and zinc (II) acetylacetonate in a mixture of dibenzyl ether, 1-octadecene, oleic acid, and sodium oleate at $290\,^{\circ}\mathrm{C}$ for 30 min as previously described [1].

Functionalization of poly(maleic anhydride-alt-1-octadecene) with NH_2-PEG(3)-azide linker

Our custom-synthesized poly(maleic anhydride-alt-1-octadecene) (PMAO) was modified with NH_2-PEG(3)-azide via anhydride ring opening reaction following the procedure published by Lak et al. and Jin et al. [2, 3]. In a typical reaction, $338\,\mathrm{mg}$ ($0.964\,\mathrm{mmol}$ monomer units) of PMAO were added into $17\,\mathrm{mL}$ dimethylformamide (DMF) in a $50\,\mathrm{mL}$ round flask and heated up to 65 °C. Next, $191.3\,\mu\mathrm{L}$ ($0.964\,\mathrm{mmol}$) of NH_2-PEG(3)-azide dissolved in $3\,\mathrm{mL}$ of DMF was pipetted into the reaction flask after the PMAO was completely dissolved. Afterwards, the mixture was left to react for 24 h at 65 °C. After cooling to room temperature, the crude lightly yellowish oily product was dialyzed against dichloromethane (DCM) using regenerated cellulose dialysis tubes (6 kDa cutoff size) to remove unreacted ingredients and byproducts. Finally, DCM was thoroughly removed in a rotary evaporator and the resulting polymer was dissolved in chloroform to obtain a final concentration of 35 mg (polymer)/ml and stored at 4 °C prior to usage.

MNC Polymer coating procedure

The polymer coating of oleic acid coated MNCs was performed and modified based on the protocol by Pellegrino et al.[4] and William W. Yu et al.[5]. Typically, $2.635\,\mathrm{mL}$ of PMAO-azide polymer (35 mg/mL) were diluted with $7.365\,\mathrm{mL}$ chloroform in a $30\,\mathrm{mL}$ glass vial and sonicated for $10\,\mathrm{min}$. Next, $2\,\mathrm{mL}$ of particle suspensions in chloroform ($1.44\,\mathrm{g}$(MNC)/L) were first diluted with $8\,\mathrm{mL}$ of chloroform and then added dropwise into the polymer solution during sonication for $10\,\mathrm{min}$ to homogenize the mixture. To ensure the homogeneous and efficient intercalation of PMAO-azide polymer hydrocarbon chain with oleic acid on NPs, the mixture was stirred and incubated at room temperature overnight. The ratio of polymer to MNCs corresponds to 500 polymer units per nm^2 of a particle. Next, chloroform was removed very slowly within 5 -6 h through stepwise reduction of pressure to a final value of $350\,\mathrm{mbar}$ and increase of temperature to 34 °C. After a complete removal of chloroform, particles were resuspended in $20\,\mathrm{mL}$ of sodium borate buffer (pH 8.7) by sonication for $1\,\mathrm{h}$ at 45 °C. The particle suspension was then concentrated to $2\,\mathrm{mL}$ using spin filtration (Amicon regenerated cellulose spin filter, $15\,\mathrm{mL}$, $30\,\mathrm{kDa}$ cutoff size) at $3200\,\mathrm{rpm}$ and 20 °C for $20\,\mathrm{min}$.

Particle purification and fractionation

To remove polymeric micelles and partile clusters that are formed during the polymer coating, we fractioned MNCs on non-continuous sucrose gradient ($10\,\%$:$40\,\%$:$60\,\%$, from top to bottom, each fraction $4\,\mathrm{mL}$) in centrifuge falcon tube. Typically, 500 µL of polymer-coated particle suspensions in borate buffer were loaded into the sucrose centrifuge tube and centrifuged for $1.5\,\mathrm{h}$ at $4500\,\mathrm{rpm}$ at 4 °C. Next, to collect monomeric MNCs, meaning nanoparticles that are singly coated within a polymeric shell, nanoparticles between $10\,\%$ and $40\,\%$ band, including $10\,\%$ band, were collected using a long needle. The sucrose was then removed by 3 rounds of Amicon filtration (Amicon spin filter, $15\,\mathrm{mL}$, $30\,\mathrm{kDa}$ cutoff size) at $3000\,\mathrm{rpm}$ and $20\,^{\circ}\mathrm{C}$ for $15\,\mathrm{min}$. After each round of spin filtration the particles were thoroughly resuspended in borate buffer by vigorous pipetting. Finally, the particles were concentrated to $2\,\mathrm{mL}$ in TE ($5\,\mathrm{mmol}$ Tris, $1\,\mathrm{mmol}$ EDTA, $5\,\mathrm{mmol}$ NaCl, pH 7.3) buffer by spin filtration. To further remove any nonmagnetic impurities from the particles, we performed one round of magnetic washing using a $1.5\,\mathrm{mL}$ MACS column (Miltenyi Biotec) in combination with a MiniMACS separator (permanent magnet) as previously described [2].

Agarose gel electrophoresis to purify polymer coated NPs

To extract the fraction of monomeric MNCs from the mixture of polymer coated particles, native agarose gel electrophoresis (AGE) was applied. In a typical preparation procedure, $1.5\,\%$ agarose gel in $1\,\times$ TAE running buffer was made by dissolving $0.5\,\mathrm{g}$ of agarose in 50 mL of $1\,\times$ TAE buffer by $2\,\mathrm{min}$ microwave irradiation. After cooling down at room temperature for $5\,\mathrm{min}$, the homogenous gel solution was casted on a gel mold and left to solidify for $30\,\mathrm{min}$. Afterwards, the casted gel was placed in electrophoresis chamber (Biorad) and samples were pipetted (containing $20\,\%$ of $40\,\%$ sugar solution) into pockets. Next, AGE was run at $90\,\mathrm{mA}$ for $35\,\mathrm{min}$ to separate the monomeric MNCs from the multimeric NPs. Finally, the front section (3-4 mm wide) of the running band that contains monomeric single coated MNCs was excised from the gel. The single particles were then collected by squeezing the gel fragment.

DNA labeling of MNCs

Via click-chemistry conjugation, we are able to label DNA single strands onto our MNCs. Here, we labelled our MNCs with 20 nt oligos(dT) that are complementary to 8 nt oligos(dA) on the binding sites of DNA origami. In a typical process, a 150 µL of MNC suspension in TE buffer at a particle concentration of 15 nM was mixed with 32.8 µl of 100 µM 5'-T(20)-DBCO-3' ssDNA in TE to obtain the nominal grafting density of 0.12 ssDNA/nm². After mixing, the sample was incubated at room temperature overnight. To maximize the binding efficiency of ssDNA to MNCs, we applied a so-called salt aging procedure using 5 M NaCl salt the next day. We increased the concentration of NaCl in the mixture incrementally by 100 mM every hour up to 400 mM. After each salt adjustment step, the mixture was homogenized by vortexing and sonicating for 20 s. At the end of the salt aging procedure, the mixture was again incubated at room temperature overnight. On the next day, the excess of ssDNA was washed out by 2 rounds of centrifugation at 13000 rcf at 5 °C for 12 min. Finally, the sample volume was adjusted back to 150 µl to obtain the original particle concentration. The samples were stored at 4 °C prior to further use.

2. Design, folding and analysis of DNA origami structures

DNA origami rotor designs

6HB DNA origami structures were designed using cadnano 2.5 [6]. The $7,249$ bases long scaffold was derived from the M13Mp18 bacteriophage genome (produced in house, also available from tilibit nanosystems GmbH). Figure S1 shows a schematic of the two designs used in the study. Detailed cadnano designs of the 4-MNC and multi-MNC design are shown in Supplemental Figures S2 and S3. Sequences of scaffold and all the staples can be found in Supplementary Note 14 Scaffold and Staple sequences. Further accompanying structure design files are also available online at nanobase.org.

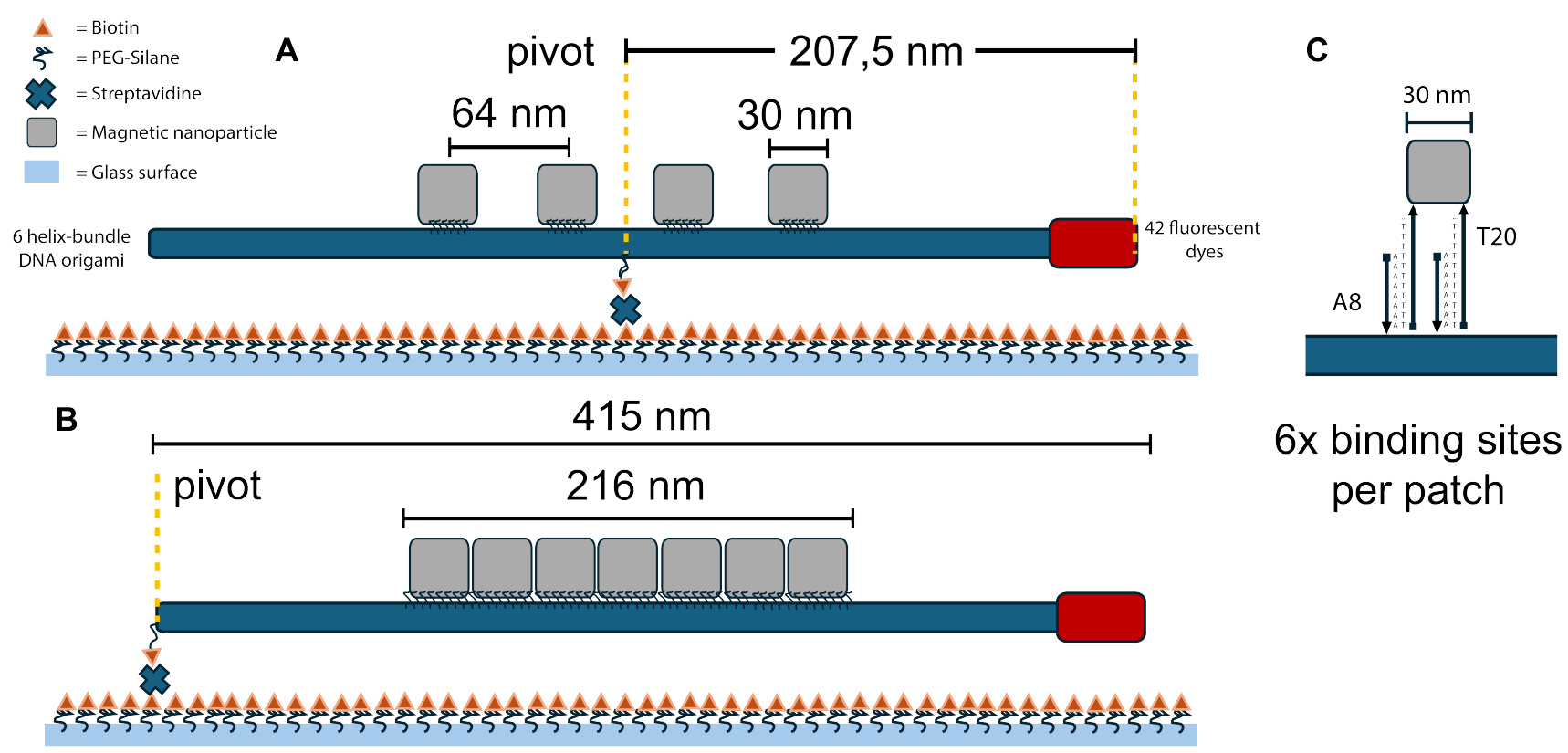

FIG. S1. **Schematic presentation of 4-MNC and multi-MNC MADONAs dimensionality.** Two schemes to visualize the dimensions of **(A)** 4-MNC and **(B)** multi-MNC MADONAs. For the 4-MNC version four MNC binding patches are arranged on the 6HB DNA origami. Each patch consists of 6 staples with 8-nt Poly-A overhangs distributed over a distance of 17 nm, with a center-to-center distance of 64 nm. For the experiments with endpivot and multi-MNC a continous binding site with a total of 96x 8-nt Poly-A extensions was used covering a length of roughly 216 nm length. The full length of the 6HB is 415 nm in both cases. Further shown are the binding scheme to the glass surface via a Biotin-Streptavidin bond and the 42x Atto-655 fluorescent dyes at the right side tip of the DNA origami.**C** Close up of the intended ideal binding of the 8-nt overhang from the origami staple to the 20-nt oligo bound to the MNC.

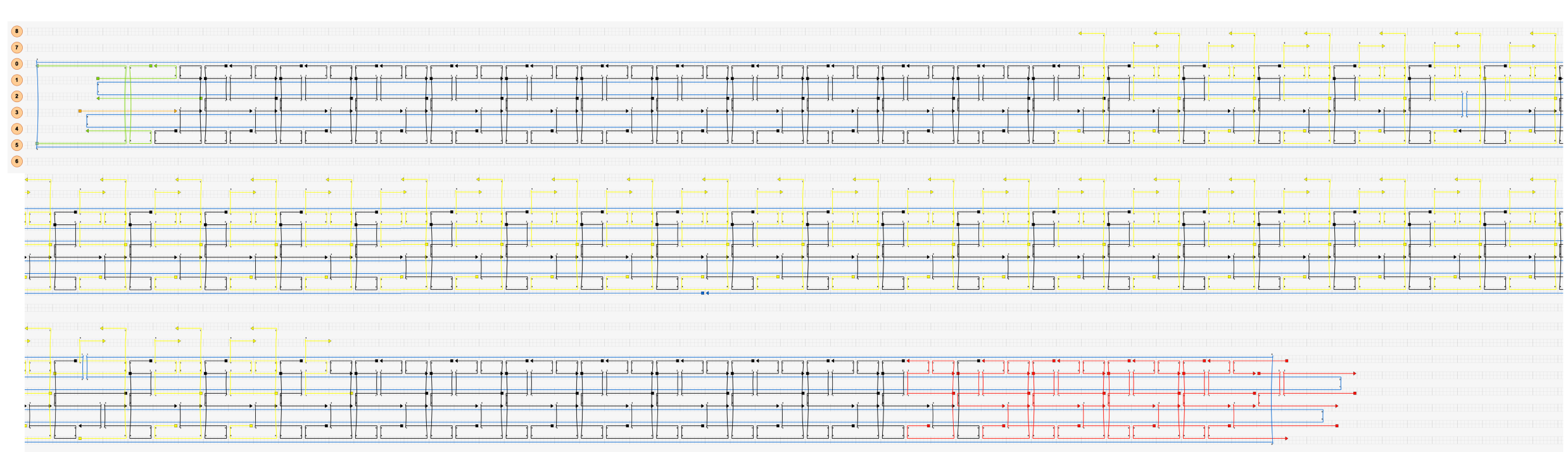

FIG. S2. **DNA origami design of multi-MNC MADONAs**. Staple colors show special functionalities. Black = unmodified basic staples. Red = Staples with extension sequence to bind fluorescent dyes. Green = blunt end staples. Yellow = Staples extended 8-nt poly-A sequence to bind MNCs. Orange = Biotin-modified staple.

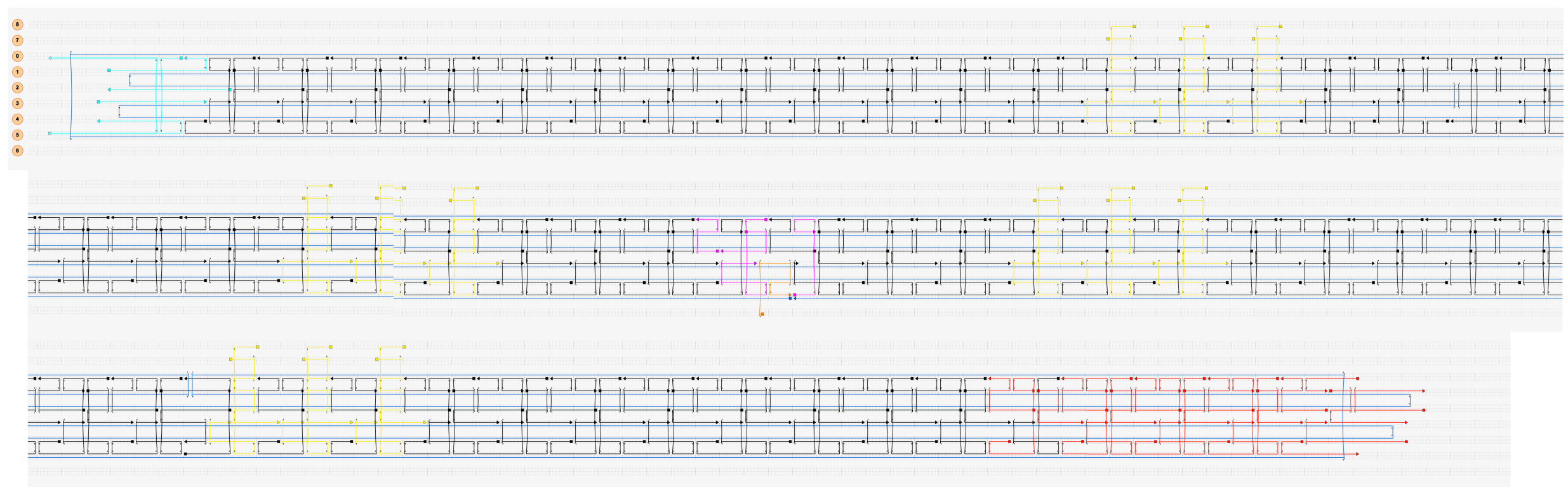

FIG. S3. **DNA origami designs of 4-MNC MADONAs**. Staple colors show special functionalities. Black = unmodified basic staples. Red = Staples with extension sequence to bind fluorescent dyes. Cyan = Passivated end design of 6hb. Yellow = Staples extended 8-nt poly-A to bind MNCs for structures with 4 MNCS. Magenta= modified staple routing for mid pivot. Orange = Biotin-modified staple.

DNA origami folding and purification

The rotor samples were folded using $100\,\mu\mathrm{L}$ of $100\,\mathrm{nM}$ scaffold (in-house produced in ddH_2O) combined with a $5\,\times$ molar excess of staples (obtained from IDT at $100\,\mu\mathrm{M}$ in 1x IDTE buffer pH 8.0) over the scaffold.

Both the multi-MNC and 4-MNC structures were folded and purified using the same protocol. The only difference being the added staple modifications and pivot point placement.

Sample buffer was adjusted to 1xTE, $12\,\mathrm{mM}$ $MgCl_2$ and $5\,\mathrm{mM}$ NaCl for folding. Folding was performed by a temperature ramp from 70 to $40\,°\mathrm{C}$ over a duration of 10 hours using a thermal cycling device. The temperature was reduced by $0.1\,°\mathrm{C}$ every $2\,\mathrm{min}$ during the thermal ramp.

Rotor samples were purified in a step-wise manner. Folded samples were at first purified from excess staples using PEG-precipitation similar to reference [7]. Afterwards Neutravidin and fluorescent dyes were added in $10\,\times$ molar excess over the initial scaffold concentration of the sample during folding. Lastly, a agarose gel electrophoresis ($70\,\mathrm{V}$, $1\,\mathrm{hour}$, $0.5\ \times\ TBE$, $5\,\mathrm{mM}$ $MgCl_2$ running buffer) was performed to separate rotor structures from Neutravidine and excess dye, followed by gel extraction to recover the structures from the gel (cf. reference [8]). Extraction from the gel was performed based on the fluorescence from the added Atto655 dyes at the tip of the 6HB.

Origami structure verification

For the structural verification of the functional assembly between MNCs and DNA origami rotors we used TEM. Negative stain-TEM micrographs were recorded similar to our previous studies (cf. reference [9]), with the addition of a 2-10x excess of MNCS over DNA origami rotor binding sites on the TEM grid. The TEM used for acquisition was a Philips CM-100 at $100\ kV$ and samples were applied to FCF400-CU copper grids from Electron Microscopy Sciences.

TIRFM and TIRFM-MF measurement setups

The TIRF microscope setup used in this study is the same as in reference [8]. The sample chambers used for fluorescence measurements were similar to previous study [10] and formed a $50\,\mu\mathrm{m}$ high channel of $12\,\mathrm{mm}$ length.

Static and rotating magnetic fields (RMFs) were generated using two pairs of custom-made pseudo-Helmholtz coils (0.69 mm, 110 windings), which were placed in a cross-wise arrangement around the TIRFM flow cell. This configuartion enbales exerting homogeneous static magnetic fields along a single axis and in x-y plane RMFs. Fields were generated using an arbitrary waveform generator (TGA1244, TTi, UK) and a pair of bipolar current amplifiers (BOP 20-10DL, Kepco Inc., USA).

Analysis of single-molecule fluorescence measurements

Fluoresecence data acquired using our single-molecule TIRF microscopy setup tracking measurements was processed using the *Picasso* software package from reference [11]. In all of the data acquisitions, we used the 42 fluorescent dyes at the tip of the rotor structure for single-molecule structure tracking. Therefore the combined fluorescence of all dyes was used for the positional localization of the rotor structures during every timestep of the measurement. If movements of the sample or sample stage changed the emitter position during measurements, a drift correction function of the *Picasso* package was applied to realign the datapoints.

In a next step the data was imported for further analysis using a custom python analysis package *roboarmanalysis* [12]. In that routine the center of each rotor was determined using a circular fit of the acquired data. This was achieved using a circular fit. In a next step, the positional coordinates of all localizations were transformed into relative polar coordinates per rotor. Rotor trajectories were generated by plotting the normalized rotor position as a function of average time.

3. Binding of MNCs to rotors on surface and in solution

Binding of DNA origami to TIRFm flow cell

For measurements the rotor structures were pipetted onto a PEG-modified glass slide (PEG modification protocol cf. reference [10]) at a concentration of $200\,\mathrm{pM}$. The measurement buffer used during the fluorescence acquisitions was $0.3\times$ TB ($30\,\mathrm{mM}$ Tris, $30\,\mathrm{mM}$ Borate), $3\,\mathrm{mM}$ $MgCl_2$ in deionized H_2O with 48 % sucrose.

After the sample was applied to the PEG-biotin surface and incubated for 30 s, the chamber was washed with at least 1 mL of the measurement chamber.

Assembly of MNCs on rotors on surface directly in TIRFM measurement flow cell

For TIRFM measurements the MNCs were added to DNA origami rotors already bound to the PEG-Biotin surface of the microscopy flow cell. The MNCs were added in excess to the flow cell by flushing of $10\,\mathrm{\mu L}$ ($10\,\mathrm{nM}$ MNCs in $1\times$ TE $20\,\mathrm{mM}$ $MgCl_2$) in the measurement chamber. The DNA origami sturctures and MNCs were incubated for 30 minutes at RT in the flow cell. For the measurement, the chamber and wells were filled with measurement buffer and buffer was refilled regularly to restrict evaporation caused changes in the sample chamber during elongated measurement times.

Assembly of MNCs on rotors on surface on functionalized mica surface

AFM data were acquired using an Asylum Research Cypher ES (Oxford Instruments) equipped with Bruker FastScan-D cantilevers operated in AC mode. To reproduce the stepwise assembly procedure used in the TIRFM flow-cell experiments as closely as possible, BSA-biotin, 4-MNC MADONAs, and MNCs were sequentially incubated on freshly cleaved mica. However, the assembly was performed in $1\times$ TE supplemented with 500 mM NaCl instead of high $MgCl_2$, thereby minimizing additional surface-bound sinks that could compete with binding to the MADONAs and interfere with assembly. Briefly, 30 µL of BSA-biotin solution (1 mg mL^{-1}) was deposited onto the mica surface and incubated for 5 min. Following thorough washing, 10 µL of 200 pM 4-MNC MADONAs was added and incubated for 3 min. After a second washing step, 10 µL of 20 nM MNCs were added and incubated for 30 min, during which the sample was periodically mixed by gentle pipetting. After the assembly, the buffer was exchanged to an AFM imaging buffer consisting of $1\times$ TE, 10 mM $MgCl_2$, and 10 mM $NiCl_2$ and images were acquired at a scan rate of 3 Hz.

Assembly of MNCs on rotors in solution for statistical analysis

6HB DNA origami rotors of 1 nM concentration were mixed with MNC suspensions 5 nM to achieve 5 times particle excess over each MNC binding site origami. The $MgCl_2$ concentration was adjusted to a final concentration of 15 mM during the assembly. The mixture was then placed in a thermocycler and the following thermal annealing program was applied for 3 times: Warming up to 45 °C and cooling down to 25 °C at a cooling rate of 0.1 °C per min. Afterwards, the mixture was pipetted in loading pocket of a 1 % agarose gel to purify assembled rotors from unbound MNCs and DNA origami. The agarose gel electrophoresis ran at 80 V for 90 min. The assembled rotors were recovered from the agarose gel by cutting and squeezing their faint, yet specific, black band that runs slower than the band of free MNCs or origami in the gel.

4. Structural and magnetic characterization of colloidal MNCs

Dynamic light scattering (DLS)

DLS measurements were performed using a Malvern Zetasizer instrument at 173° backscattered measurement mode. Measurements were performed on 70 µL of particle suspensions in TE buffer (pH 7.3) at a typical particle concentration of 0.015 - 0.02 g/L at 295 K.

Magnetic hysteresis loops: characterization of saturation magnetization, magnetic coercivity, and magnetic anisotropy

The magnetic hysteresis loops were measured using magnetic property measurement system (MPMS, Quantum Design). The samples were prepared by pipetting a 100 µL suspension of CMPs in a 6 cm NMR tube. The hysteresis loops were recorded at 298 K using DC scan mode over magnetic fields between -7 and 7 T. The applied magnetic fields were corrected for a remanence field remaining in the chamber and the magnets by referencing measurements against a palladium standard sample. The coercive fields were corrected accordingly.

Determination of effective magnetic anisotropy constant

The effective anisotropy constant K_{eff} is determined using the modified Stoner-Wohlfarth (SW) model proposed by Garcia-Otero et al.[13] The model considers MNCs with uniaxial magnetic anisotropy, randomly distributed magnetization easy axes, and thermal fluctuations. In the classical SW model for randomly distributed MNCs, the coercive field holds H_{c} = 0.479 H_{a}, in which $H_{\mathrm{a}} = 2K_{\mathrm{eff}}/(\mu_0 M_{\mathrm{s}})$ is the anisotropy field. By taking effects of temperature into account via the Néel relaxation, the temperature-dependent H_{c} (T) is given by

$$\mu_0 H_c(T) = \mu_0 H_a(T)\left(0.479 - 0.81\left(\frac{k_B T}{2K_{eff}(T)V}\left(\ln\frac{\tau_m}{\tau_0}\right)\right)^{\frac{3}{4}}\right) \tag{1}$$

where K_{eff} is the effective anisotropy constant and $\mu_0 H_{\mathrm{c}}$ is linearly proportional to $T^{\frac{3}{4}}$ up to certain temperature (Fig. S4a). Assuming $\tau_0 = 10^{-9}$ s and τ_m=100 s, K_{eff} was computed and plotted over temperature by fitting $\mu_0 H_{\mathrm{c}}$ versus $T^{\frac{3}{4}}$ with Eq.1 (Fig. S4b).

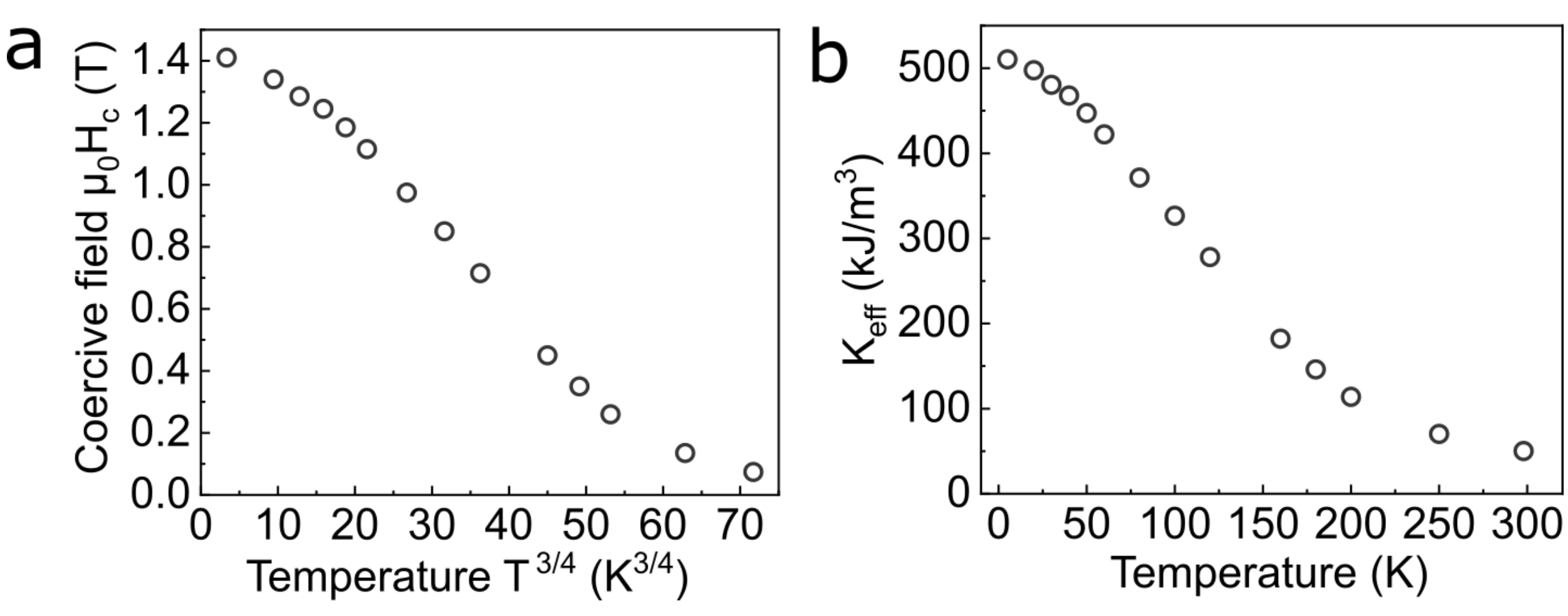

FIG. S4. **Experimental determination of the effective magnetic anisotropy constant**. (a) Coercive field (H_{c}) as a function of temperature measured over a broad range of temperture. (b) K_{eff} versus temperature.

Particle size analysis

Particle size histograms of uncoated (oleic acid coated) and polymer coated MNCs were obtained from the analysis of transmission electron microscopy (TEM) images (JEOL, 100 kV). Negative-stain TEM images were obtained via staining with 1 % uranyl formate, as described in our previous work [2].

5. Additional experimental data for multi-MNC MADONAs

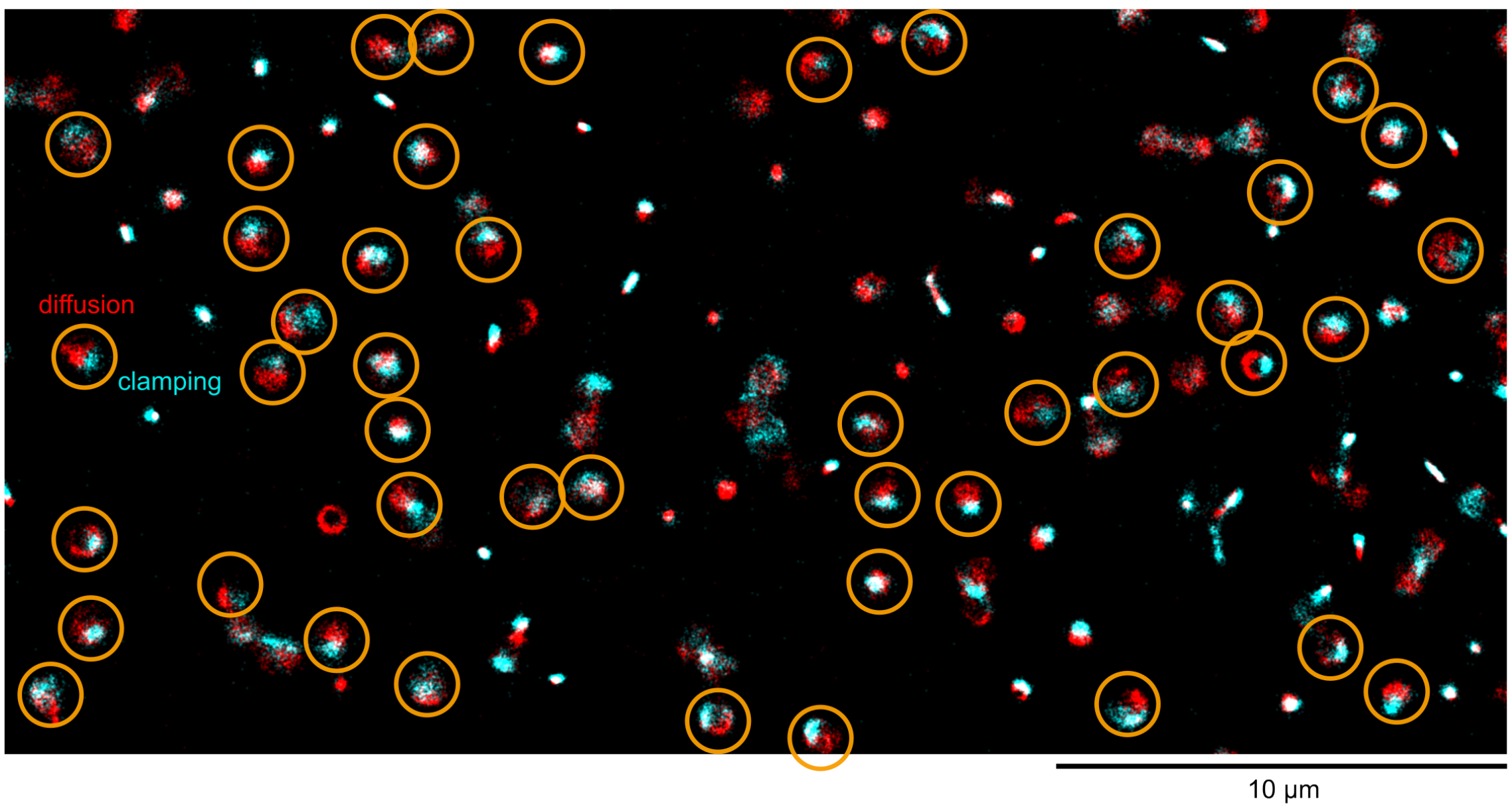

FIG. S5. **Raw localizations for a Multi-MNC MADONA ensemble**. False color representation of rotor localizations during diffusion (red) and clamping (cyan) at 6 mT magnetic fields. The cirles indicate functional (non-sticky) multi-MNC MADONAs.

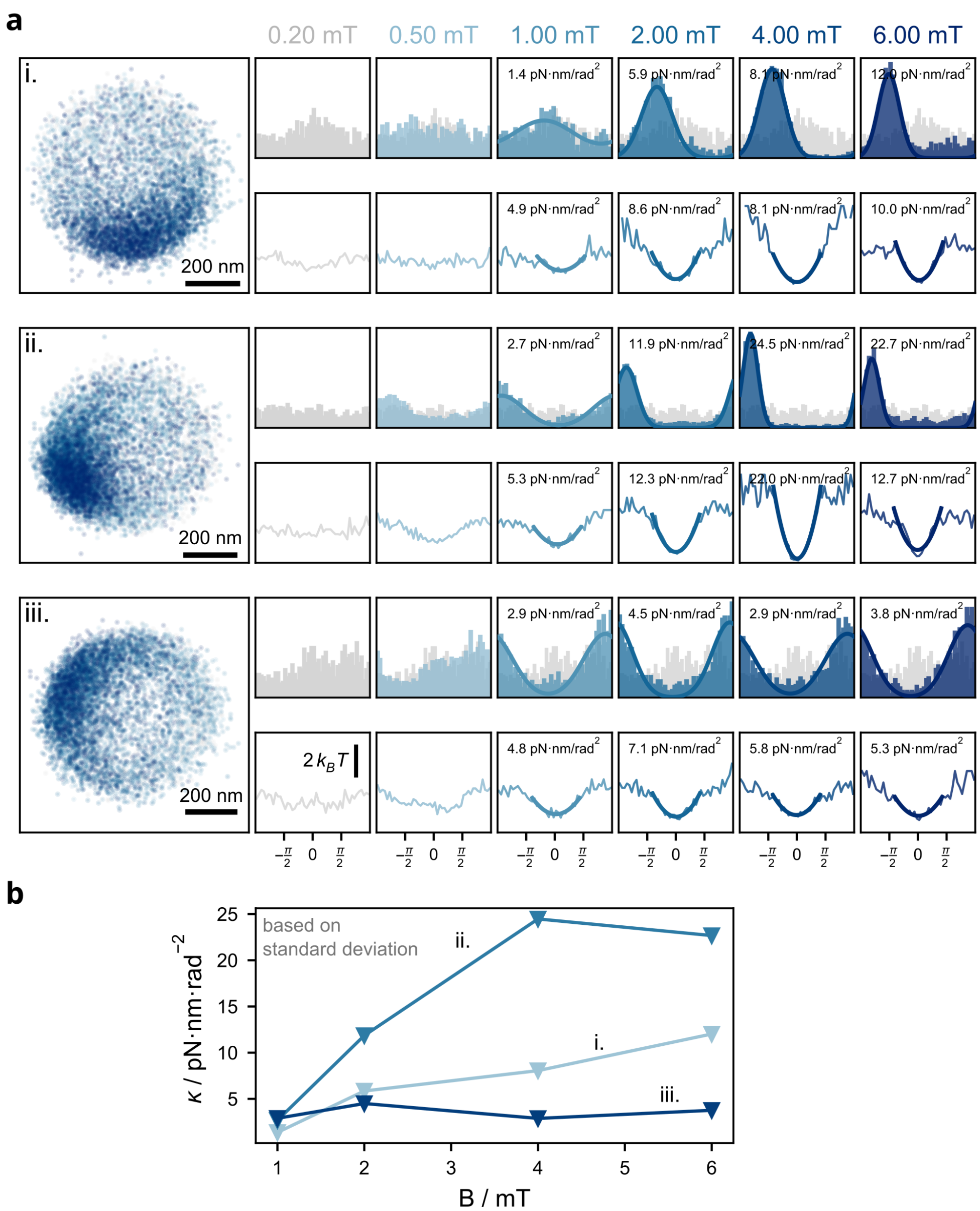

FIG. S6. **Trap stiffness of multi-MNC MADONAs during clamping experiment**. (a) Localization histograms (top row) and energy landscapes (bottom row) for three exemplary rotors (denoted as i., ii., and iii.) overlayed with a Gaussian or quadratic fit estimating the trap stiffness. (b) Angular trap stiffness κ as a function of field strength B based on the Gaussian fits.

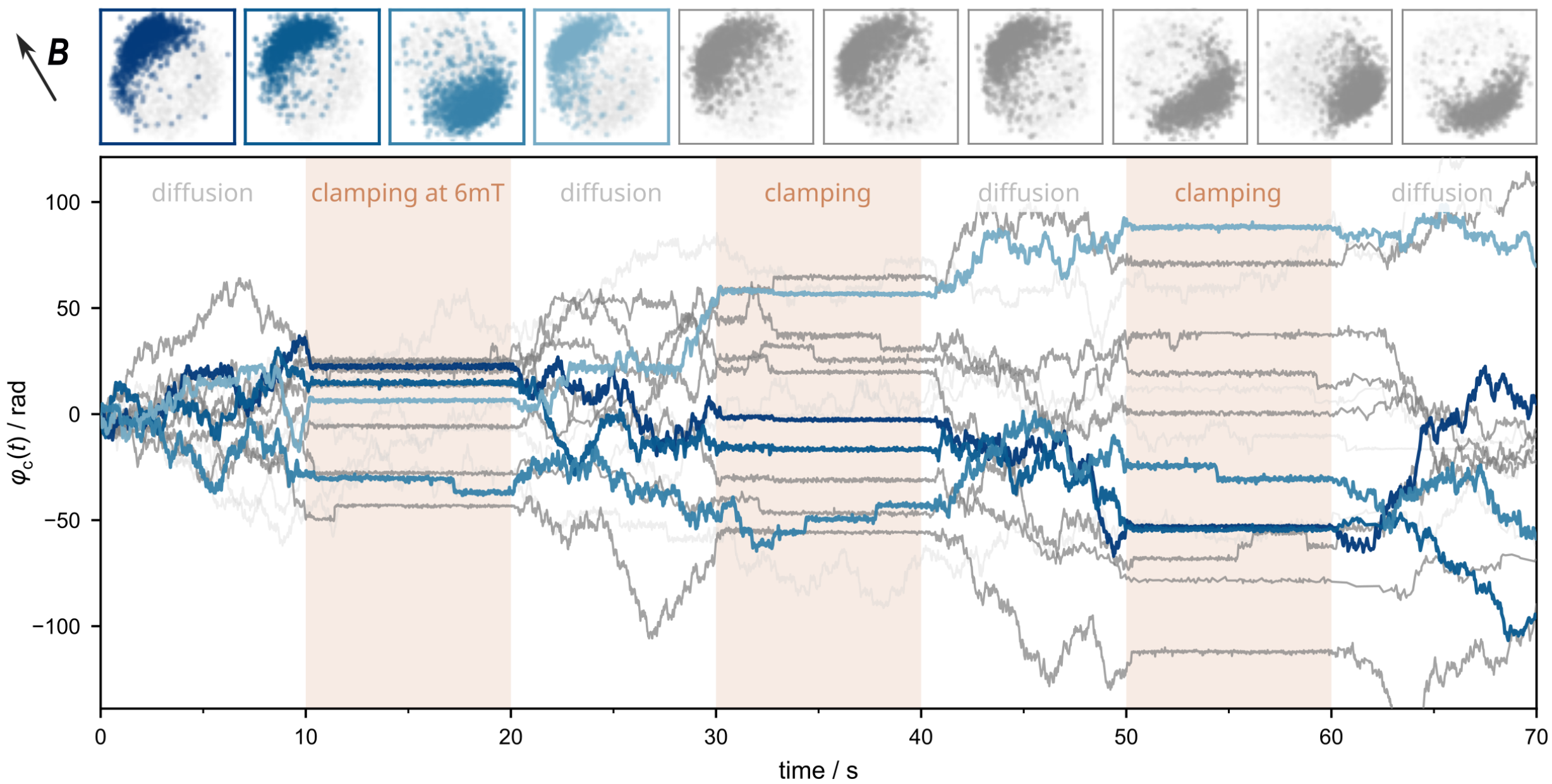

FIG. S7. **On/off magnetic clamping assays on multi-MNC MADONAs.** Localization scatter plots (top) and trajectories (bottom) from an experiment in which a magnetic field of $B = 9$ mT was repeatedly switched on and off in 10 s intervals. Scatter plots show diffusion-phase localizations in lightgray and clamping-phase localizations in highlighted colors. Shaded regions in the trajectory panel indicate clamping intervals. Rotors that were reliably trapped by the external field are plotted in darkgray, whereas trajectories from unresponsive rotors are shown in lightgray. The data was recorded in 1x TE, 500mM NaCl at 20 ms frame time.

6. Analysis of MNC binding to MADONAs

TEM micrographs of multi-MNC MADONAs

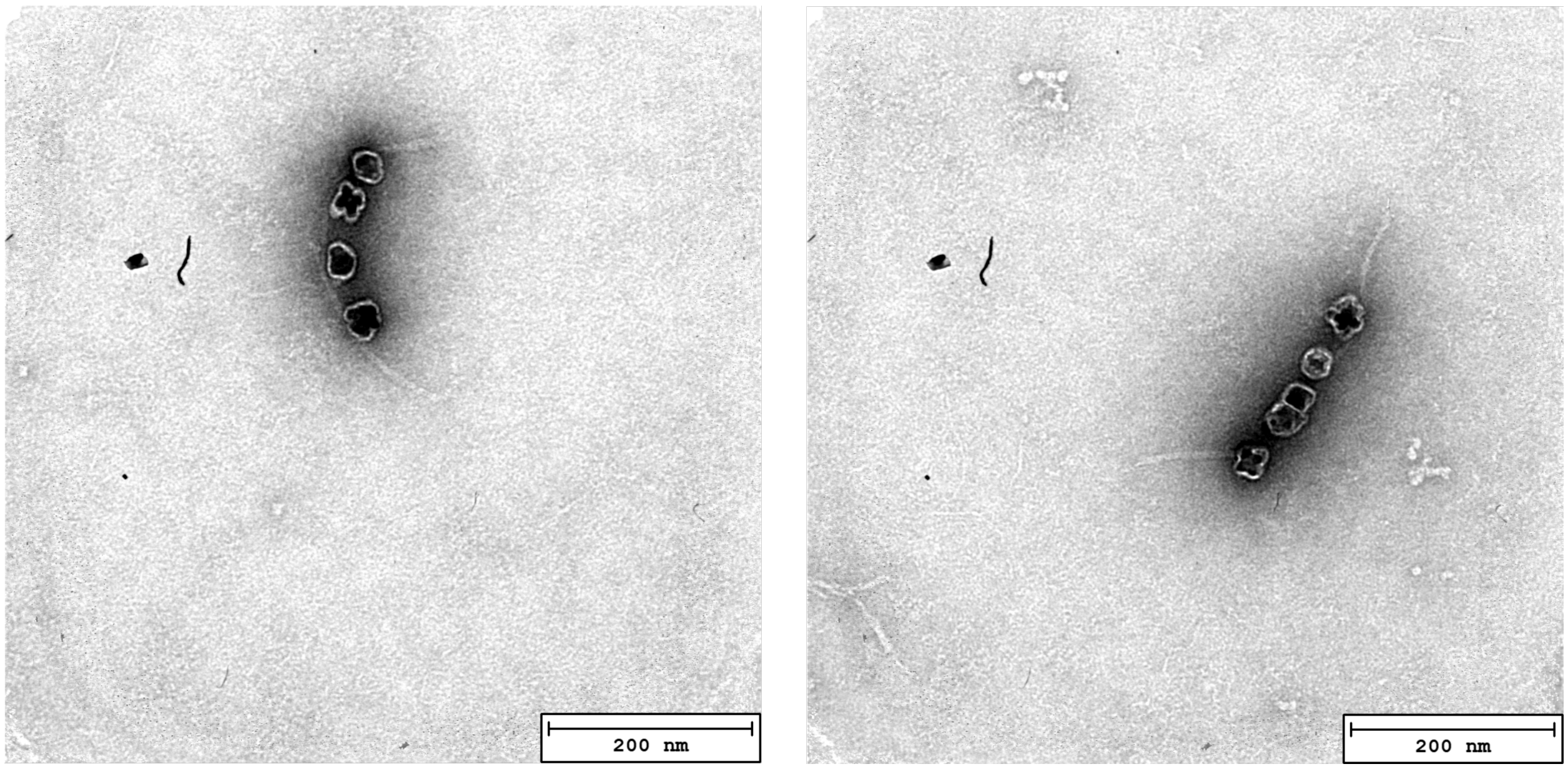

FIG. S8. **Negative-stain TEM micrographs of multi-MNC MADONAs**. These rotors were prepared through coupling of MNCs on the 6HBs in solution. The structures were then purified from unbound MNCs and 6HBs using excise-squeeze technique. In the multi-MNC design, the particle binding sites are placed 6-8 nm apart from each other, binding of several particles with the center-to-center distance of $\approx$ 17 nm are expected. This means the maximum number of MNCs per rotors is seven to eight. However, these TEM images show that, persumably due to the fact that the binding sites are very closed to each other, the actual number of bound MNCs per structure is far from ideal.

AFM images of 4-MNC MADONAs assembled on a mica surface

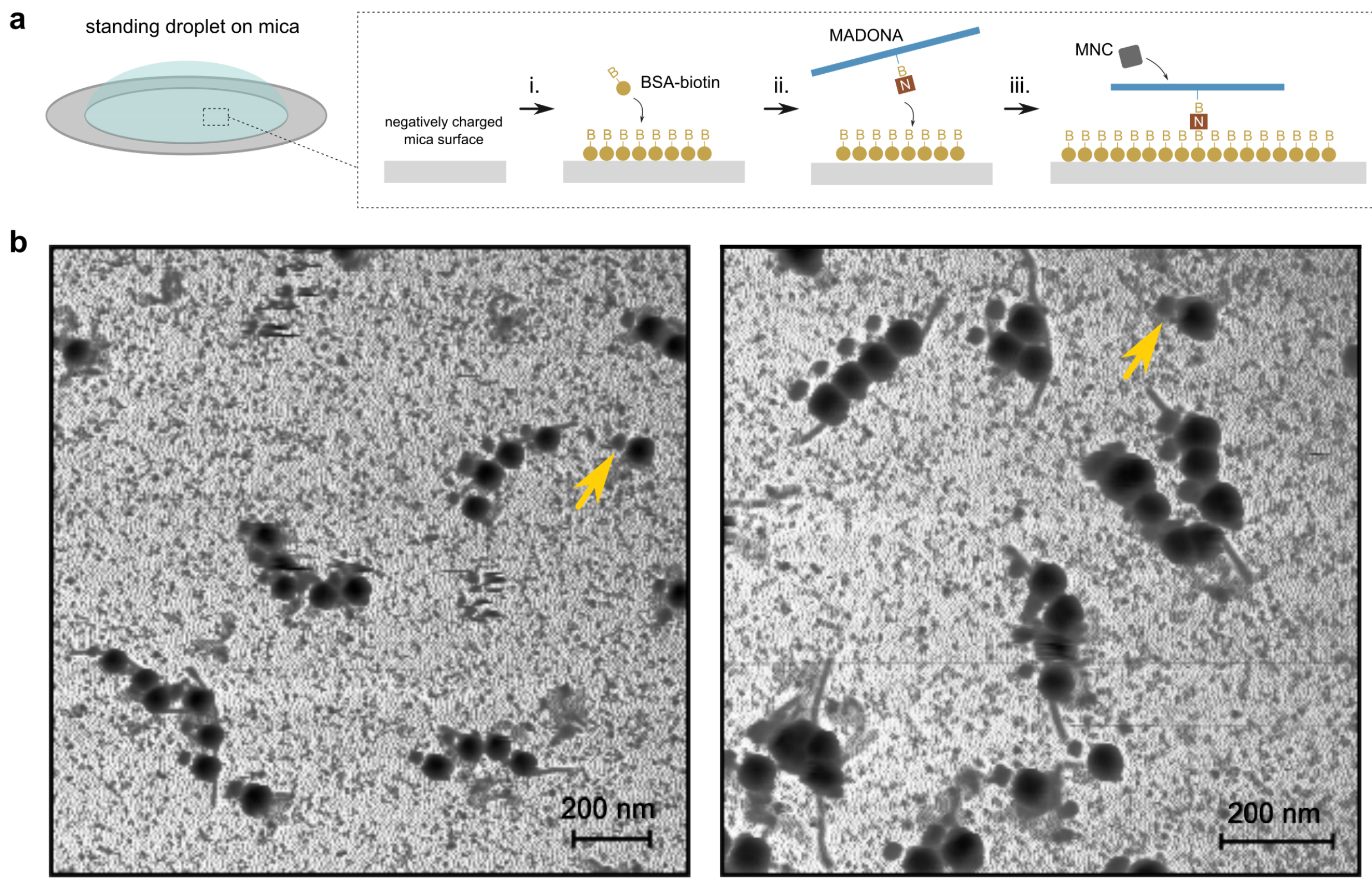

FIG. S9. **Assembly of MNCs on rotors on a functionalized mica surface.** (a) Assembly procedure on mica, mimicking the preparation performed in the flow cell for TIRFM measurements. i. Freshly cleaved mica was functionalized with biotinylated BSA to emulate the biotin-PEG surface used for TIRFM experiments. ii. MADONAs with 4 binding sites were incubated on the surface for 3 min. iii. MNCs were added in 500 mM NaCl, 1 × TE buffer and incubated for 30 min to promote attachment to the MADONAs. Each step was followed by thorough washing. (b) False-color AFM height images of two representative fields of view. Rotor aggregation and limited height contrast make reliable assignment of MNC occupancy challenging. Arrows indicate gray shadow artifacts appearing to the left of each MNC due to the AFM imaging process introducing the risk of false-positives in crowded areas.

TEM micrographs of 4-MNC MADONAs

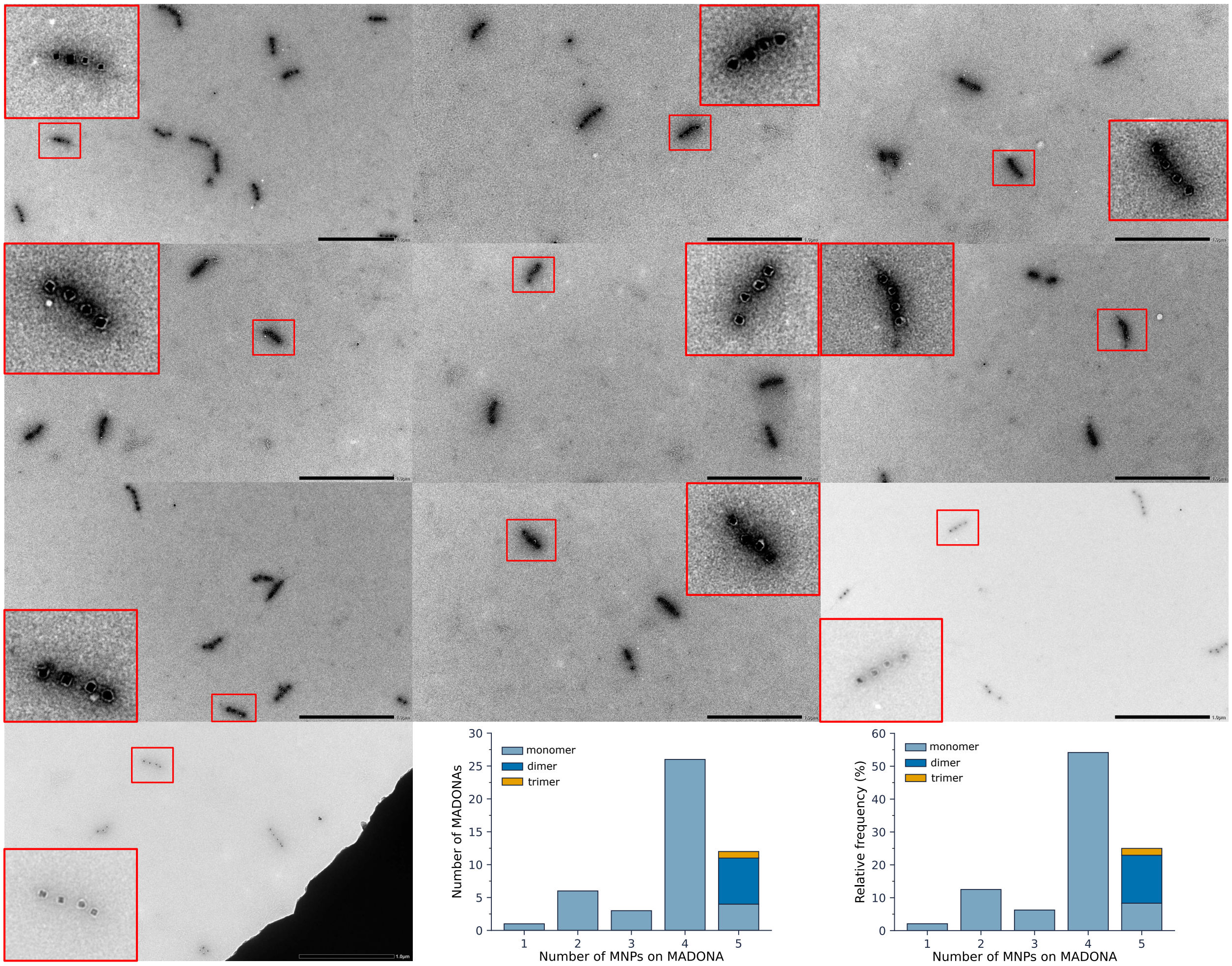

FIG. S10. **Collection of negative-stain TEM micrographs of 4-MNC MADONAs for statistical distribution analyses**. These rotors were prepared by coupling MNCs on the 6HBs in-solution. The structures were then purified from unbound MNCs and 6HBs using excise-squeeze technique. In the 4-MNC design, the particle binding sites are placed at 64 nm ($R_{\mathrm{c-c}}$) distance. Insets show MADONAs with 4 MNCs bound that are highlightd with red rectangulars. Histograms showing raw number and relative percentages of MNC distribution on MADONAs are shown in the bottom right (N = 48). Ensemble subpartitions which include a bound dimeric or trimeric MNC are marked dark blue and yellow in the histogram.

7. Co-rotation as phase-locking phenomena

We consider a DNA origami rotor with magnetic nanocubes rigidly attached to its structure, while each magnetic cube carries a magnetic dipole moment m_i. Then, the resulting magnetic moment of the rotor m_{eff} experiences a torque $\tau_m = m_{\mathrm{eff}} B \sin(\varphi_B - \varphi_m)$, depending on its axis' orientation φ_m relative to the external field φ_B. Since the magnetic particles are rigidly coupled to the DNA origami, the rotor co-rotates, and there is a constant angle offset between m_{eff} and the rotor axis, $\varphi_m = \varphi_r + \Delta\varphi$. For notational simplicity, we eliminate the constant phase offset by setting $\Delta\varphi = 0$, thereby virtually aligning the rotor axis with the axis of the magnetic moment. The rotor trajectory for a rotating magnetic field at constant angular velocity, $\varphi_B(t) = \omega t$, is described by a torque balance in the overdamped regime, where the induced magnetic torque has to compensate for the viscous drag, the local restoring torque stemming from rotor-surface interactions, and thermal fluctuations.

$$\zeta \dot{\varphi}_r + U'(\varphi_r) + \xi(t) = m_{\mathrm{eff}} B \sin\big(\omega t - \varphi_r\big). \tag{2}$$

Here, ζ is the drag coefficient, $U(\varphi_r)$ the energy landscape, and ξ Gaussian white noise with $\langle \xi(t)\, \xi(t')\rangle = 2\zeta k_B T\, \delta(t-t')$. We switch to a rotating-frame description by defining a phase offset between the magnetic field and the rotor's angular position,

$$\theta = \omega t - \varphi_r, \quad \dot{\theta} = \omega - \dot{\varphi}_r. \tag{3}$$

Then, substituting φ_r into torque balance yields

$$\dot{\theta} = \omega - \frac{m_{\mathrm{eff}}\, B}{\zeta} \sin\theta + \frac{1}{\zeta} U'\big(\omega t - \theta\big) + \sqrt{2D}\, \eta(t), \tag{4}$$

with a normalized noise term $\frac{1}{\zeta}\, \xi(t) = \sqrt{2D}\, \eta(t)$, D being the rotational diffusion coefficient. We further assume the energy landscape of the rotor to be $U(\varphi_r) = -\Delta E \cos(n\varphi_r)$, which leads to an expression for the surface-induced torque of $U'(\omega t - \theta) = n\Delta E \sin\big(n(\omega t - \theta)\big)$ and we arrive at

$$\dot{\theta} = \omega - \frac{m_{\mathrm{eff}}\, B}{\zeta} \sin\theta + \frac{n\Delta E}{\zeta} \sin\big(n(\omega t - \theta)\big) + \sqrt{2D}\, \eta(t). \tag{5}$$

Finally, denoting $K = m_{\mathrm{eff}}\, B/\zeta$ as the coupling strength and $\Delta E' = \Delta E/\zeta$ as the scaled surface-interaction potential, the phase obeys a generalized stochastic Adler equation

$$\dot{\theta} = \omega - K \sin\theta + n\Delta E' \sin\big(n(\omega t - \theta)\big) + \sqrt{2D}\, \eta(t), \tag{6}$$

where the positive $\Delta E'$-term represents the additional desynchronizing effect of the local surface interactions. Altogether, Eqn. (6) describes a non-autonomous stochastic Adler–type system – mathematically equivalent to an overdamped Brownian particle in a time-varying washboard potential. The system reveals regimes of sustained phase locking for strong coupling, intermittent phase slips, and thermally activated escapes at lower coupling strength relative to the detuning term ω.

Classical Adler equation

In the limit of a flat energy landscape ($\Delta E' \to 0$) and vanishing fluctuations ($\eta = 0$), Eqn. (6) reduces to the classic Adler equation,

$$\dot{\theta} = \omega - K \sin\theta, \tag{7}$$

which describes the deterministic synchronization (of a natural oscillator) to an external periodic drive. Since our system has no intrinsic frequency, the detuning term is simply ω. The rotors are phase-locked to the external drive, $\dot{\theta} = 0$, if the magnetic torque compensates the viscous drag. In the classic Adler equation, a fixed point exists whenever

$$|\omega| \leq K \quad \text{and} \quad m_{\mathrm{eff}}\, B \geq \zeta\, |\omega|, \tag{8}$$

which implies that the rotors' magnetic moment must satisfy $m_{\mathrm{eff}} \geq \zeta|\omega|/B$. The constant phase difference in the locked state is then given by $\arcsin(\omega/K)$. Equally, one may view $\theta(t)$ as the position of a particle in a washboard potential

$$V(\theta, t) = -\,\omega\, \theta - \, K \cos\theta, \tag{9}$$

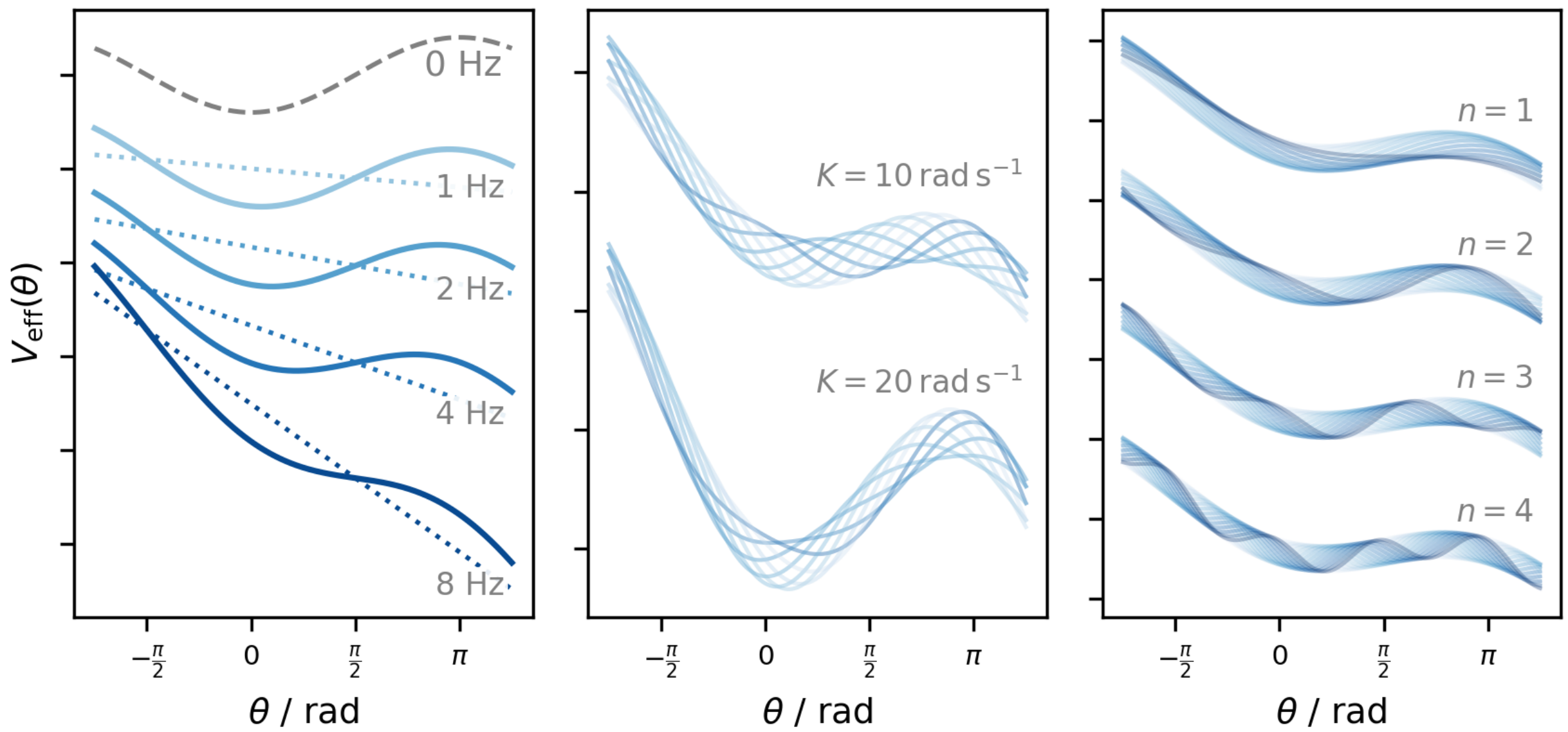

FIG. S11. **Effective potential $V_{\text{eff}}(\theta)$ and locking regimes under different conditions**. (a) Classic Adler potential in case of $\Delta E' = 0$: $V_{\text{eff}}(\theta) = -\omega\,\theta - K\cos\theta$ traps the phase in its cosine wells at low drive frequency f, whereas at high f the linear tilt $-\omega\theta$ dominates and phase slipping occurs. (b) The effect of a periodically explored surface-interaction $U(\varphi_r)$ leads to a time-dependent corrugation of $V_{\text{eff}}(\theta)$. Depending on the surface interaction strength, the coupling K might not be sufficient to keep the rotor phase-locked ($\Delta E = 4\,k_BT$, $n = 2$, $f = 1$ Hz). The weak coupling yields a shallow well that slips intermittently under the tilt. (c) The number of wells n in the surface interaction potential $U(\varphi_r)$ affects the corrugation of $V_{\text{eff}}(\theta)$ and allows fractional phase locking by providing additional stable wells ($\Delta E = 4\,k_BT$, $f = 1$ Hz, $K = 10\,\text{rad}\,\text{s}^{-1}$).

as illustrated in Fig.S11a. In case of sufficient coupling, the particle remains trapped in a local minimum despite the overall tilt – this corresponds to a constant phase difference and the regime of phase locking. However, if $|\omega| > K$, the tilt overwhelms the wells and the particle runs downhill with an average drift rate

$$\left\langle \dot{\theta} \right\rangle \;=\; \text{sign}(\omega)\sqrt{\omega^2 - K^2}. \tag{10}$$

In the lab frame this means that the rotor can no longer keep up with the applied field and instead *slips* in phase, see Fig. S12.

Stochastic Adler equation

If we now allow noise to kick in, $D > 0$ but $\Delta E' = 0$, we observe features of a noisy synchronizer. Then, the Adler equation reads $\dot{\theta} = \omega - K\sin\theta + \sqrt{2D}\,\eta(t)$, where phase locking can be thought of as a diffusing virtual particle θ trapped in a potential well that represents the locked state, see Eqn. 9. However, random thermal kicks occasionally give the particle enough energy to hop over the barrier ΔV into the next well. In the Kramers picture, escape events scale with

$$k \;\propto\; \exp\!\left(-\frac{\Delta V}{D}\right). \tag{11}$$

If D is small, the escapes are rare, and we can observe long periods of phase-locking under conditions deterministic synchronization would occur. Exemplary trajectories are shown in Fig. S13

Breathing potential

If we additionally consider the orientation-dependent local interactions of the DNA origami rotor with the surface, $\theta(t)$ experiences an periodic forcing term $n\Delta E'\,\sin\!\big(n(\omega t - \theta)\big)$, which leads to a 'breathing' energy landscape in the rotating frame

$$V(\theta, t) = -\,\omega\,\theta - \;K\cos\theta - \;\Delta E'\;\cos\!\big(n(\,\omega t - \theta)\big)\,, \tag{12}$$

where the washboard potential is continuously modulated by the oscillatory component $\Delta E'$, see also Fig. S11b and c. Then $\theta(t)$ cannot settle into a static fixed point anymore, but lives on a periodic orbit

$$\theta(t + qT) \;=\; \theta(t) + 2\pi\, m, \tag{13}$$

with integer number m that closes only after q cycles of the drive, while in the lab frame the rotor angle advances by $\Delta\varphi_r = 2\pi\,(q - m)$. The rotation number

$$N_r \;=\; \frac{\Delta\varphi_r}{2\pi} \;=\; \frac{q-m}{q} \tag{14}$$

then describes the number of full rotor turns per drive cycle. Exemplary rotor trajectories are provided in Figure S14.

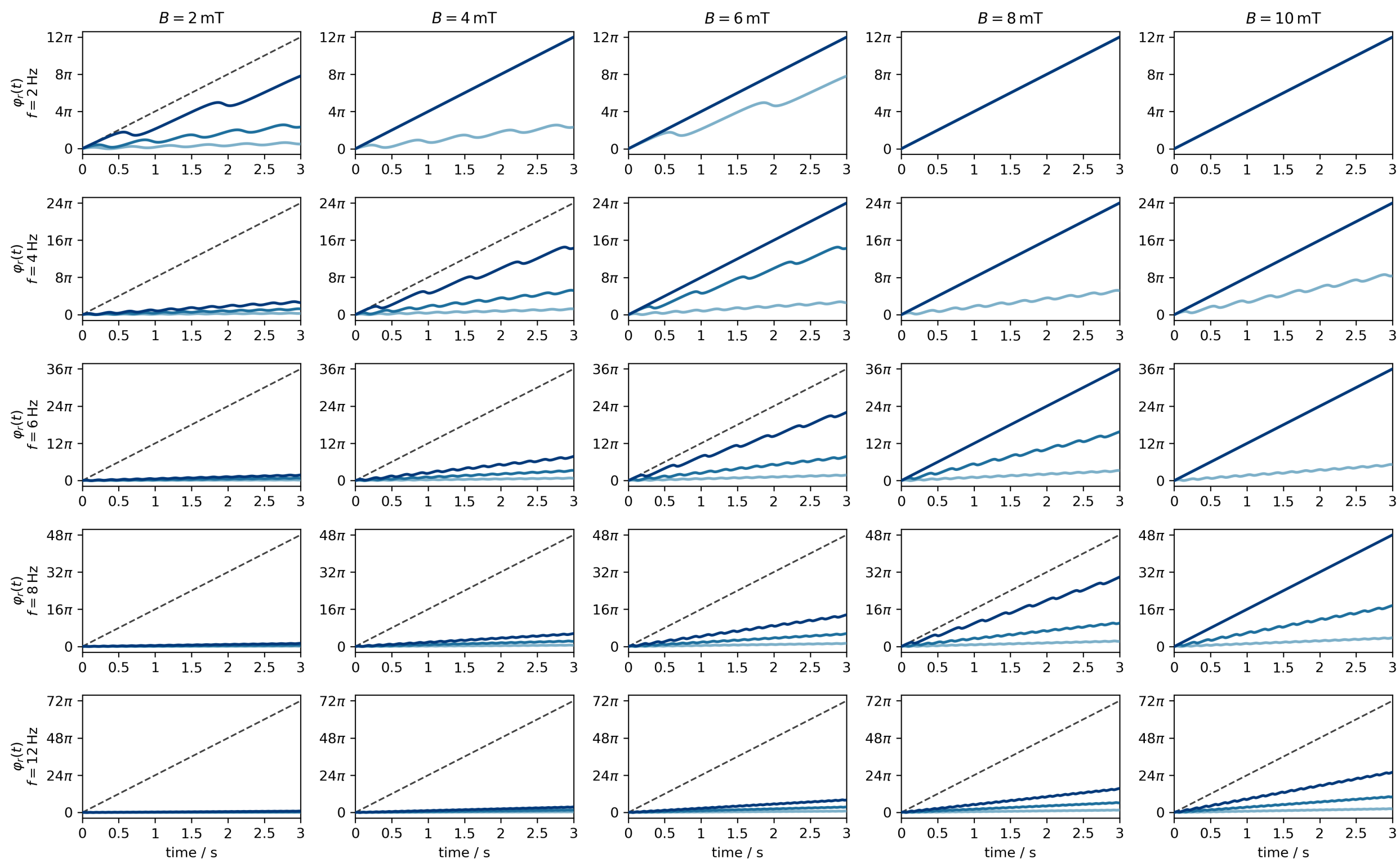

FIG. S12. **Deterministic rotor trajectories at different actuation frequencies and field strengths** for $D = 0$ and $\Delta E = 0$. Light to dark blue curves correspond to rotors with effective magnetic moments $m_{\mathrm{eff}} = \{1, 2, 3\} \cdot 10^{-18}\,\mathrm{A\,m^2}$. The trajectories that fall on the top of the external drive (dashed line) are fully synchronous. The ones that show oscilli-ations are driven with the field, yet at lower velocity and showing phase-slips (e.g. light blue trace at 6 mT and 2 Hz).

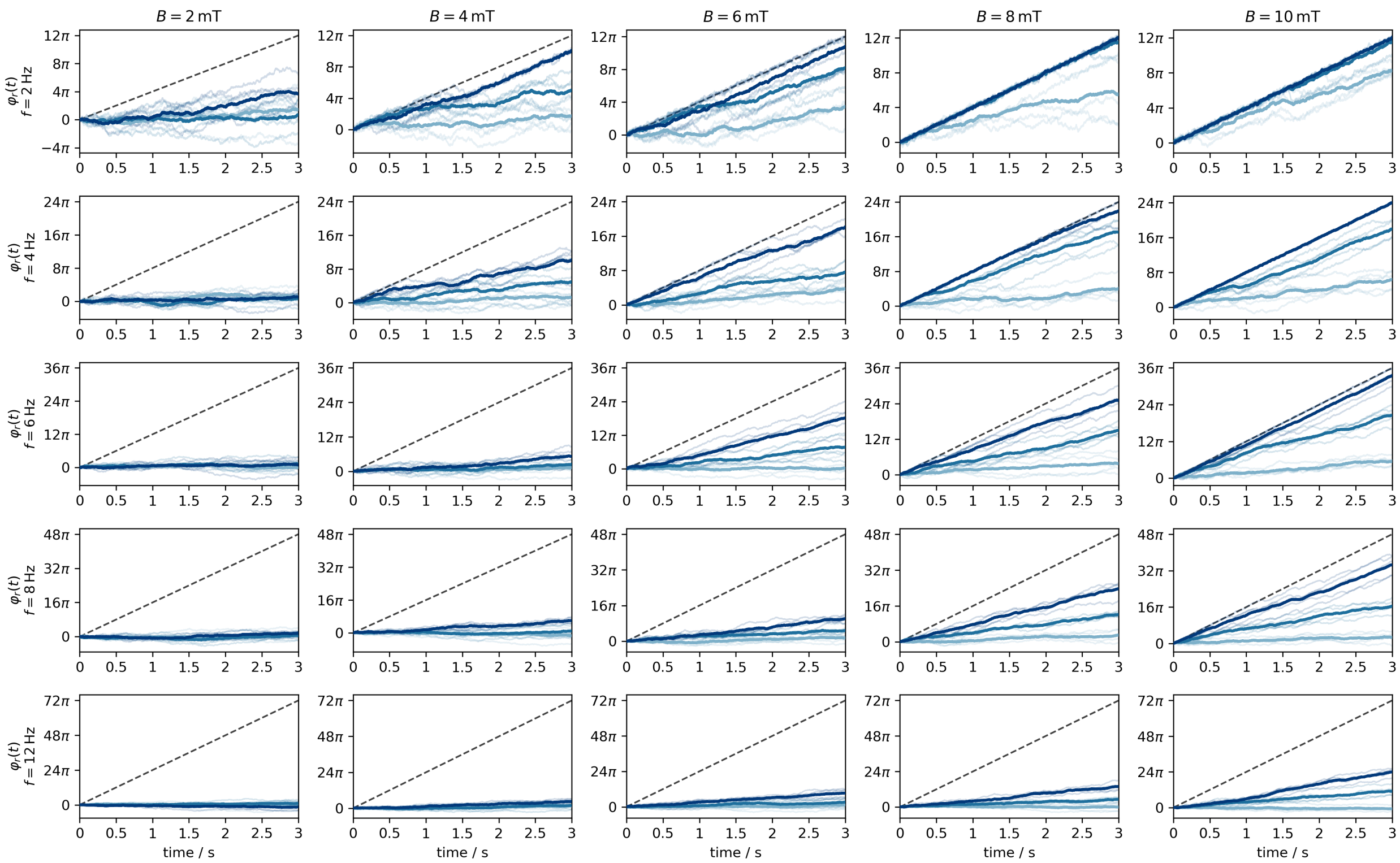

FIG. S13. **Stochastic rotor trajectories at different actuation frequencies and field strengths** for rotors with a rotational diffusion coefficient $D = 8\,\mathrm{rad}^2\,\mathrm{s}^{-1}$. Light to dark blue curves correspond to rotors with effective magnetic moments $m_{\mathrm{eff}} = \{1, 2, 3\} \cdot 10^{-18}\mathrm{A\,m}^2$. For each magnetic moment 5 exemplary trajectories and their mean trend are shown.

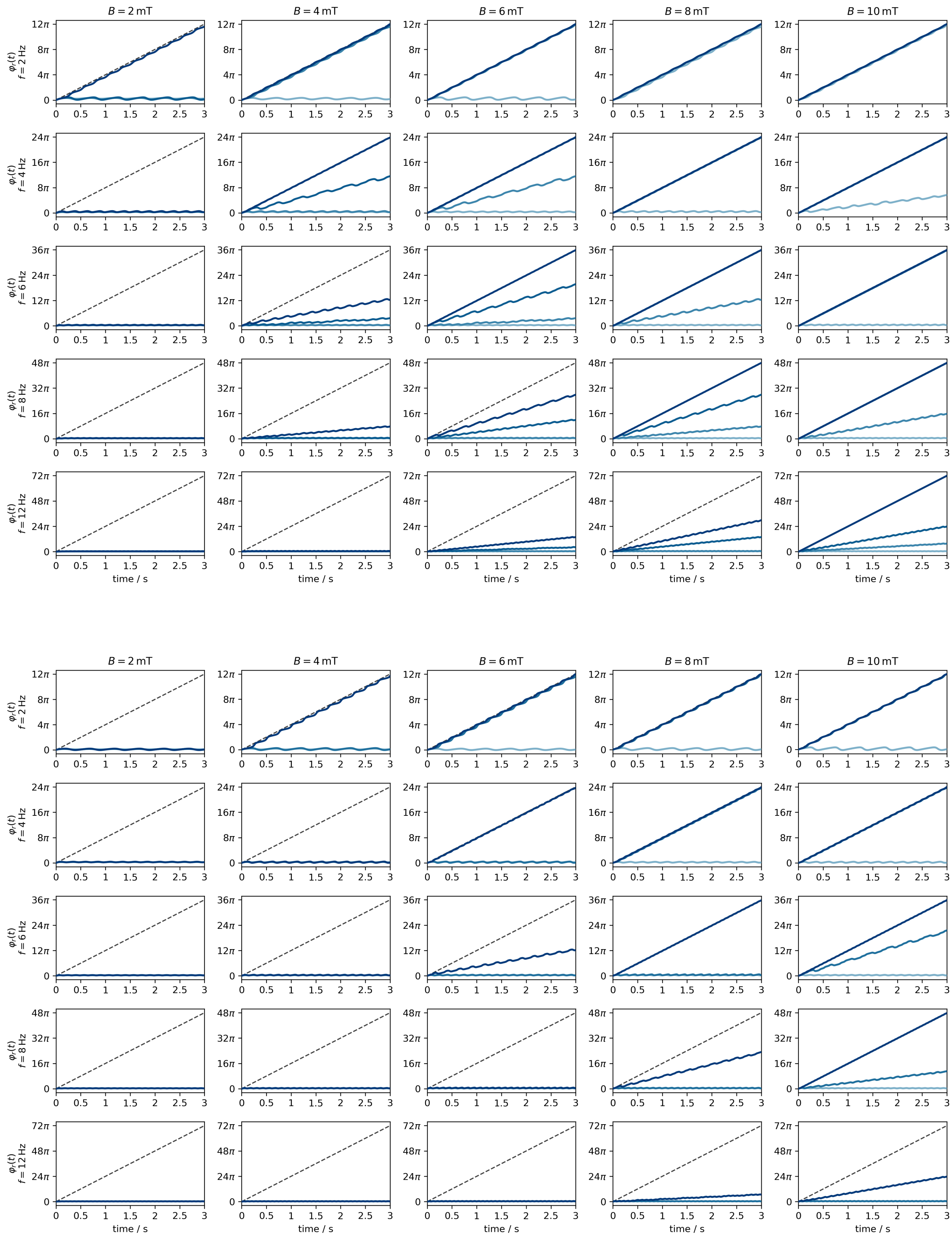

FIG. S14. **Deterministic rotor trajectories at different actuation frequencies and field strengths for rotors with an intrinsic sinusoidal π-periodic energy landscape** with $\Delta E = 0.5 k_B T$ (top) and $1 k_B T$ (bottom). Light to dark blue curves correspond to rotors with effective magnetic moments $m_{\text{eff}} = \{1, 2, 3\} \cdot 10^{-18} \mathrm{A\,m^2}$.

8. Intrinsic energy landscapes of 4-MNC MADONAs

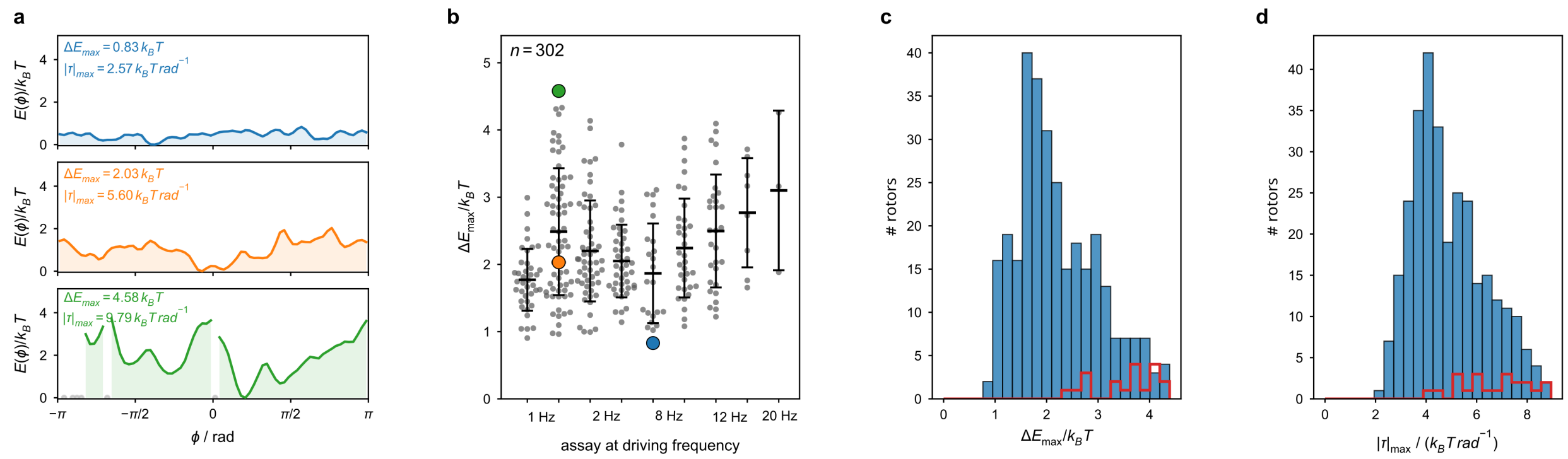

FIG. S15. **Intrinsic energy landscapes arising from rotor–surface interactions.** (a) Three representative rotor energy landscapes obtained from Boltzmann inversion of the angular probability distribution, $E(\varphi) = -k_BT \ln p(\phi)$. (b) Distribution of energy barrier heights across all rotor ensembles included in the drop-out analysis. (c) Barrier heights of all rotors ($n = 302$) that sampled at least 85% of the angular range (blue) compared with rotors that remained trapped in local energy wells (red). (d) Corresponding maximum restoring torques required to escape the local energy wells.

9. Rotor trajectories used in TIRFM-RMF assays

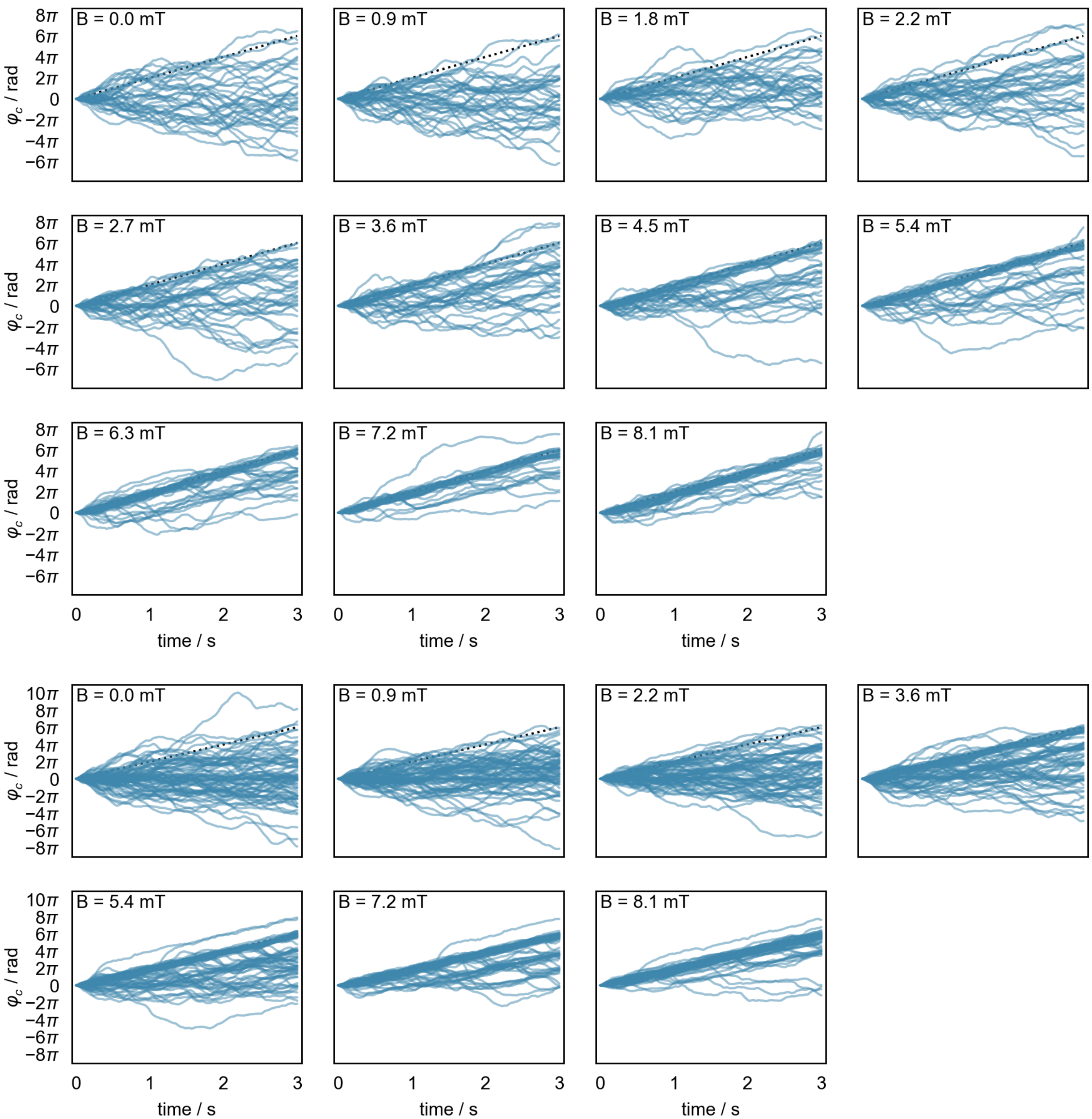

FIG. S16. TIRFM-RMF assay at $1\,\mathrm{Hz}$ corresponding to data shown in Figure 3i–k. Smoothed rotor trajectories for rotations at $1\,\mathrm{Hz}$ with different field strengths for two different set of rotors ($n = 39$ and $n = 76$). The dashed line corresponds to a perfect synchronization with the field.

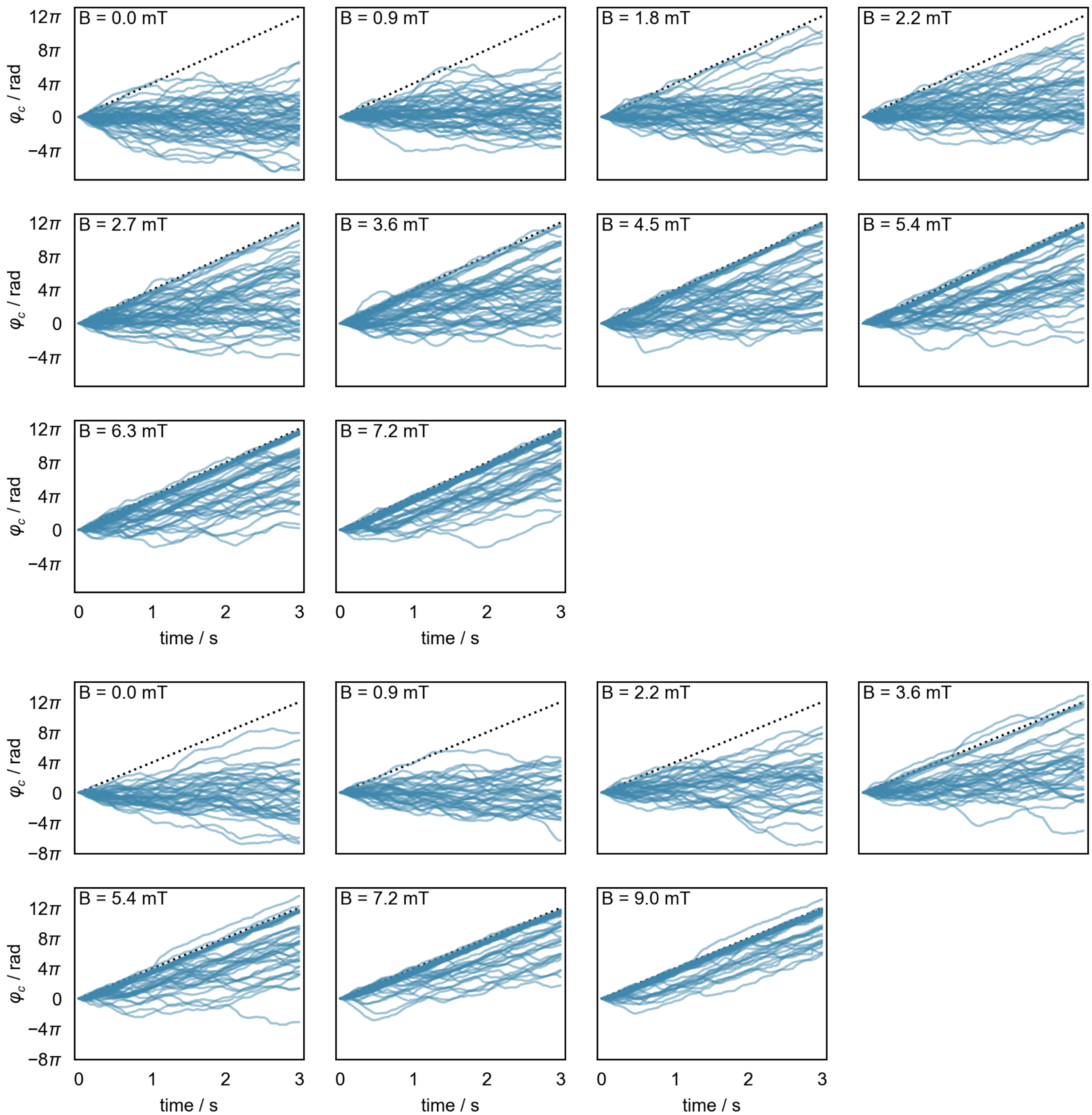

FIG. S17. TIRFM-RMF assay at $2\,\mathrm{Hz}$ corresponding to data shown in Figure 3g and Figure 3i–k. Smoothed rotor trajectories for rotations at $2\,\mathrm{Hz}$ with different field strengths for two different set of rotors ($n = 54$ and $n = 43$).

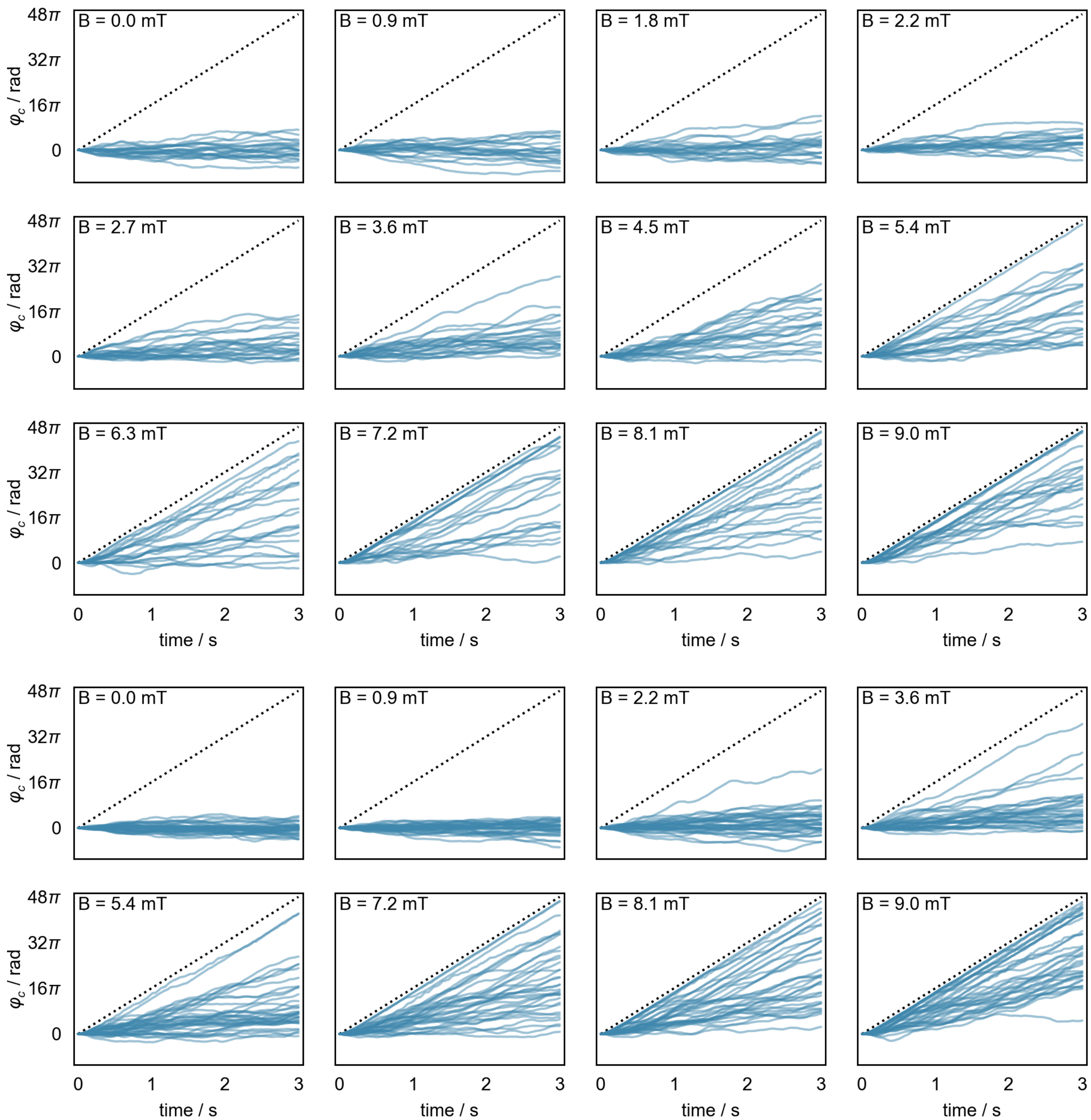

FIG. S18. TIRFM-RMF assay at $8\,\mathrm{Hz}$ corresponding to data shown in Figure 3h–k. Smoothed rotor trajectories for rotations at $8\,\mathrm{Hz}$ with different field strengths for two different set of rotors ($n = 23$ and $n = 36$).

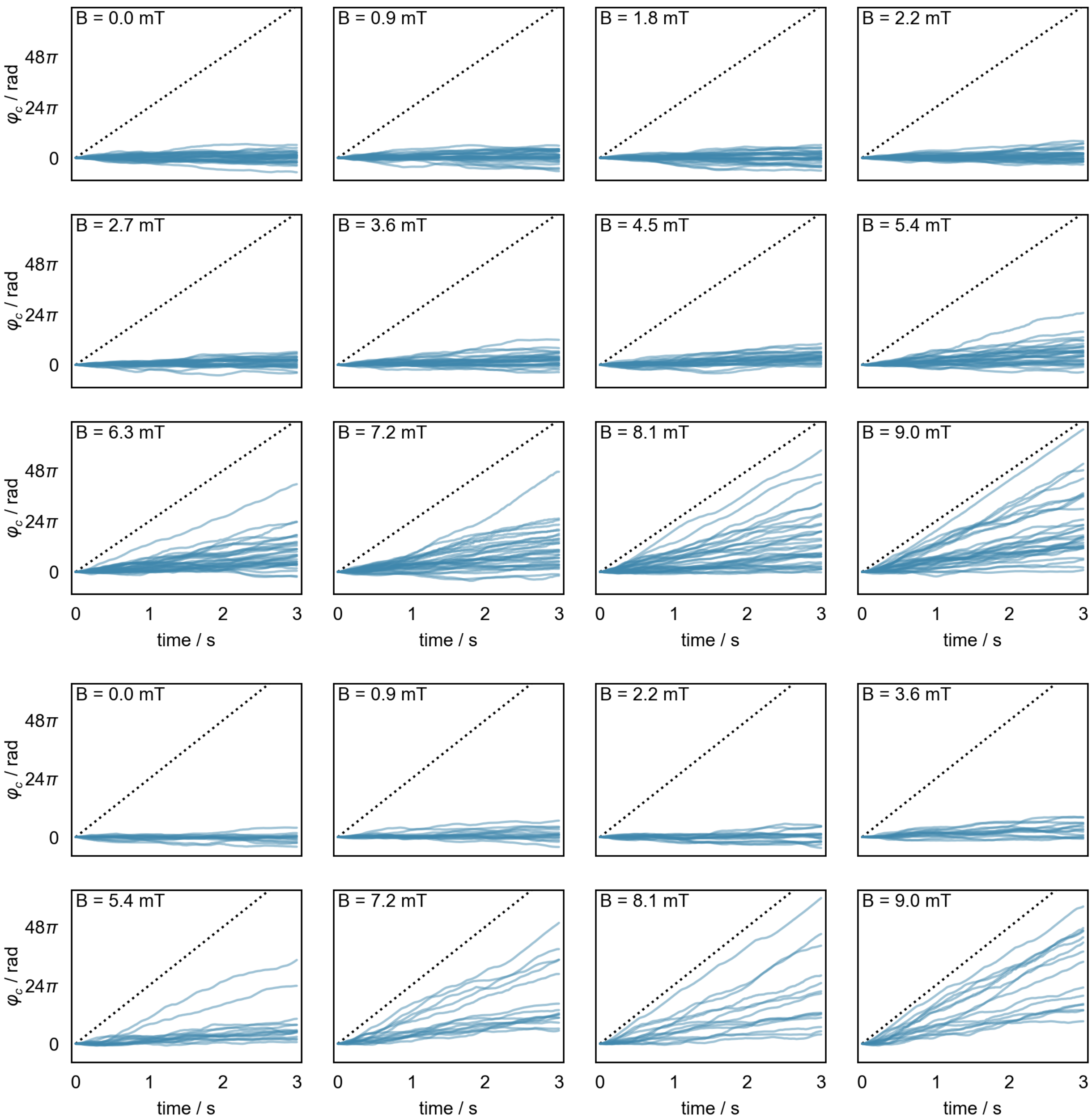

FIG. S19. TIRFM-RMF assay at $12\,\mathrm{Hz}$ corresponding to data shown in Figure 3i–k. Smoothed rotor trajectories for rotations at $12\,\mathrm{Hz}$ with different field strengths for two different set of rotors ($n = 32$ and $n = 15$).

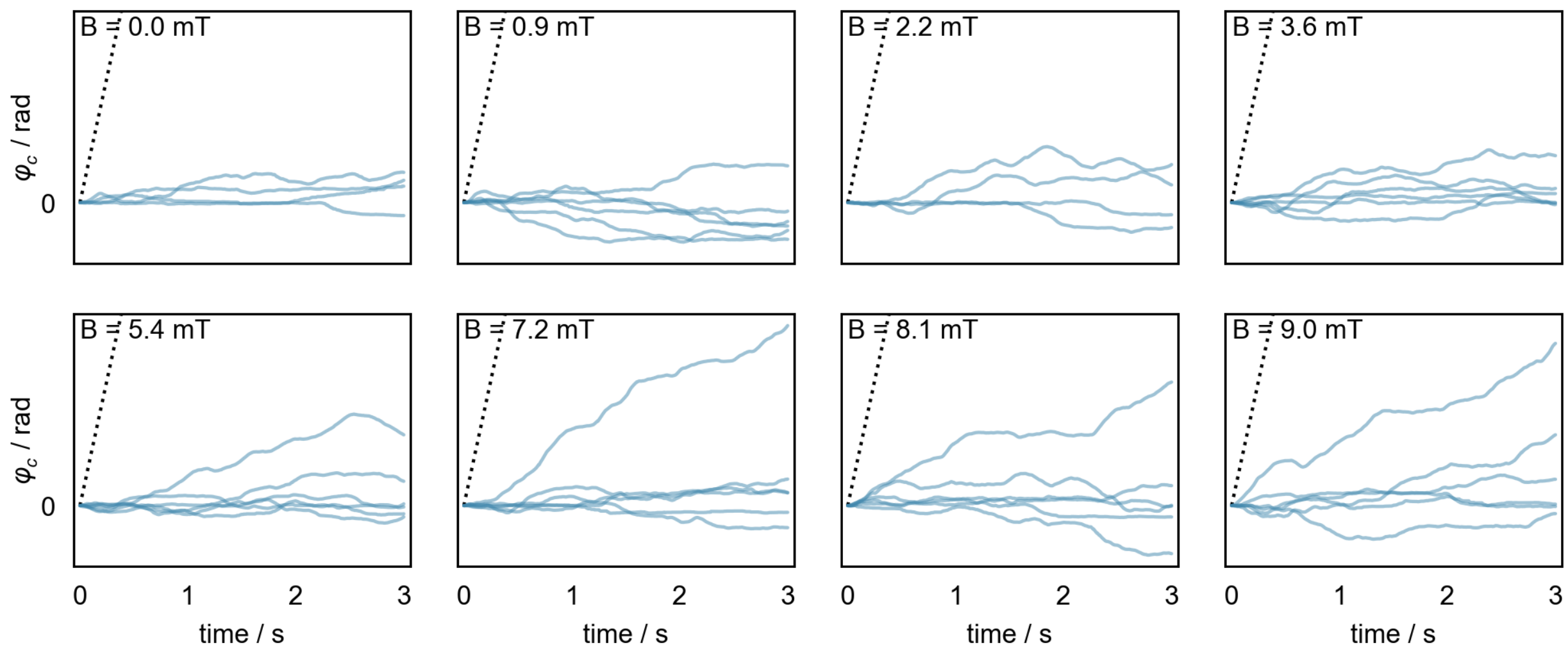

FIG. S20. TIRFM-RMF assay at $20\,\mathrm{Hz}$ corresponding to data shown in Figure 3i–k. Smoothed rotor trajectories for rotations at $20\,\mathrm{Hz}$ with different field strengths for one set of rotors ($n = 6$).

10. Magnetic torques of 4-MNC MADONAs from frequency-resolved drop-out analyses

To quantify the magnetic torques of 4-MNC MADONAs from the rotation assays shown in Figure 3e and Figure 3f, we calculated the phase-locking value (PLV) with respect to an externally rotating magnetic field of angular frequency ω

$$\mathrm{PLV} = \left|E\left[e^{i\theta(t)}\right]\right| = \left|\frac{1}{N}\sum_{n=1}^{N} e^{i(\omega t_n - \varphi_{r,n})}\right|,$$

where $\theta(t) = \omega t - \varphi_r(t)$ is the instantaneous phase offset between the external field and the rotor angle $\varphi_r(t)$, and $E[\cdot]$ denotes the average complex pointer on the unit circle. For a rotor that is phase-locked to the external field, the phase offset remains constant over time. In this case, the complex pointer $e^{i\theta(t)}$ points in a fixed direction on the unit circle, and the resulting average vector has unit length. By contrast, if the rotor does not follow the field, the phase offset varies over time, the complex pointers point in different directions, and their average vector has a length smaller than one. Thus, $\mathrm{PLV} = 1$ indicates perfect phase locking and co-rotation with the external field, whereas $\mathrm{PLV} = 0$ indicates the absence of phase synchrony between rotor and drive.

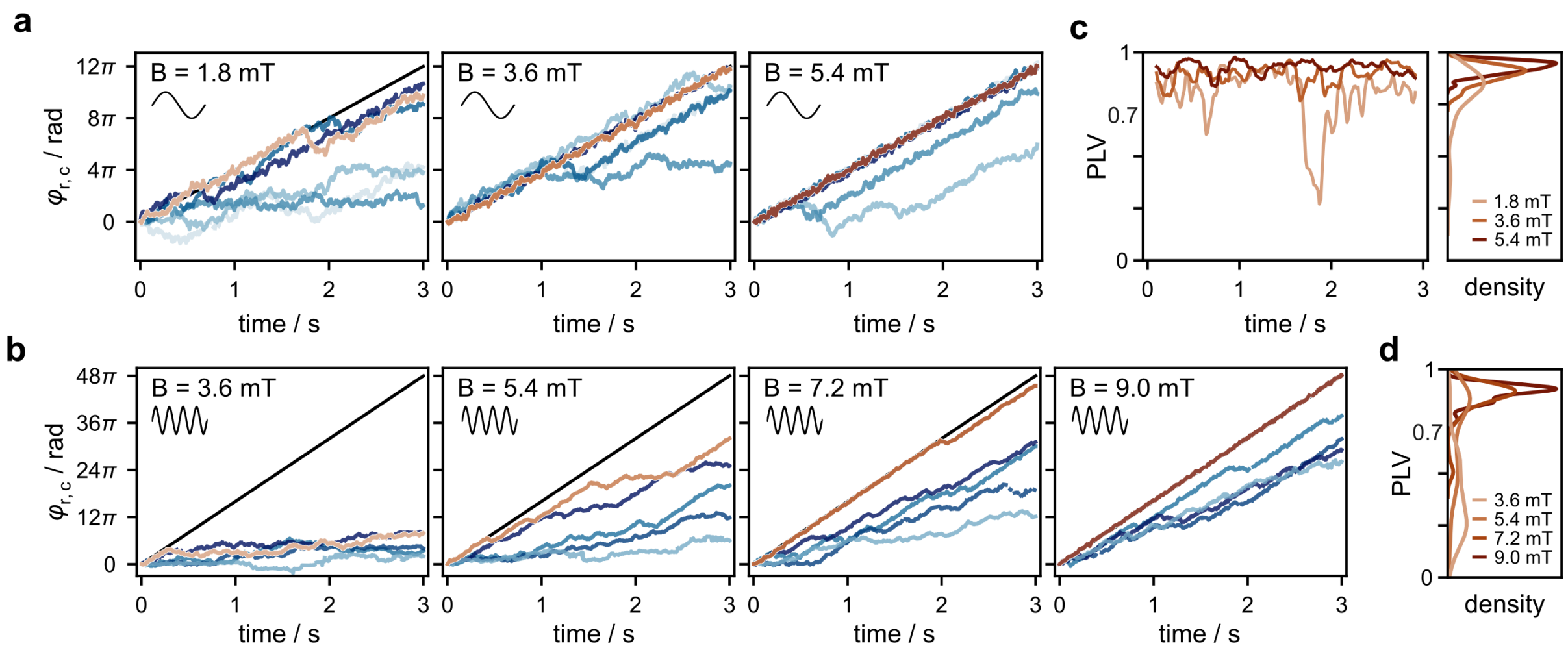

FIG. S21. **Rotor trajectories from Figure 3 and corresponding phase-locking values.** (a,b) Raw angular trajectories of rotors subjected to rotating magnetic fields at (a) 2 Hz and (b) 8 Hz, respectively. The red trajectory highlights the representative rotor used for the PLV analysis. (c) Windowed phase-locking values, $\mathrm{PLV}(t)$, for the highlighted rotor in (a), together with the corresponding PLV histograms. (d) PLV histograms for the highlighted rotor in (b) during actuation at 8 Hz.

Figure S21a,b show the rotor trajectories from Figure 3 together with the temporal evolution and distribution of the PLV for the highlighted rotor in Figure S21c,d. The PLV was estimated from overlapping 200 ms time windows during actuation at 2 Hz and 8 Hz, respectively. At low magnetic field strengths, thermal fluctuations substantially perturb the rotor motion and broaden the phase-offset distribution, resulting in reduced phase locking. In addition, transient phase slips can strongly decrease the PLV even when the rotor is still visibly driven by the rotating field, as illustrated by the highlighted trajectory at $B = 1.8$ mT. The PLV therefore provides a stringent measure of phase locking, with high PLV indicating clear synchronization between rotor and external field. However, in the presence of thermal noise, no general PLV threshold can reliably distinguish purely diffusive motion from field-driven but imperfectly phase-locked motion.

We further note that PLV measurements are not directly comparable across different actuation frequencies when the total measurement duration is kept fixed. For a constant rotational diffusion coefficient D, higher actuation frequencies produce more drive cycles within the same observation time and the deterministic angular advance imposed by the field becomes larger relative to the diffusive angular fluctuations, increasing the effective

signal-to-noise ratio for detecting coherent phase locking. Consequently, phase locking can be identified more robustly at higher actuation frequencies of ≥ 2 Hz. Conversely, purely diffusive rotor trajectories can still yield nonzero PLVs when evaluated against a virtual reference drive, particularly at low reference frequencies, where diffusive angular displacements occur on a comparable timescale as the imposed drive period (see Figure S23). We therefore performed TIRFM–RMF assays for each field of view at a fixed actuation frequency while varying the magnetic field strength, using the resulting PLV distributions during pure diffusion to determine the breakdown points of the rotor ensemble.

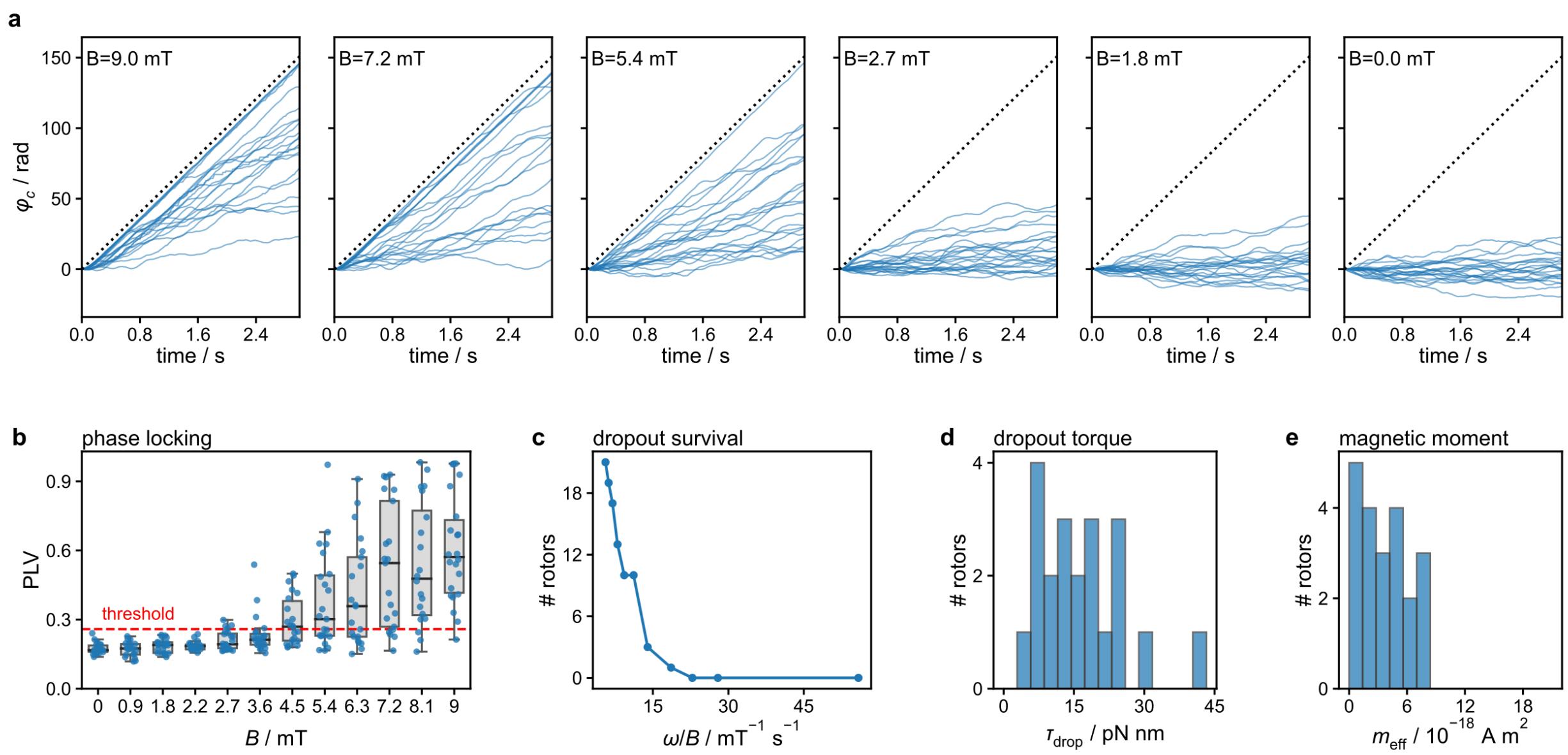

FIG. S22. **Exemplary drop-out assay analysis for 4-MNC MADONAS in a rotating magnetic field at 8 Hz.** (a) Smoothed angular trajectories of individual rotors during rotating-field actuation at different magnetic field amplitudes. The dotted black lines indicate ideal phase-locked rotation at the imposed driving frequency. (b) Phase-locking values (PLVs) of rotors from a single field of view as a function of magnetic field amplitude. The PLV measured under diffusive, non-driven conditions was used to define an ensemble-specific threshold, $> \mathrm{PLV}_{\mathrm{diff}}$, separating driven rotors from unresponsive rotors. A rotor was classified as dropped out when its PLV fell below this threshold upon decreasing the field amplitude. (c) Dropout survival curve showing the number of rotors remaining phase locked as a function of the normalized driving strength, ω/B. (d) Magnetic torque at dropout, estimated from torque balance via $\tau_{\mathrm{visc}} = \omega\zeta$, where ζ is the rotational drag coefficient obtained from diffusive trajectories. (e) Distribution of effective magnetic moments estimated for individual rotors as $m_{\mathrm{eff}} = \omega\zeta/B_{\mathrm{drop}}$.

As exemplarily illustrated in Figure S22a,b, the fraction of driven rotors increases markedly with magnetic field strength (see also Figure 3g,h), consistent with stronger magnetic torque stabilizing phase locking. However, rotor drop-out is not governed solely by the classical torque balance between magnetic torque and viscous drag. Local restoring torques arising from the individual rotor energy landscapes can interfere with driven rotation and transiently trap the rotor in preferred angular states [8, 9], as illustrated in Figure S14. Thus, a loss of co-rotation with the external drive can arise from several experimentally inseparable contributions. First, the magnetic torque exerted by the MNCs may be insufficient to overcome the viscous drag torque, corresponding to classical drop-out (Figure S12). Second, thermal fluctuations may induce stochastic phase slips, even when the rotor remains coupled to the drive (Figure S13). Third, rotor–surface interactions may introduce additional local restoring torques that cannot be overcome by the applied magnetic torque. Nevertheless, as shown in Figure S22a, driven rotor trajectories remain clearly distinguishable from freely diffusive motion.

We therefore established a non-strict, operational classification based on the PLV. A rotor was considered to at least transiently follow the external field synchronously if its mean PLV exceeded $\mathrm{PLV}_{\mathrm{diff,max}}$, where $\mathrm{PLV}_{\mathrm{diff,max}}$ denotes the maximum PLV observed for the freely diffusing ensemble under the corresponding virtual reference drive. Using this PLV threshold, we performed drop-out analyses on the datasets (shown in Figure S22 for an exemplary field of view, see also Figure 3g,h) to estimate the effective magnetic moment, m_{eff}, of individual rotors. Survival curves were obtained by counting the number of rotors that satisfied the PLV threshold as a function

of the normalized field parameter ω/B (Figure S22c). Upon decreasing the magnetic field strength at fixed ω, more and more rotors drop out. For each rotor, the corresponding drop-out condition provides a lower-bound estimate of m_{eff}. Because the TIRFM–RMF assays were performed at different driving frequencies, the rotors were challenged with different viscous drag torques at a given magnetic field strength. Figure 3i summarizes the overlaid drop-out curves from all assays and the corresponding estimates of the effective magnetic moments. The resulting distribution (Figure 3j) spans from approximately $1 \times 10^{-18}\ \text{Am}^2$, comparable to the magnetic moment expected for a single MNC, to values more than one order of magnitude larger. Consequently, the magnetic torque generated by 4-MNC MADONAs ranges from a few $\text{pN}\,\text{nm}$ to more than $100\ \text{pN}\,\text{nm}$ (Figure 3k).

A closer inspection of the magnetic moment and torque distributions reveals a subset of rotors with torques on the order of 50–$100\ \text{pN nm}$ (Figure 3k). We attribute these high-torque rotors to cases in which individual binding sites were occupied not only by monomeric MNCs, but by MNC dimers or trimers. Such clusters could form during polymer coating and, if their magnetic moments are aligned, would contribute disproportionately to the total effective magnetic moment of the rotor. Conversely, the subset of rotors with torques below $10\ \text{pN}, \text{nm}$ likely corresponds to rotors carrying fewer than four MNCs, rotors in which the magnetic moments of attached MNCs are partially opposed, or rotors in which one or more MNC moments have a substantial out-of-plane component and therefore contribute less to the in-plane torque exerted by the lateral rotating magnetic field.

MADONAs without MNCs attached exposed to a rotating magnetic field

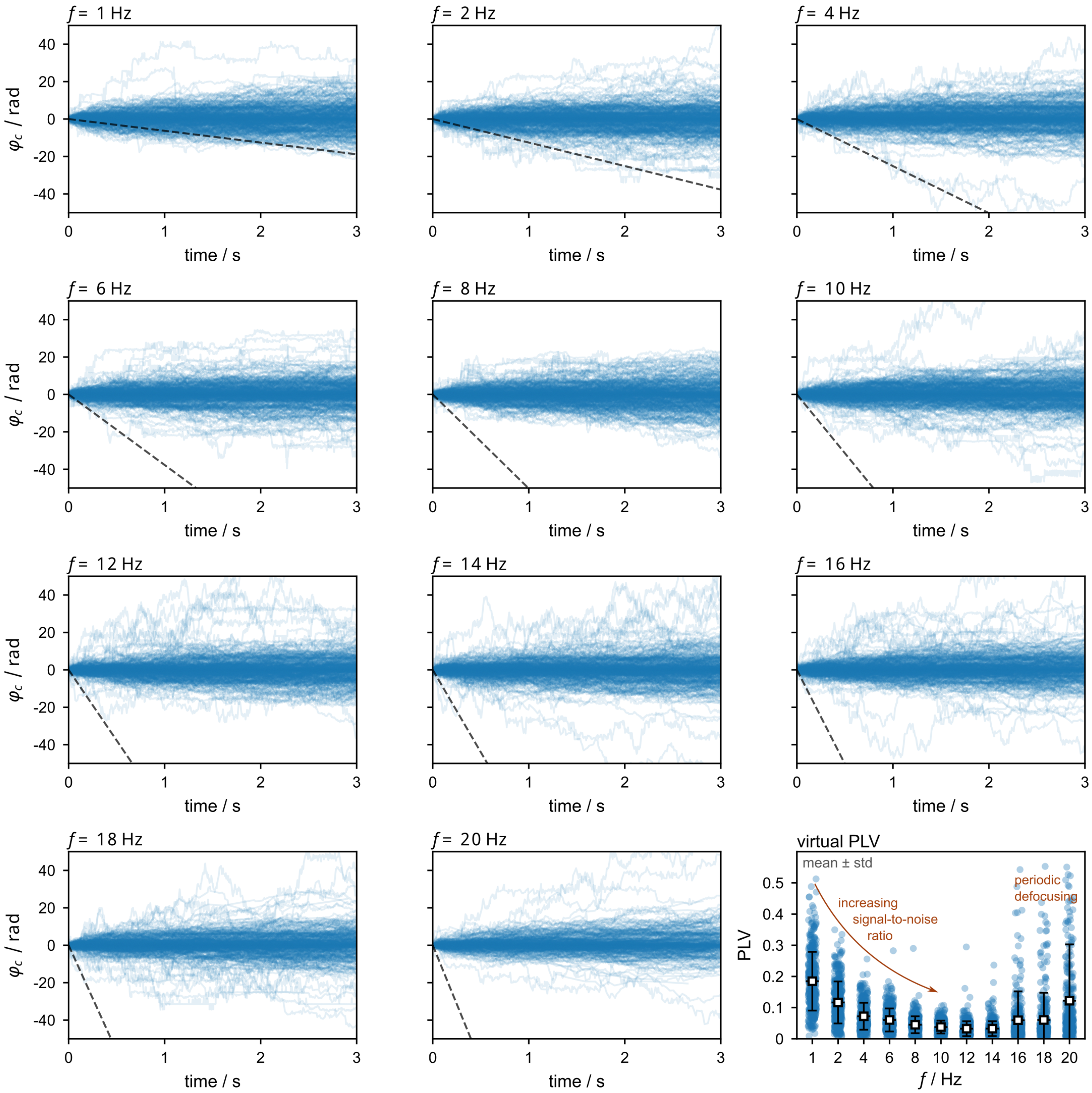

FIG. S23. **Rotors without MNCs in a rotating magnetic field at different frequencies**. Trajectories of unfiltered rotor ensembles exposed to a rotating magnetic field with 4.5 mT field strength at varying driving frequencies. We computed individual PLVs based on the rotor trajectories and a virtual drive (dashed line). As the advancing angle at higher frequencies leads to sustantially higher signal-to-noise ratios, we observe decreasing PLV thresholds. At high frequencies and strong fields, thermal drift induced by heating coils can induce jitter of the focal plane and increase diffusion.

11. Trap stiffness data for 4-MNC MADONAs

Exemplary 4-MNC MADONAs

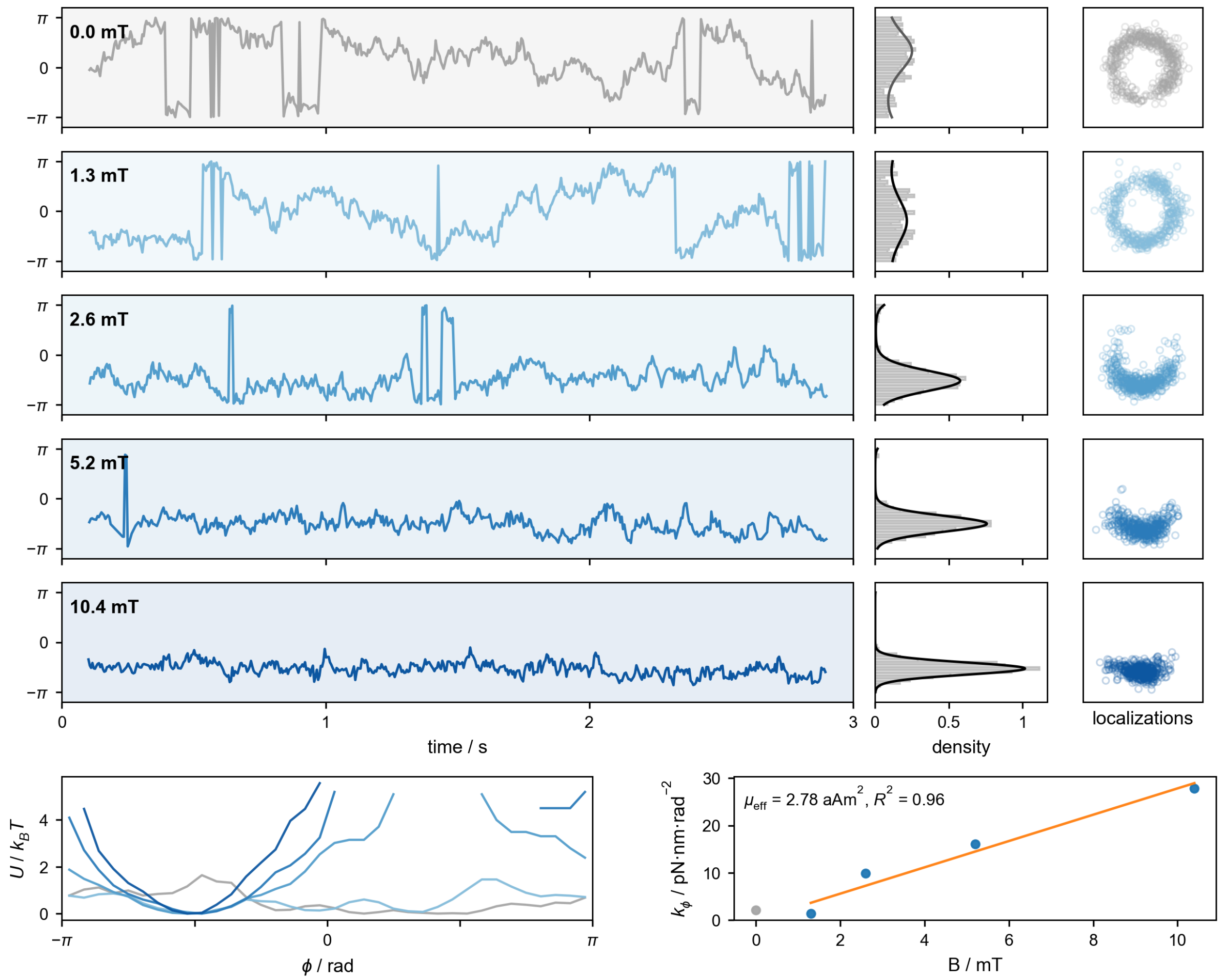

FIG. S24. **Exemplary 4-MNC MADONA rotor during clamping experiment**. Trajectories during clamping, angular localization histogram and van Mises fit, scatter plot of x, y- localizations. Energy landscapes (bottom left) and traps stiffness as function of the applied field (bottom right).

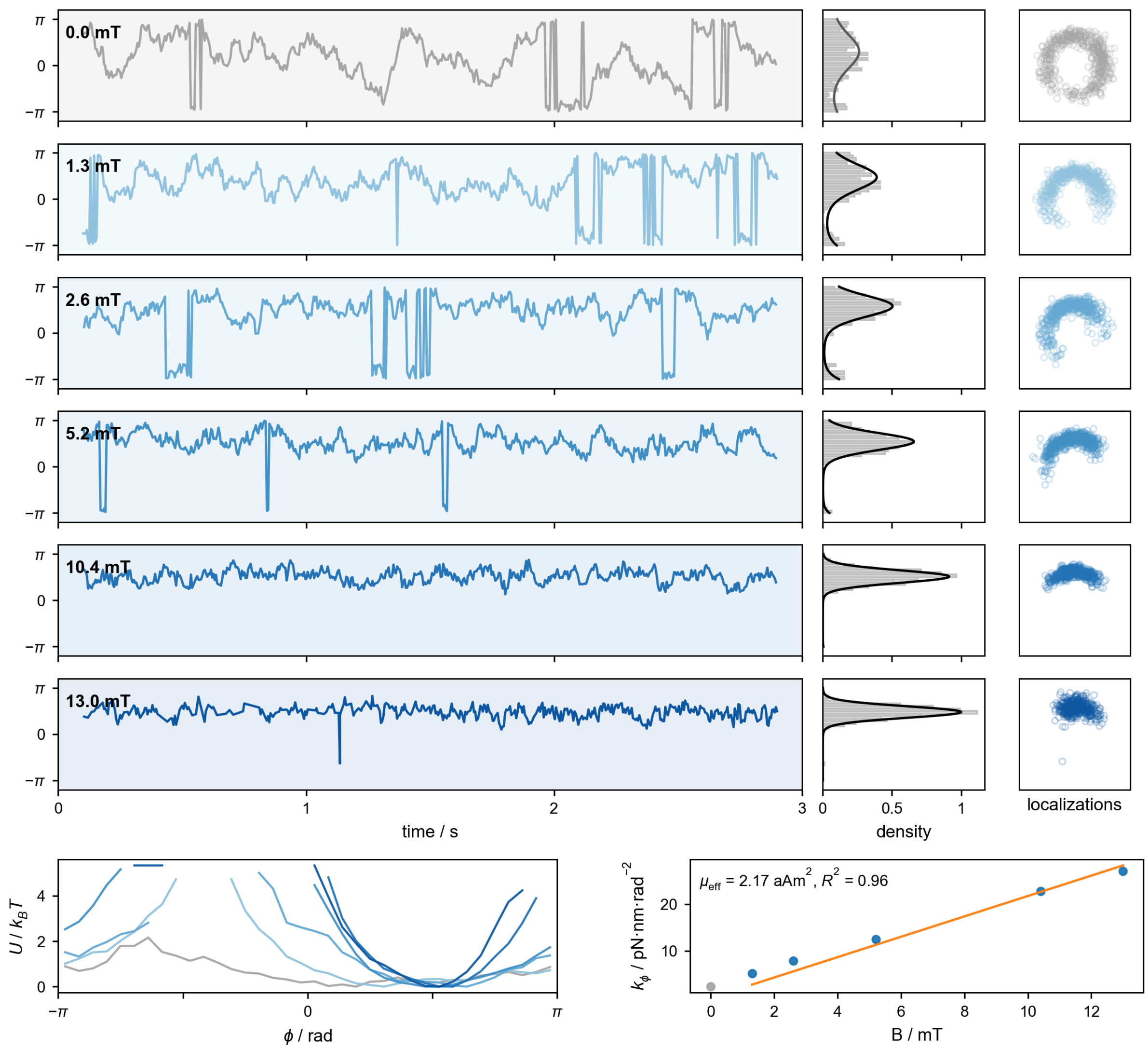

FIG. S25. **Cont. Exemplary 4-MNC MADONA rotor during clamping experiment**.

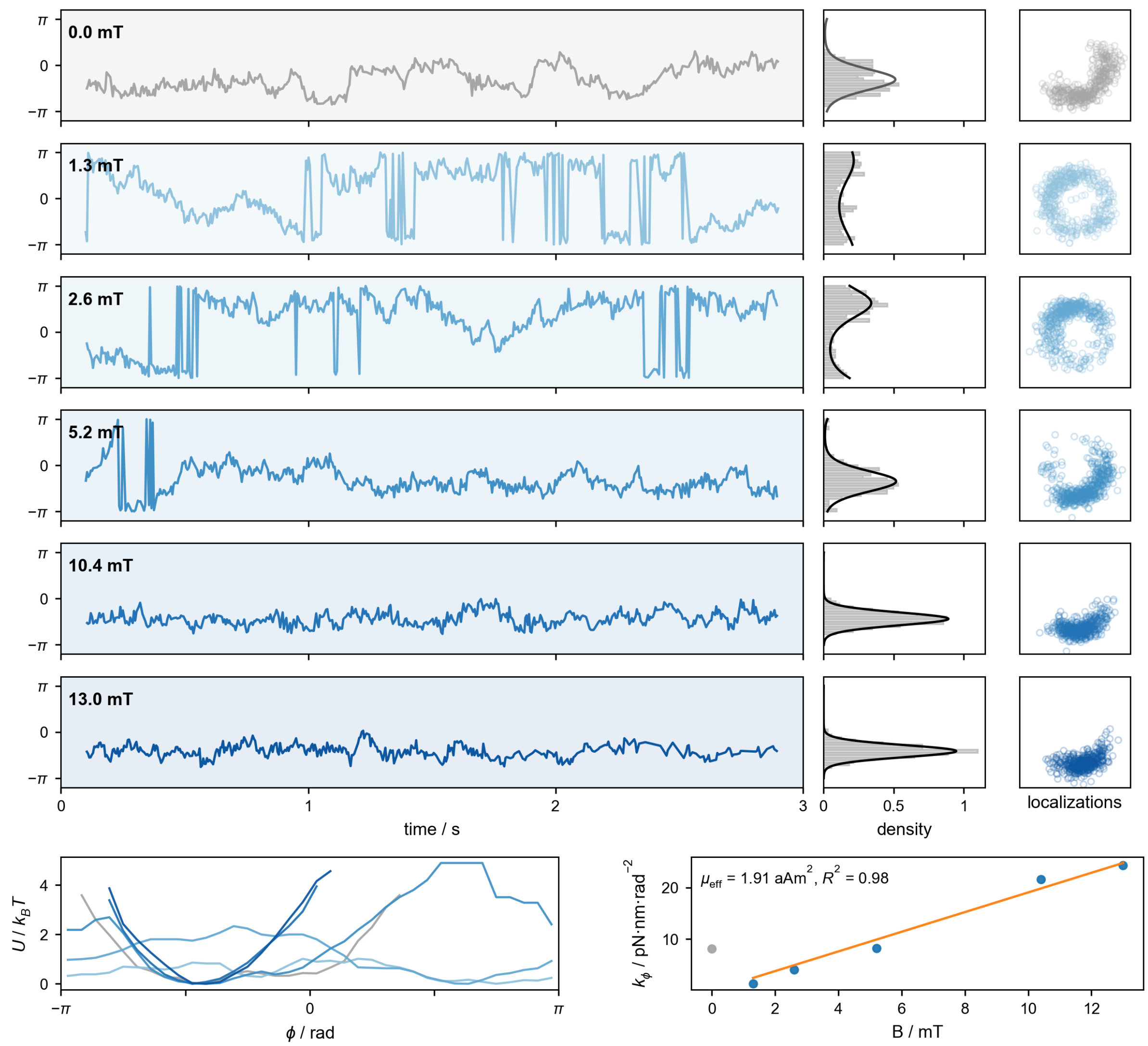

FIG. S26. **Cont. Exemplary 4-MNC MADONA rotor during clamping experiment**.

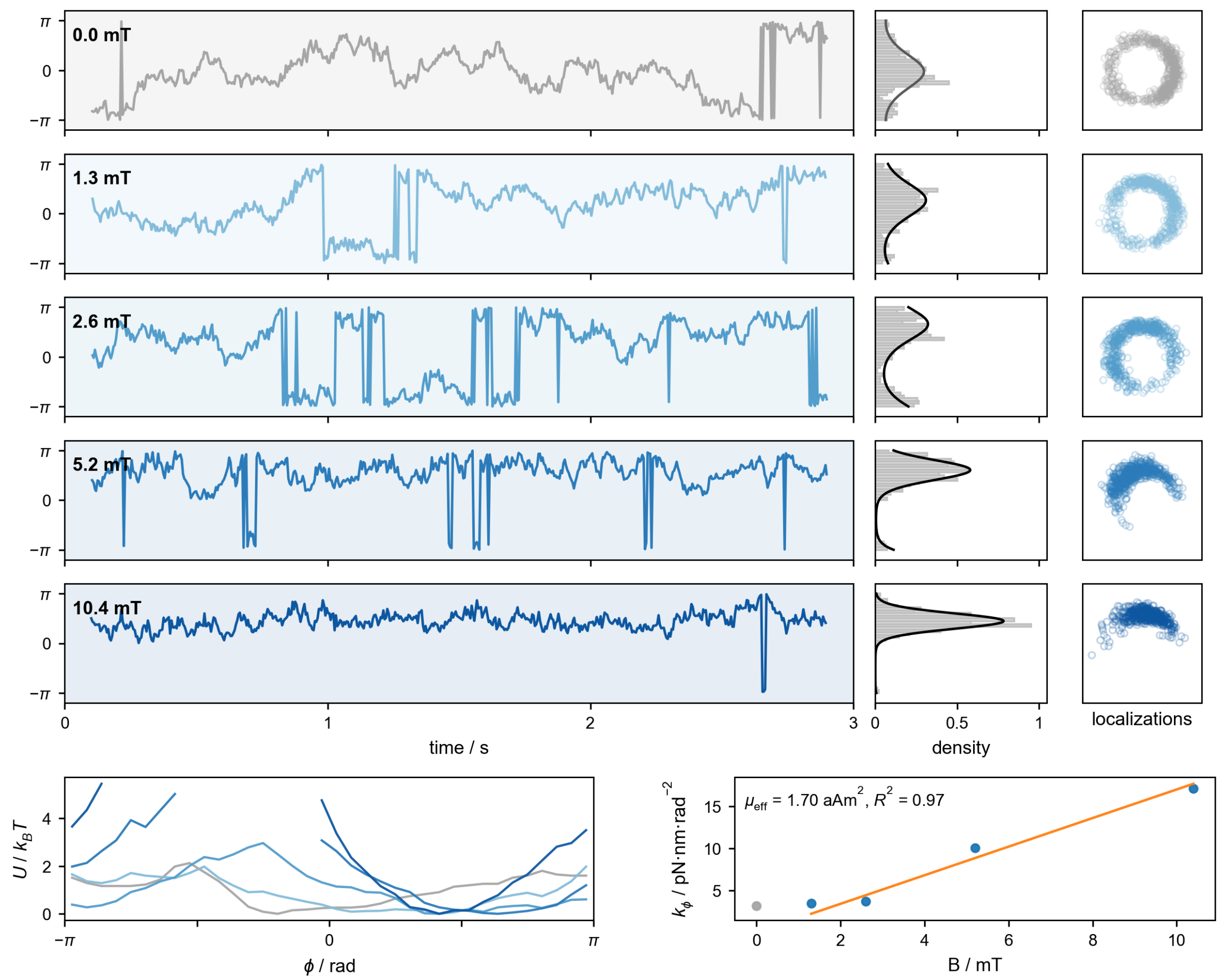

FIG. S27. **Cont. Exemplary 4-MNC MADONA rotor during clamping experiment**.

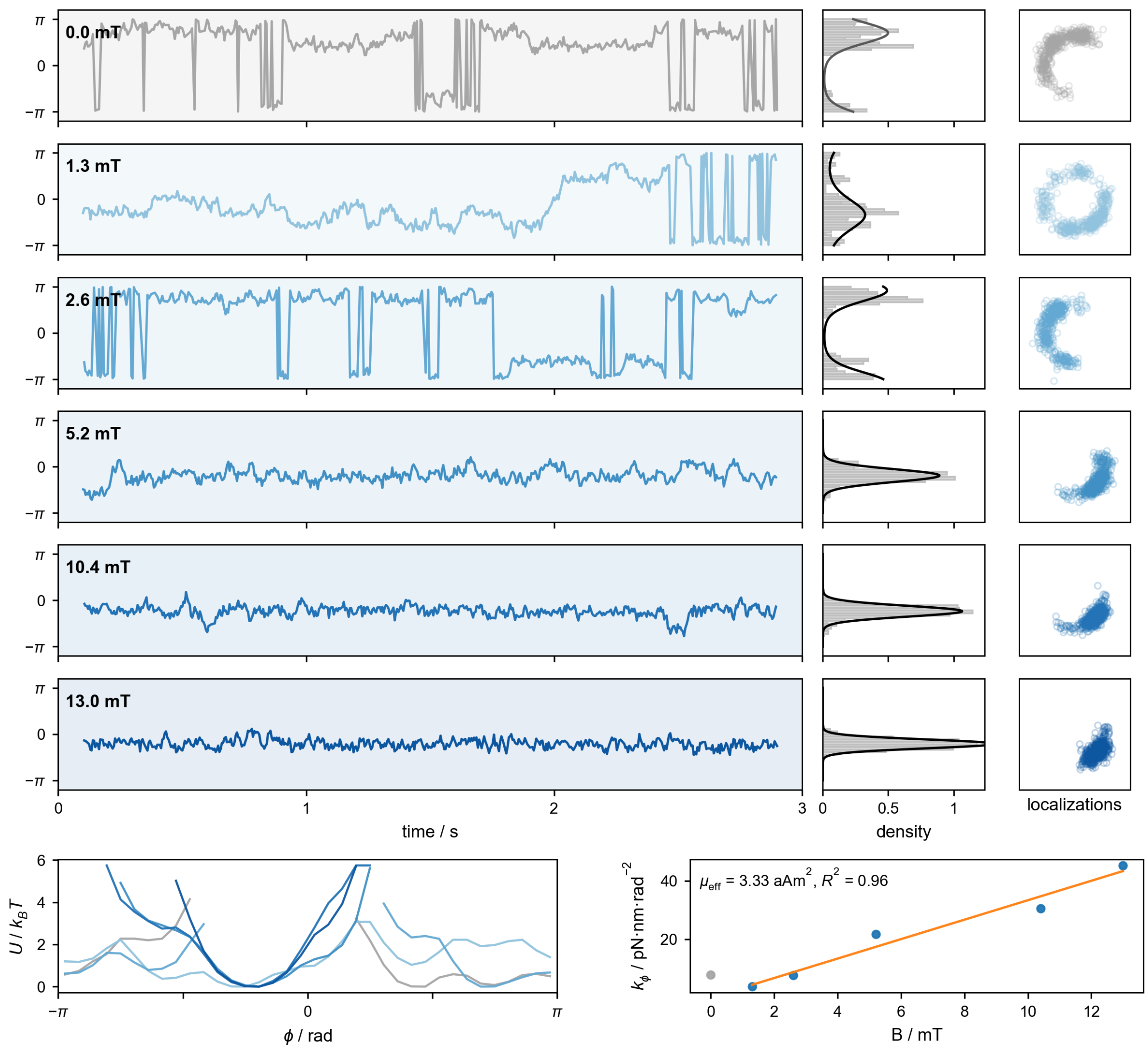

FIG. S28. **Cont. Exemplary 4-MNC MADONA rotor during clamping experiment**.

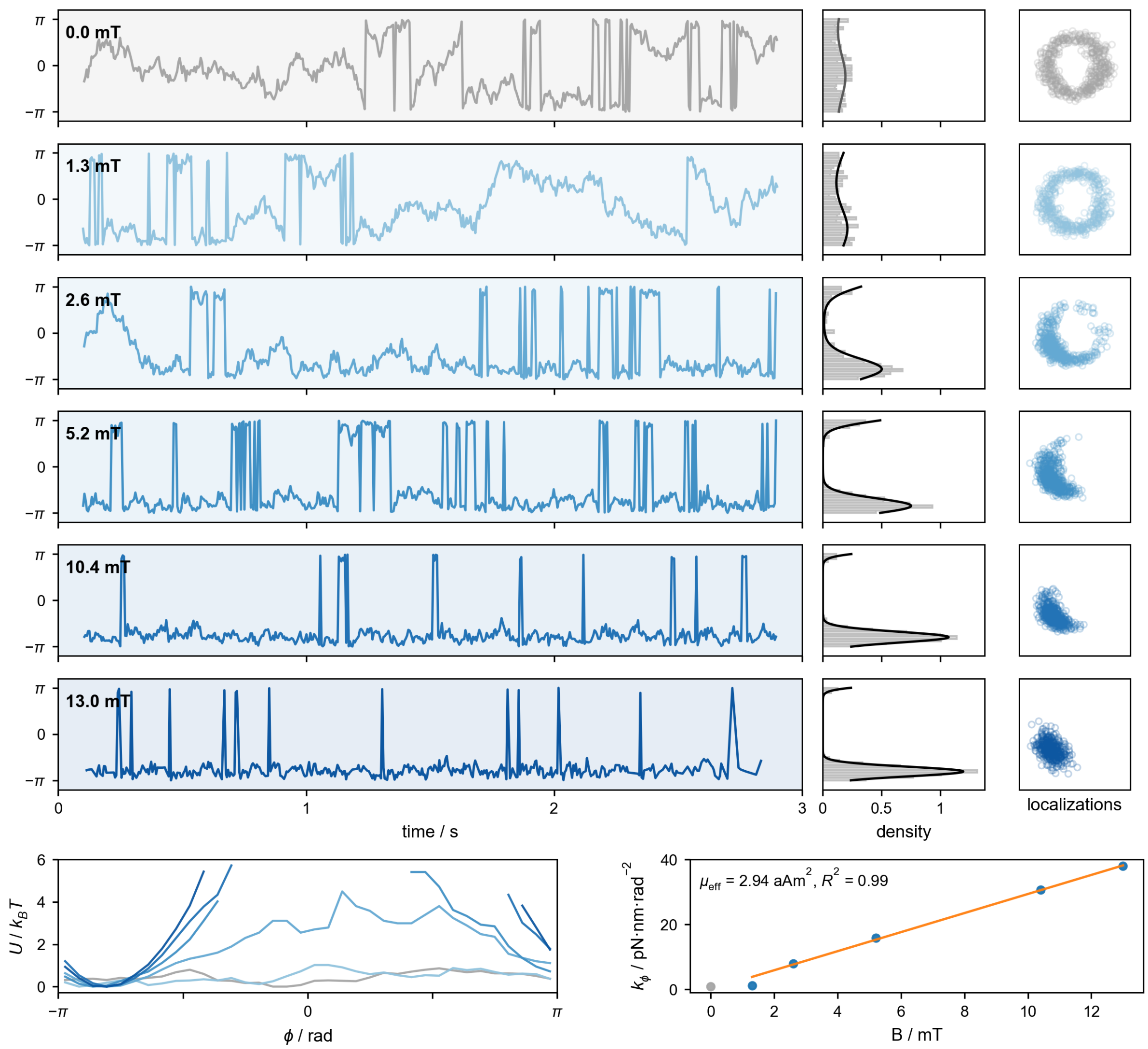

FIG. S29. **Cont. Exemplary 4-MNC MADONA rotor during clamping experiment**.

Trap stiffness as function of the applied field

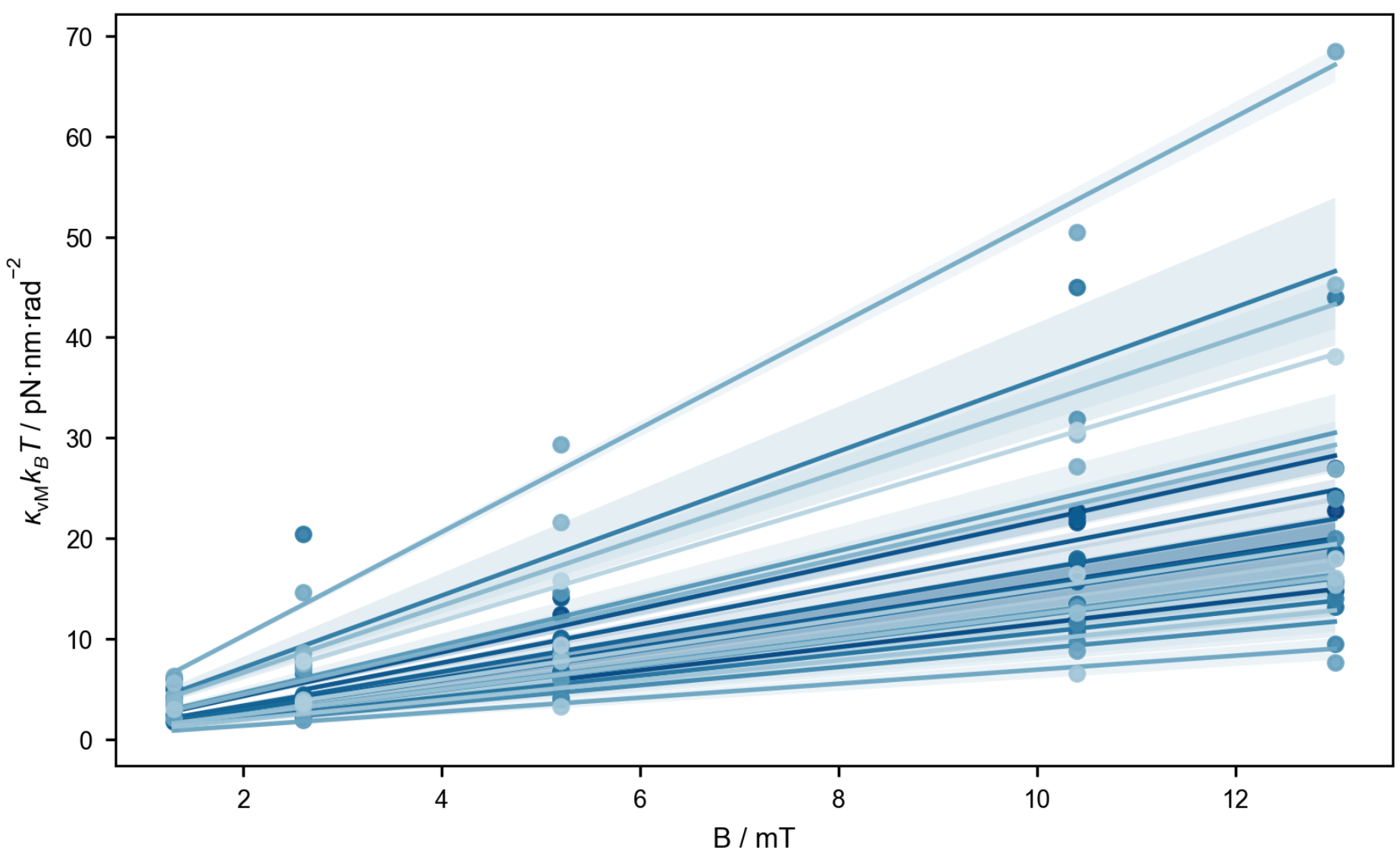

FIG. S30. **Trap stiffness as function of the applied field** for $n = 23$ 4-MNC MADONAs.

12. Monte Carlo simulations of magnetic properties of MADONAs

To capture the overal magnetic configuration of MADONAs in homogeneous magnetic fields and to estimate their net magnetization axis, magnetic moment and torques, we minimized the total magnetic energy. We used Metropolis Monte Carlo (MMC) energy minimization scheme for this purpose. The total magnetic energy $U_{\mathrm{i}}^{\mathrm{T}}$ of MNCs in external magnetic fields is given by a modified Stoner-Wohlfarth (SW) model for a single-domain MNC. The modfied SW model considers three energy terms: the Zeeman energy presenting particle-field interaction, the magnetic anisotropy energy for a cubic crystal (purely magnetocrystalline nature and ignoring K_0), and the magnetic dipole-dipole interaction energy, and is given by:

$$\begin{aligned} \frac{U_T}{k_BT} &= -\xi_i \boldsymbol{m}_i \cdot \boldsymbol{B} + \frac{K_1 V_i}{k_B T}[\alpha_1^2\alpha_2^2 + \alpha_2^2\alpha_3^2 + \alpha_3^2\alpha_1^2] \\ &+ (\frac{d_0}{r_{ij}})^3 \lambda \left\{ \boldsymbol{m_i} \cdot \boldsymbol{m_j} - 3(\boldsymbol{m_i} \cdot \boldsymbol{r_{ij}})(\boldsymbol{m_j} \cdot \boldsymbol{r_{ij}}) \right\}, \end{aligned} \tag{15}$$

with

$$\xi_i = \frac{m_i B}{k_B T}, \tag{16}$$

and

$$\lambda = \frac{\mu_0 {m_0}^2}{4\pi k_B T {d_0}^3}, \tag{17}$$

in which k_{B} is the Boltzmann's constant in J K^{-1}, T (295 k) is the temperature in K, d_{i} is the particle i core diameter in nm, $r_{\mathrm{ij}} = \|r_{\mathrm{ij}}\|$ is the magnitude of the vector drawn from particles i to j in nm (Figure 4d), ξ is the particle's dipole-field interaction parameter, λ is a dimensionless dipole-dipole coupling parameter, K_1 is the first-order anisotropy coefficient in J m^{-3}, m_0 is the average magnetic moment, d_0 is the average particle core diameter. Items shown in bold are the unit vectors (e.g. $\boldsymbol{m_i}$ is the magnetic moment unit vector of the particle i and $m_{\mathrm{i}}= \|m_{\mathrm{i}}\|$). $\alpha_1 = \cos(\theta_1)$, $\alpha_2 = \cos(\theta_2)$, and $\alpha_3 = \cos(\theta_3)$ are the cosines of the angles between the magetization vector and the principle crystallographic axes of the crystal (Fig. S31a).

Our $Co_{0.4}Zn_{0.2}Fe_{2.4}O_4$ MNCs with a cubic crystal strcucture have a cubic magnetic anisotropy which is given by the second term im eq. 19 (Fig. S7a). The dominance of the cubic magnetic anisotropy can further be deduced by looking at the remanence to saturation magnetization ($M_{\mathrm{r}}/M_{\mathrm{s}}$) ratio of magnetic hysteresis loops at 5 K (Fig. S31b). The $M_{\mathrm{r}}/M_{\mathrm{s}}$ = 0.76 falls within the regime where the cubic magnetic anisotropy governs. Cobalt-doped zinc ferrite MNCs have the first-order magnetic anisotropy constant $K_1 > 0$, thus having six magnetic easy axes (Fig. S31a).

We can simplify the anisotropy axis sytem from a sixfold to a fourfold symmetry with two magnetization easy directions: [100] and [010], since the magnetic actuation experiments are done on the x-y surface plane (i.e. $\theta_3 = 0$ and $\cos(\theta_3) = 0$) (Fig. S31a). By doing so, the cubic magnetic anisotropy energy term in eq. 19 will be simplified to:

$$\begin{gathered} \frac{E_a}{k_BT} = \frac{K_1V_i}{k_BT}(\alpha_1^2\alpha_2^2) = \frac{K_1V_i}{k_BT}\cos^2(\theta_1)\cos^2(\theta_2) \\ \theta_2 = \frac{\pi}{2} - \theta_1 \\ \cos^2(\theta_2) = sin^2(\theta_1) \\ \frac{E_a}{k_BT} = \frac{K_1V_i}{k_BT}\cos^2(\theta_1)\sin^2(\theta_1) \\ \frac{E_a}{k_BT} = \frac{K_1V_i}{4k_BT}\sin^2(2\theta_1) \end{gathered} \tag{18}$$

with

$$K_1 = 4K_{eff} \tag{19}$$

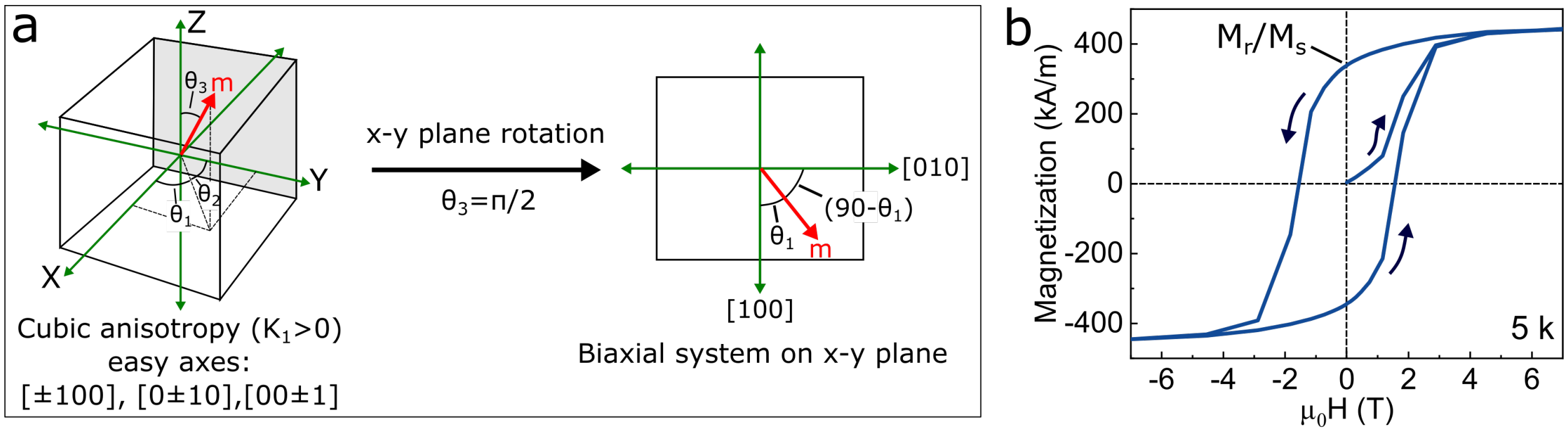

FIG. S31. **Magnetic anisotropy model of custom MNCs**.(a) Scheme of a single MNC with Cubic magnetic anisotropy and the first-order anisotropy constant K_1 with sixfold magnetization easy axes. Considering the magnetic rotation experiments in x-y plane, the symmetry from sixfold symmetry can be reduced to fourfold with two easy magnetization directions. This so-called biaxial system shows four energy minima at 0°, 90°, 180°, and 270°. (b) Magnetic hysteresis loops of individual MNCs measured at 5 K, showing the direction in which the magnetization loops are recorded.

Through this simplification, the in x-y plane cubic magnetic anisotropy resembles the uniaxial anisotropy with four energy minima at 0°, 90°, 180°, and 270°.

The MMC simulations are 2D in-plane, in which the particle' magnetic moment is considered as a magnetic macrospin, pointing out from its center of coordination in a Cartesian x-y coordination system as shown in Fig. S31a.

At the beginning of each MMC simulation, the particle's magnetic moments and easy axes are randomly chosen in the x-y plane. Both vectors are set to be aligned to fulfill the magnetization in our ferrimagnetic MNCs with permanent dipole moment, where the magnetic moment is coupled to the easy axis. Next and at each MC sweep, a particle is chosen randomly and its magnetic moment and easy axis are rotated randomly for a maximum of 10° and 5°, respectively. The rotation of moment and easy axis vectors occurs in the same direction, with maximum 10° phase-lag between them. As the x-y coordinate will be set to the direction of the easy axis at the start of each MC simulation, the random rotation of the easy axis corresponds to the random physical rotation of MNCs (Figure 4d) on the 6HB. Due to ferrimagnetic nature of our MNCs, we considered that the alignment of the particle's moment with field requires a physical rotation of MNCs after being bound to the bundles (Figure 4d). As the MNCs are coupled to the 6HBs through three 8 nt double-stranded DNA, we hypothesize that the rotational freedom of bound MNCs is limited to a maximum of 90° toward the field. To give a full picture, we performed MMC simulations at 45°, 60°, and 90° rotational freedom. Next, at each new configuration, the total free energy is calculated and compared with the old configuration. If the total energy is reduced, the new configutaion is accepted. If the total energy is increased, the Metroplois criterion is applied and depending on the outcome, either the old or new configuration is accepted.

The following experimentally determined parameters were plugged into the MC simulations: M_s = 269 kA m^{-1}, K_{eff} = 50200 J m^{-3}. The particle size distribution was considerd to be log-normal with μ = 2.815 and σ = 0.09, as obtained from TEM analyses (Figure 1). Each MC simulation is iterated for 500.000 MC sweeps to reach equilibrium. Uniform magnetic fields along the [0-10] direction with the field strength of 9 mT were used. From the MC simulations, we obtained equailibrium magnetic configuration, effective magnetic moment at R_{c-c} = 64 nm, and maximum magnetic torques. Our MC simulations capture the magnetic configuration of MADONAs in the clamping experiments.

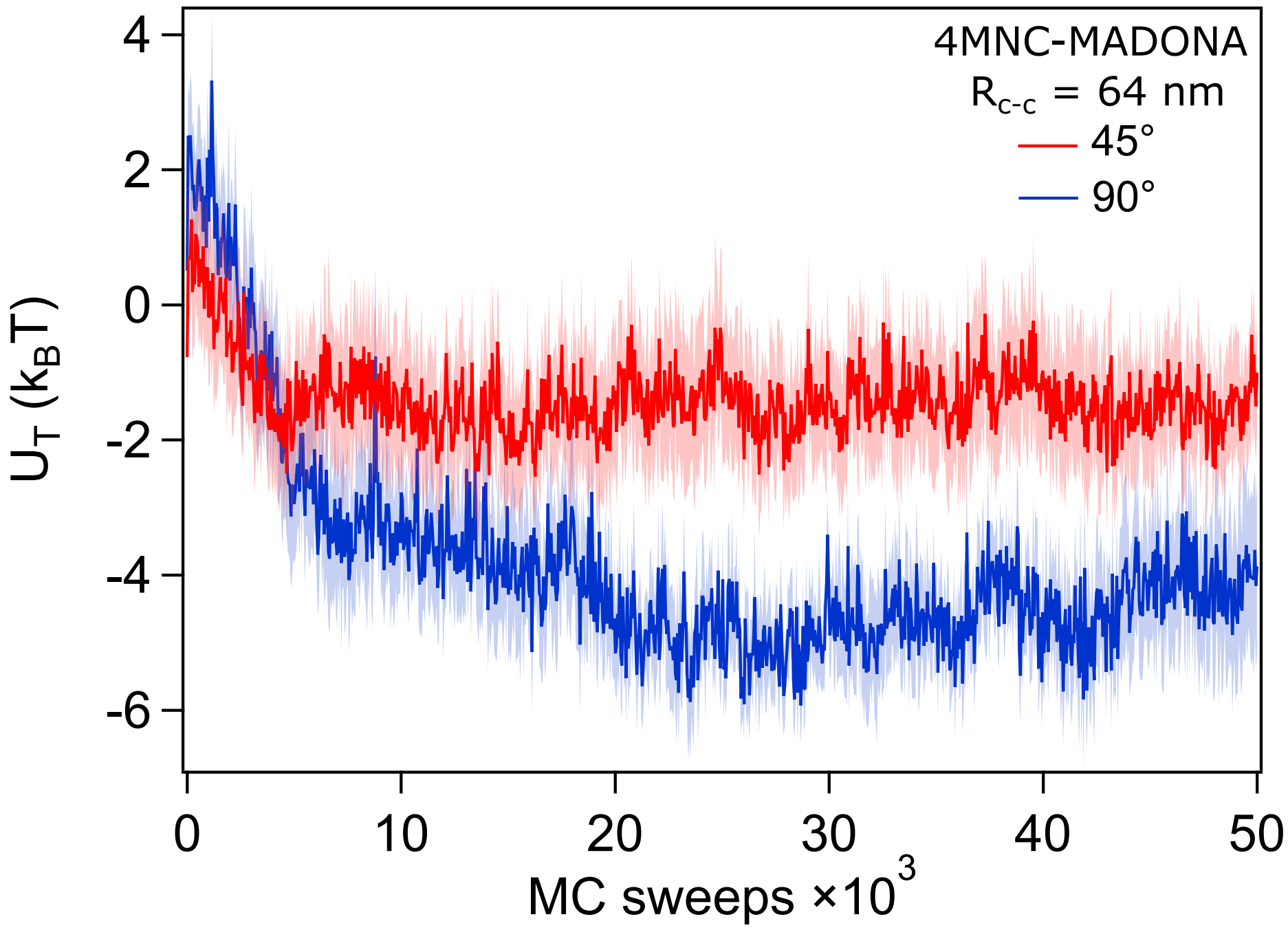

FIG. S32. **Total magnetic energy minimization using MMC simulations**. Changes in the total magnetic energy of 4-MNC MADONA at R_{c-c} = 64 nm as a function of MMC sweeps at 45 and 90 ° rotational freedom. Data shown are the mean $\pm$ s.e.m of fifteen MC simulations at 9 mT magnetic fields. Errors are shown as shaded bands. Data are interpolated to 1000 points (cubic spline) for the sake of clarity and presentation. Only the first 50 k MMC sweeps out of the total 500 k are shown.

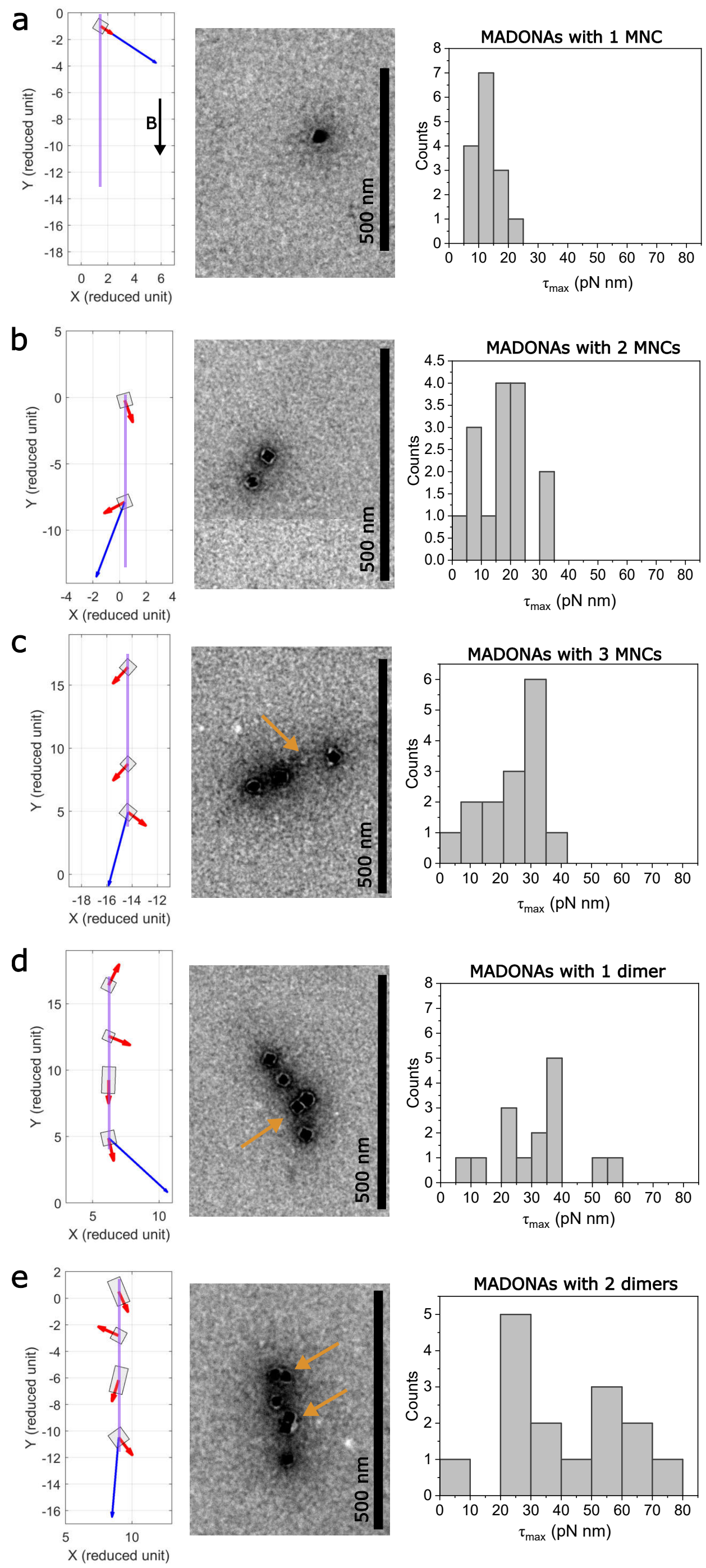

FIG. S33. **Monte Carlo simulations on 4-MNC MADONAs with non-stoichiometric number of bound MNCs and dimers**. (a) MADONAs with only one MNC bound. (b) MADONAs having only two particle binding sites occupied. (c) MADONAs with three particle binding sites being occupied. (d) MADONAs showing full occupancy yet having one dimer in one binding site instead of monomeric MNCs. (e) MADONAs with full occupancy and having two dimers in two of their binding sites instead of monomerric MNCs. In all panels, the left figure shows a typical MMC simulation box with the reduced unit equals to the mean particle size. The MMNCs are shown as gray squares (dimers as gray rectangulars), their macro spin as red arrows and the overall magnetization axis of the MADONA as blue arrows. The purple line represents the 216 nm section of 6HB bundles on which binding patches are designed (Fig. S2). The particle coordinates are chosen randmoly within the simulation box. The middle figures are negative-stain TEM micrographs from Fig.S10. The yellow arrows highlight unbound binding sites and dimers. The right figures are torques histograms obtained from the MMC simulations at 9 mT as described in the main text.

13. 4-MNC MADONAs driven in a low-viscosity buffer

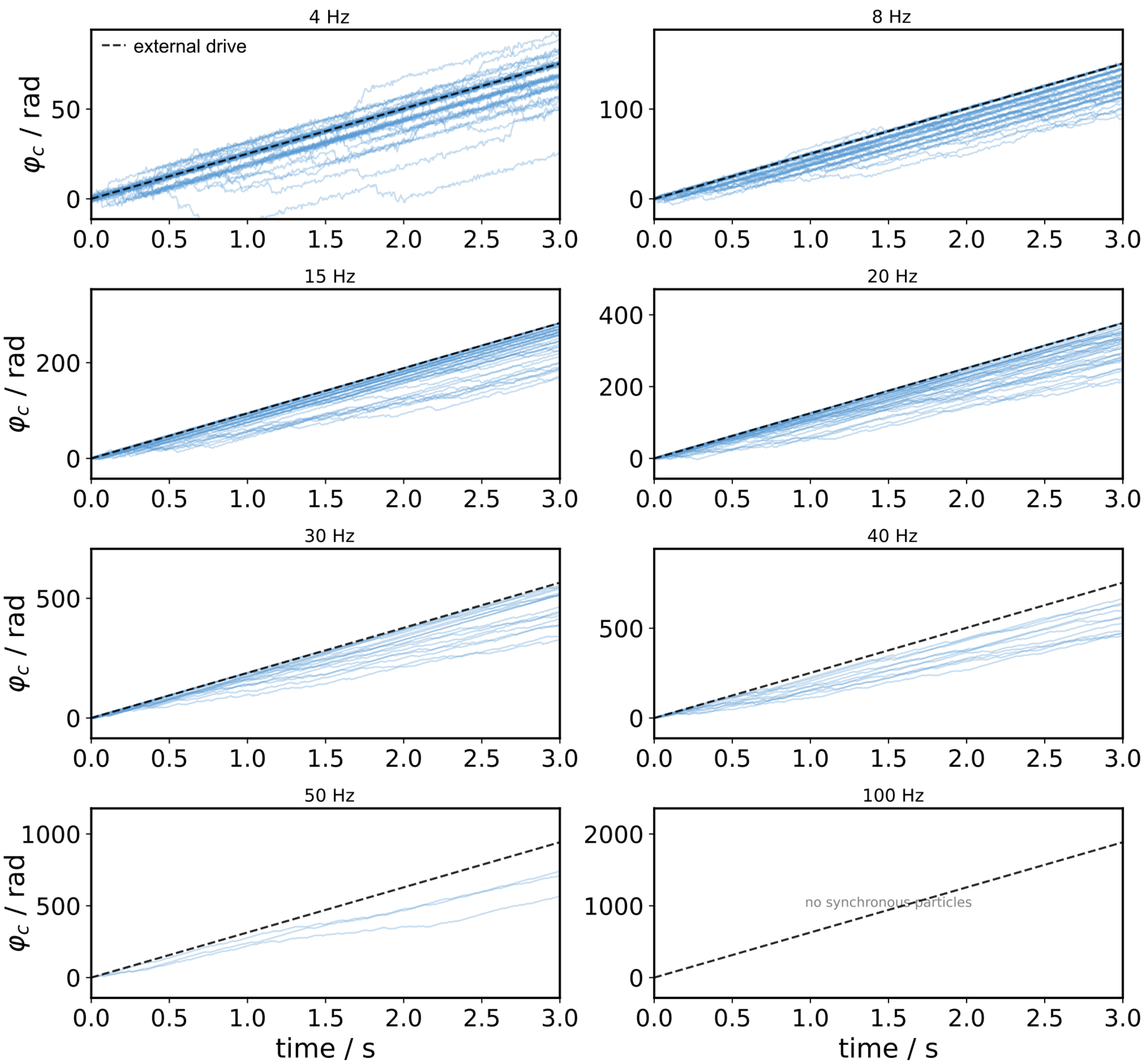

FIG. S34. **Trajectories of 4-MNC MADONAs driven by rotating magnetic fields in low-viscosity buffer.** Rotor trajectories from different fields of view during actuation at $B = 9$ mT and different driving frequencies measured in a buffer at water viscosity (1x TE supplemented with 500 mM NaCl). Individual trajectories are shown in light blue, and the dashed black line indicates the external drive. With increasing driving frequency, fewer rotors remain synchronized.

14. Scaffold and staple sequences

$7,249$ basepair scaffold version used for the 6hb structues:

```
1         11        21        31        41        51        61        71
AAGCTTGGCACTGGCCGTCGTTTTACAACGTCGTGACTGGGAAAACCCTGGCGTTACCCAACTTAATCGCCTTGCAGCAC
81        91        101       111       121       131       141       151
ATCCCCCTTTCGCCAGCTGGCGTAATAGCGAAGAGGCCCGCACCGATCGCCCTTCCCAACAGTTGCGCAGCCTGAATGGC
161       171       181       191       201       211       221       231
GAATGGCGCTTTGCCTGGTTTCCGGCACCAGAAGCGGTGCCGGAAAGCTGGCTGGAGTGCGATCTTCCTGAGGCCGATAC
241       251       261       271       281       291       301       311
TGTCGTCGTCCCCTCAAACTGGCAGATGCACGGTTACGATGCGCCCATCTACACCAACGTGACCTATCCCATTACGGTCA
321       331       341       351       361       371       381       391
ATCCGCCGTTTGTTCCCACGGAGAATCCGACGGGTTGTTACTCGCTCACATTTAATGTTGATGAAAGCTGGCTACAGGAA
401       411       421       431       441       451       461       471
GGCCAGACGCGAATTATTTTTGATGGCGTTCCTATTGGTTAAAAAATGAGCTGATTTAACAAAAATTTAATGCGAATTTT
481       491       501       511       521       531       541       551
AACAAAATATTAACGTTTACAATTTAAATATTTGCTTATACAATCTTCCTGTTTTTGGGGCTTTTCTGATTATCAACCGG
561       571       581       591       601       611       621       631
GGTACATATGATTGACATGCTAGTTTTACGATTACCGTTCATCGATTCTCTTGTTTGCTCCAGACTCTCAGGCAATGACC
641       651       661       671       681       691       701       711
TGATAGCCTTTGTAGATCTCTCAAAAATAGCTACCCTCTCCGGCATTAATTTATCAGCTAGAACGGTTGAATATCATATT
721       731       741       751       761       771       781       791
GATGGTGATTTGACTGTCTCCGGCCTTTCTCACCCTTTTGAATCTTTACCTACACATTACTCAGGCATTGCATTTAAAAT
801       811       821       831       841       851       861       871
ATATGAGGGTTCTAAAAATTTTTATCCTTGCGTTGAAATAAAGGCTTCTCCCGCAAAAGTATTACAGGGTCATAATGTTT
881       891       901       911       921       931       941       951
TTGGTACAACCGATTTAGCTTTATGCTCTGAGGCTTTATTGCTTAATTTTGCTAATTCTTTGCCTTGCCTGTATGATTTA
961       971       981       991       1001      1011      1021      1031
TTGGATGTTAATGCTACTACTATTAGTAGAATTGATGCCACCTTTTCAGCTCGCGCCCCAAATGAAAATATAGCTAAACA
1041      1051      1061      1071      1081      1091      1101      1111
GGTTATTGACCATTTGCGAAATGTATCTAATGGTCAAACTAAATCTACTCGTTCGCAGAATTGGGAATCAACTGTTATAT
1121      1131      1141      1151      1161      1171      1181      1191
GGAATGAAACTTCCAGACACCGTACTTTAGTTGCATATTTAAAACATGTTGAGCTACAGCATTATATTCAGCAATTAAGC
1201      1211      1221      1231      1241      1251      1261      1271
TCTAAGCCATCCGCAAAAATGACCTCTTATCAAAAGGAGCAATTAAAGGTACTCTCTAATCCTGACCTGTTGGAGTTTGC
1281      1291      1301      1311      1321      1331      1341      1351
TTCCGGTCTGGTTCGCTTTGAAGCTCGAATTAAAACGCGATATTTGAAGTCTTTCGGGCTTCCTCTTAATCTTTTTGATG
1361      1371      1381      1391      1401      1411      1421      1431
CAATCCGCTTTGCTTCTGACTATAATAGTCAGGGTAAAGACCTGATTTTTGATTTATGGTCATTCTCGTTTTCTGAACTG
1441      1451      1461      1471      1481      1491      1501      1511
TTTAAAGCATTTGAGGGGGATTCAATGAATATTTATGACGATTCCGCAGTATTGGACGCTATCCAGTCTAAACATTTTAC
1521      1531      1541      1551      1561      1571      1581      1591
TATTACCCCCTCTGGCAAAACTTCTTTTGCAAAAGCCTCTCGCTATTTTGGTTTTTATCGTCGTCTGGTAAACGAGGGTT
1601      1611      1621      1631      1641      1651      1661      1671
ATGATAGTGTTGCTCTTACTATGCCTCGTAATTCCTTTTGGCGTTATGTATCTGCATTAGTTGAATGTGGTATTCCTAAA
1681      1691      1701      1711      1721      1731      1741      1751
TCTCAACTGATGAATCTTTCTACCTGTAATAATGTTGTTCCGTTAGTTCGTTTTATTAACGTAGATTTTTCTTCCCAACG
1761      1771      1781      1791      1801      1811      1821      1831
TCCTGACTGGTATAATGAGCCAGTTCTTAAAATCGCATAAGGTAATTCACAATGATTAAAGTTGAAATTAAACCATCTCA
1841      1851      1861      1871      1881      1891      1901      1911
AGCCCAATTTACTACTCGTTCTGGTGTTTCTCGTCAGGGCAAGCCTTATTCACTGAATGAGCAGCTTTGTTACGTTGATT
1921      1931      1941      1951      1961      1971      1981      1991
TGGGTAATGAATATCCGGTTCTTGTCAAGATTACTCTTGATGAAGGTCAGCCAGCCTATGCGCCTGGTCTGTACACCGTT
2001      2011      2021      2031      2041      2051      2061      2071
CATCTGTCCTCTTTCAAAGTTGGTCAGTTCGGTTCCCTTATGATTGACCGTCTGCGCCTCGTTCCGGCTAAGTAACATGG
```

```
2081      2091      2101      2111      2121      2131      2141      2151
AGCAGGTCGCGGATTTCGACACAATTTATCAGGCGATGATACAAATCTCCGTTGTACTTTGTTTCGCGCTTGGTATAATC
2161      2171      2181      2191      2201      2211      2221      2231
GCTGGGGGTCAAAGATGAGTGTTTTAGTGTATTCTTTTGCCTCTTTCGTTTTAGGTTGGTGCCTTCGTAGTGGCATTACG
2241      2251      2261      2271      2281      2291      2301      2311
TATTTTACCCGTTTAATGGAAACTTCCTCATGAAAAAGTCTTTAGTCCTCAAAGCCTCTGTAGCCGTTGCTACCCTCGTT
2321      2331      2341      2351      2361      2371      2381      2391
CCGATGCTGTCTTTCGCTGCTGAGGGTGACGATCCCGCAAAAGCGGCCTTTAACTCCCTGCAAGCCTCAGCGACCGAATA
2401      2411      2421      2431      2441      2451      2461      2471
TATCGGTTATGCGTGGGCGATGGTTGTTGTCATTGTCGGCGCAACTATCGGTATCAAGCTGTTTAAGAAATTCACCTCGA
2481      2491      2501      2511      2521      2531      2541      2551
AAGCAAGCTGATAAACCGATACAATTAAAGGCTCCTTTTGGAGCCTTTTTTTTGGAGATTTTCAACGTGAAAAAATTATT
2561      2571      2581      2591      2601      2611      2621      2631
ATTCGCAATTCCTTTAGTTGTTCCTTTCTATTCTCACTCCGCTGAAACTGTTGAAAGTTGTTTAGCAAAATCCCATACAG
2641      2651      2661      2671      2681      2691      2701      2711
AAAATTCATTTACTAACGTCTGGAAAGACGACAAAACTTTAGATCGTTACGCTAACTATGAGGGCTGTCTGTGGAATGCT
2721      2731      2741      2751      2761      2771      2781      2791
ACAGGCGTTGTAGTTTGTACTGGTGACGAAACTCAGTGTTACGGTACATGGGTTCCTATTGGGCTTGCTATCCCTGAAAA
2801      2811      2821      2831      2841      2851      2861      2871
TGAGGGTGGTGGCTCTGAGGGTGGCGGTTCTGAGGGTGGCGGTTCTGAGGGTGGCGGTACTAAACCTCCTGAGTACGGTG
2881      2891      2901      2911      2921      2931      2941      2951
ATACACCTATTCCGGGCTATACTTATATCAACCCTCTCGACGGCACTTATCCGCCTGGTACTGAGCAAAACCCCGCTAAT
2961      2971      2981      2991      3001      3011      3021      3031
CCTAATCCTTCTCTTGAGGAGTCTCAGCCTCTTAATACTTTCATGTTTCAGAATAATAGGTTCCGAAATAGGCAGGGGGC
3041      3051      3061      3071      3081      3091      3101      3111
ATTAACTGTTTATACGGGCACTGTTACTCAAGGCACTGACCCCGTTAAAACTTATTACCAGTACACTCCTGTATCATCAA
3121      3131      3141      3151      3161      3171      3181      3191
AAGCCATGTATGACGCTTACTGGAACGGTAAATTCAGAGACTGCGCTTTCCATTCTGGCTTTAATGAGGATTTATTTGTT
3201      3211      3221      3231      3241      3251      3261      3271
TGTGAATATCAAGGCCAATCGTCTGACCTGCCTCAACCTCCTGTCAATGCTGGCGGCGGCTCTGGTGGTGGTTCTGGTGG
3281      3291      3301      3311      3321      3331      3341      3351
CGGCTCTGAGGGTGGTGGCTCTGAGGGTGGCGGTTCTGAGGGTGGCGGCTCTGAGGGAGGCGGTTCCGGTGGTGGCTCTG
3361      3371      3381      3391      3401      3411      3421      3431
GTTCCGGTGATTTTGATTATGAAAAGATGGCAAACGCTAATAAGGGGGCTATGACCGAAAATGCCGATGAAAACGCGCTA
3441      3451      3461      3471      3481      3491      3501      3511
CAGTCTGACGCTAAAGGCAAACTTGATTCTGTCGCTACTGATTACGGTGCTGCTATCGATGGTTTCATTGGTGACGTTTC
3521      3531      3541      3551      3561      3571      3581      3591
CGGCCTTGCTAATGGTAATGGTGCTACTGGTGATTTTGCTGGCTCTAATTCCCAAATGGCTCAAGTCGGTGACGGTGATA
3601      3611      3621      3631      3641      3651      3661      3671
ATTCACCTTTAATGAATAATTTCCGTCAATATTTACCTTCCCTCCCTCAATCGGTTGAATGTCGCCCTTTTGTCTTTGGC
3681      3691      3701      3711      3721      3731      3741      3751
GCTGGTAAACCATATGAATTTTCTATTGATTGTGACAAAATAAACTTATTCCGTGGTGTCTTTGCGTTTCTTTTATATGT
3761      3771      3781      3791      3801      3811      3821      3831
TGCCACCTTTATGTATGTATTTTCTACGTTTGCTAACATACTGCGTAATAAGGAGTCTTAATCATGCCAGTTCTTTTGGG
3841      3851      3861      3871      3881      3891      3901      3911
TATTCCGTTATTATTGCGTTTCCTCGGTTTCCTTCTGGTAACTTTGTTCGGCTATCTGCTTACTTTTCTTAAAAAGGGCT
3921      3931      3941      3951      3961      3971      3981      3991
TCGGTAAGATAGCTATTGCTATTTCATTGTTTCTTGCTCTTATTATTGGGCTTAACTCAATTCTTGTGGGTTATCTCTCT
4001      4011      4021      4031      4041      4051      4061      4071
GATATTAGCGCTCAATTACCCTCTGACTTTGTTCAGGGTGTTCAGTTAATTCTCCCGTCTAATGCGCTTCCCTGTTTTTA
4081      4091      4101      4111      4121      4131      4141      4151
TGTTATTCTCTCTGTAAAGGCTGCTATTTTCATTTTTGACGTTAAACAAAAAATCGTTTCTTATTTGGATTGGGATAAAT
4161      4171      4181      4191      4201      4211      4221      4231
AATATGGCTGTTTATTTTGTAACTGGCAAATTAGGCTCTGGAAAGACGCTCGTTAGCGTTGGTAAGATTCAGGATAAAAT
4241      4251      4261      4271      4281      4291      4301      4311
TGTAGCTGGGTGCAAAATAGCAACTAATCTTGATTTAAGGCTTCAAAACCTCCCGCAAGTCGGGAGGTTCGCTAAAACGC
```

```
4321      4331      4341      4351      4361      4371      4381      4391
CTCGCGTTCTTAGAATACCGGATAAGCCTTCTATATCTGATTTGCTTGCTATTGGGCGCGGTAATGATTCCTACGATGAA
4401      4411      4421      4431      4441      4451      4461      4471
AATAAAAACGGCTTGCTTGTTCTCGATGAGTGCGGTACTTGGTTTAATACCCGTTCTTGGAATGATAAGGAAAGACAGCC
4481      4491      4501      4511      4521      4531      4541      4551
GATTATTGATTGGTTTCTACATGCTCGTAAATTAGGATGGGATATTATTTTTCTTGTTCAGGACTTATCTATTGTTGATA
4561      4571      4581      4591      4601      4611      4621      4631
AACAGGCGCGTTCTGCATTAGCTGAACATGTTGTTTATTGTCGTCGTCTGGACAGAATTACTTTACCTTTTGTCGGTACT
4641      4651      4661      4671      4681      4691      4701      4711
TTATATTCTCTTATTACTGGCTCGAAAATGCCTCTGCCTAAATTACATGTTGGCGTTGTTAAATATGGCGATTCTCAATT
4721      4731      4741      4751      4761      4771      4781      4791
AAGCCCTACTGTTGAGCGTTGGCTTTATACTGGTAAGAATTTGTATAACGCATATGATACTAAACAGGCTTTTTCTAGTA
4801      4811      4821      4831      4841      4851      4861      4871
ATTATGATTCCGGTGTTTATTCTTATTTAACGCCTTATTTATCACACGGTCGGTATTTCAAACCATTAAATTTAGGTCAG
4881      4891      4901      4911      4921      4931      4941      4951
AAGATGAAATTAACTAAAATATATTTGAAAAAGTTTTCTCGCGTTCTTTGTCTTGCGATTGGATTTGCATCAGCATTTAC
4961      4971      4981      4991      5001      5011      5021      5031
ATATAGTTATATAACCCAACCTAAGCCGGAGGTTAAAAAGGTAGTCTCTCAGACCTATGATTTTGATAAATTCACTATTG
5041      5051      5061      5071      5081      5091      5101      5111
ACTCTTCTCAGCGTCTTAATCTAAGCTATCGCTATGTTTTCAAGGATTCTAAGGGAAAATTAATTAATAGCGACGATTTA
5121      5131      5141      5151      5161      5171      5181      5191
CAGAAGCAAGGTTATTCACTCACATATATTGATTTATGTACTGTTTCCATTAAAAAAGGTAATTCAAATGAAATTGTTAA
5201      5211      5221      5231      5241      5251      5261      5271
ATGTAATTAATTTTGTTTTCTTGATGTTTGTTTCATCATCTTCTTTTGCTCAGGTAATTGAAATGAATAATTCGCCTCTG
5281      5291      5301      5311      5321      5331      5341      5351
CGCGATTTTGTAACTTGGTATTCAAAGCAATCAGGCGAATCCGTTATTGTTTCTCCCGATGTAAAAGGTACTGTTACTGT
5361      5371      5381      5391      5401      5411      5421      5431
ATATTCATCTGACGTTAAACCTGAAAATCTACGCAATTTCTTTATTTCTGTTTTACGTGCAAATAATTTTGATATGGTAG
5441      5451      5461      5471      5481      5491      5501      5511
GTTCTAACCCTTCCATTATTCAGAAGTATAATCCAAACAATCAGGATTATATTGATGAATTGCCATCATCTGATAATCAG
5521      5531      5541      5551      5561      5571      5581      5591
GAATATGATGATAATTCCGCTCCTTCTGGTGGTTTCTTTGTTCCGCAAAATGATAATGTTACTCAAACTTTTAAAATTAA
5601      5611      5621      5631      5641      5651      5661      5671
TAACGTTCGGGCAAAGGATTTAATACGAGTTGTCGAATTGTTTGTAAAGTCTAATACTTCTAAATCCTCAAATGTATTAT
5681      5691      5701      5711      5721      5731      5741      5751
CTATTGACGGCTCTAATCTATTAGTTGTTAGTGCTCCTAAAGATATTTTAGATAACCTTCCTCAATTCCTTTCAACTGTT
5761      5771      5781      5791      5801      5811      5821      5831
GATTTGCCAACTGACCAGATATTGATTGAGGGTTTGATATTTGAGGTTCAGCAAGGTGATGCTTTAGATTTTTCATTTGC
5841      5851      5861      5871      5881      5891      5901      5911
TGCTGGCTCTCAGCGTGGCACTGTTGCAGGCGGTGTTAATACTGACCGCCTCACCTCTGTTTTATCTTCTGCTGGTGGTT
5921      5931      5941      5951      5961      5971      5981      5991
CGTTCGGTATTTTTAATGGCGATGTTTTAGGGCTATCAGTTCGCGCATTAAAGACTAATAGCCATTCAAAAATATTGTCT
6001      6011      6021      6031      6041      6051      6061      6071
GTGCCACGTATTCTTACGCTTTCAGGTCAGAAGGGTTCTATCTCTGTTGGCCAGAATGTCCCTTTTATTACTGGTCGTGT
6081      6091      6101      6111      6121      6131      6141      6151
GACTGGTGAATCTGCCAATGTAAATAATCCATTTCAGACGATTGAGCGTCAAAATGTAGGTATTTCCATGAGCGTTTTTC
6161      6171      6181      6191      6201      6211      6221      6231
CTGTTGCAATGGCTGGCGGTAATATTGTTCTGGATATTACCAGCAAGGCCGATAGTTTGAGTTCTTCTACTCAGGCAAGT
6241      6251      6261      6271      6281      6291      6301      6311
GATGTTATTACTAATCAAAGAAGTATTGCTACAACGGTTAATTTGCGTGATGGACAGACTCTTTTACTCGGTGGCCTCAC
6321      6331      6341      6351      6361      6371      6381      6391
TGATTATAAAAACACTTCTCAGGATTCTGGCGTACCGTTCCTGTCTAAAATCCCTTTAATCGGCCTCCTGTTTAGCTCCC
6401      6411      6421      6431      6441      6451      6461      6471
GCTCTGATTCTAACGAGGAAAGCACGTTATACGTGCTCGTCAAAGCAACCATAGTACGCGCCCTGTAGCGGCGCATTAAG
6481      6491      6501      6511      6521      6531      6541      6551
CGCGGCGGGTGTGGTGGTTACGCGCAGCGTGACCGCTACACTTGCCAGCGCCCTAGCGCCCGCTCCTTTCGCTTTCTTCC
```

```
6561      6571      6581      6591      6601      6611      6621      6631
CTTCCTTTCTCGCCACGTTCGCCGGCTTTCCCCGTCAAGCTCTAAATCGGGGGCTCCCTTTAGGGTTCCGATTTAGTGCT
6641      6651      6661      6671      6681      6691      6701      6711
TTACGGCACCTCGACCCCAAAAAACTTGATTTGGGTGATGGTTCACGTAGTGGGCCATCGCCCTGATAGACGGTTTTTCG
6721      6731      6741      6751      6761      6771      6781      6791
CCCTTTGACGTTGGAGTCCACGTTCTTTAATAGTGGACTCTTGTTCCAAACTGGAACAACACTCAACCCTATCTCGGGCT
6801      6811      6821      6831      6841      6851      6861      6871
ATTCTTTTGATTTATAAGGGATTTTGCCGATTTCGGAACCACCATCAAACAGGATTTTCGCCTGCTGGGGCAAACCAGCG
6881      6891      6901      6911      6921      6931      6941      6951
TGGACCGCTTGCTGCAACTCTCTCAGGGCCAGGCGGTGAAGGGCAATCAGCTGTTGCCCGTCTCACTGGTGAAAAGAAAA
6961      6971      6981      6991      7001      7011      7021      7031
ACCACCCTGGCGCCCAATACGCAAACCGCCTCTCCCCGCGCGTTGGCCGATTCATTAATGCAGCTGGCACGACAGGTTTC
7041      7051      7061      7071      7081      7091      7101      7111
CCGACTGGAAAGCGGGCAGTGAGCGCAACGCAATTAATGTGAGTTAGCTCACTCATTAGGCACCCCAGGCTTTACACTTT
7121      7131      7141      7151      7161      7171      7181      7191
ATGCTTCCGGCTCGTATGTTGTGTGGAATTGTGAGCGGATAACAATTTCACACAGGAAACAGCTATGACCATGATTACGA
7201      7211      7221      7231      7241
ATTCGAGCTCGGTACCCGGGGATCCTCTAGAGTCGACCTGCAGGCATGC
```

Staple sequences of the basic 6hb DNA origami nanostructure

Staple sequences of the 6HB base structure used as MNC binding platform. Staple sequences are denoted in the form Name, starting helix [base position]end helix[base position], DNA sequence.

TABLE I: Extension sequences

Type	Name	Sequence
basic 6HB	1[154]3[167]	GCAACGGCGCCCACGCCTTGAGTAACAG
basic 6HB	0[832]3[825]	AAGCAAACGAGCCGAAACGCAATAATAAAGAATAA
basic 6HB	1[553]3[566]	AACAAAGAACAAACGAATCCTTGAAAAC
basic 6HB	1[1036]3[1049]	CTTTGATAGCACTACGACGACAATAAAC
basic 6HB	0[643]3[636]	TATAATGCTGCAAGCAAAAGGGCGACATCTGAGAG
basic 6HB	1[280]3[293]	AAACACTTTGAAAATTGCTCAGTACCAG
basic 6HB	1[343]3[356]	TATCATCGTTTCAGTGTATCACCGTACT
basic 6HB	1[1015]3[1028]	TTAACCGAGAGCCGGGTAAAGTAATTCT
basic 6HB	0[412]3[405]	CATACAGGATAGGTGTAGCGCGTTTTCAAACCGCC
basic 6HB	0[286]3[279]	AAGGATATAGGAACAACCGCCACCCTCACGGGGTT
basic 6HB	0[622]3[615]	GTTTTAAAGCTGGCATTGAGGGAGGGAAGAATTTA
basic 6HB	1[322]3[335]	ACAAAGTAAAGGAAATAAGTATAGCCCG
basic 6HB	1[448]3[461]	TGAAAGACACAGACAATAGGAACCCATG
basic 6HB	0[370]3[363]	CCTCAGATCGGATTTAGCGTTTGCCATCCAGGAGG
basic 6HB	0[580]3[573]	TCCATATGTTGGGAAAGGTGAATTATCAATAGCGA
basic 6HB	1[133]3[146]	GACAGCATTCGGTCATAAGTTTTAACGG
basic 6HB	1[511]3[524]	TAATCTTCATTTGACCTTGCTTCTGTAA
basic 6HB	1[532]3[545]	ATTCATTATTAATTCTATTAATTAATTT
basic 6HB	1[427]3[440]	GGGAACCGCGTAACATTTTCAGGGATAG
basic 6HB	0[244]3[237]	TGCCTGATTCGCATAACCACCACCAGAGTATTAAG
basic 6HB	1[1099]3[1112]	ATATCCACTGGTCATCCTGAACAAGAAA
basic 6HB	1[889]3[902]	AAAACCATTGCGGACCAGTATAAAGCCA
basic 6HB	1[700]3[713]	AGGACGTTTTAACGCCAATCGCAAGACA
basic 6HB	0[748]3[741]	CCAGACCCGGGTACCATACATAAAGGTGCAAATAT
basic 6HB	1[196]3[209]	AGGAAGTCAGCTTGCCTATTTCGGAACC
basic 6HB	1[175]3[188]	AGGACTAGCCGACAATAAACAGTTAATG
basic 6HB	0[538]3[531]	TAGTTTGGAAACCATAGAGCCAGCAAAAATCGTCG
basic 6HB	0[979]3[972]	TACGCCAGGTTTGCCTAATATCAGAGAGCAGAGGC
basic 6HB	0[874]3[867]	ATAAATCGTGAGCTGTAAGCAGATAGCCAGTATCA
basic 6HB	0[790]3[783]	AAGACTTCCTGTGTAAGACTCCTTATTATGGTTTG
basic 6HB	1[385]3[398]	CATGTTAAATGAATACCGCCACCCTCAG

Continued on next page

Type	Name	Sequence
basic 6HB	0[391]3[384]	TAGCAAAACGGCGGTTTCGGTCATAGCCCCTCAGA
basic 6HB	0[223]3[216]	AAGATTCAACGTTACAGCATTGACAGGATATTATT
basic 6HB	0[916]3[909]	CCCCCTCTTCCAGTGAAATAGCAATAGCACGCTCA
basic 6HB	0[853]3[846]	TACCCTGAAAGCCTGTTACCAGAAGGAAATTACTA
basic 6HB	1[1057]3[1070]	CTTGCCTGTTATCTTCAGCTAATGCAGA
basic 6HB	1[259]3[272]	AAGAGGCGCTCCAAAGGATTAGGATTAG
basic 6HB	0[181]3[174]	CATCAATCCCAAAAGATATTCACAAACATGCCCGT
basic 6HB	0[265]3[258]	CCTCATAATCAGCTCACCCTCAGAGCCGCAAGAGA
basic 6HB	0[1063]3[1056]	GAGCACGAGAGAGTGAGAGAATAACATAAACATGT
basic 6HB	1[574]3[587]	AATAAGGCTGAGCATAGCTTAGATTAAG
basic 6HB	1[595]3[608]	AAACACCCGAATTAGAAGAGTCAATAGT
basic 6HB	0[328]3[321]	GACCCTGGCCAGCTAGCCACCACCGGAAGGTTGAT
basic 6HB	0[349]3[342]	CGGTTGTATGTGAGAATCAAAATCACCGGAATAGG
basic 6HB	1[973]3[986]	TCAGTGAAGACTTTATTTTCGAGCCAGT
basic 6HB	0[1105]3[1098]	CTTAATGGCGAAAAATTTTTTGTTTAACAGATAAG
basic 6HB	1[301]3[314]	AGCGATTGCGAATAAGTGCCGTCGAGAG
basic 6HB	1[826]3[839]	AAAGGAATGGCAATACACCGGAATCATA
basic 6HB	1[616]3[629]	ATTGGGCAGTTACATCAAAATCATAGGT
basic 6HB	0[664]3[657]	GCTTAGATAACGCCATGGTTTACCAGCGCTCCGGC
basic 6HB	1[637]3[650]	TTTCAACGCCTGATACTACCTTTTTAAC
basic 6HB	1[994]3[1007]	AGAGTCTATTTGAGGAATATAAAGTACC
basic 6HB	0[475]3[468]	GGGCGCGCGACAGTAACCATCGATAGCATACCGTA
basic 6HB	1[217]3[230]	TAAAATATTTCGAGCTGAAACATGAAAG
basic 6HB	1[1078]3[1091]	AAACTATAGTTGAATGTTTATCAACAAT
basic 6HB	0[1042]3[1035]	CTCGTTAGCCCTTCGGAAGCGCATTAGAGTCCAGA
basic 6HB	1[931]3[944]	AGAGGGGCCGAACGTCGCCATATTTAAC
basic 6HB	0[433]3[426]	TAGCATTTGGGCGCGTTTGCCTTTAGCGCACCCTC
basic 6HB	0[1000]3[993]	AGGGATTGGTGGTTAAGTCAGAGGGTAAAATAAGA
basic 6HB	0[727]3[720]	ACAGGTCAGGTCGAATAAAAGAAACGCAAAGAACG
basic 6HB	1[679]3[692]	AACTGGCAGTAACATATGTAAATGCTGA
basic 6HB	0[811]3[804]	AAAGATTCTCACAACCCAAAAGAACTGGTGATAAA
basic 6HB	0[307]3[300]	GAGAAGCTTCGCGTCCTCAGAGCCGCCAGCGGATA
basic 6HB	1[364]3[377]	GTCGAAATTTGCTATTTAGTACCGCCAC
basic 6HB	1[742]3[755]	CGGAACATGCACGTATTTTAGTTAATTT
basic 6HB	0[1084]3[1077]	GCGTACTCACGCTGATGAAAATAGCAGCACGCGCC
basic 6HB	0[958]3[951]	TAGCGTCGGCCAACACAAGAATTGAGTTAACGCCA
basic 6HB	0[937]3[930]	CGTCATATGCCAGCATAATAAGAGCAAGTTGAGAA
basic 6HB	1[805]3[818]	TAATGCACTGATTGTAAGGCGTTAAATA
basic 6HB	1[910]3[923]	TTTTGCAAGTTTGAACAGTAGGGCTTAA
basic 6HB	0[706]3[699]	CCTTTAACCAGTGCCACGGAATAAGTTTTGCAAAT
basic 6HB	0[160]3[153]	TTCTAGCCCGGTTGCCTCATTAAAGCCAGGTCAGT
basic 6HB	1[406]3[419]	GGCGCAGTCGTCTTACCCTCAGAGCCAC
basic 6HB	0[1021]3[1014]	GCTAAACTGAGACGATTAACTGAACACCGACAAAA
basic 6HB	1[658]3[671]	ATTACCTGGGAGAATTAGGTTGGGTTAT
basic 6HB	1[469]3[503]	TGTACAGACTACAAACACTGAGTTTCGTCAATAAATCAATATATGTGAG
basic 6HB	1[721]3[734]	TACGTTAGAAATTGCGAGAAAACTTTTT
basic 6HB	1[868]3[881]	CTCGTTTAGAAGGATATGCGTTATACAA
basic 6HB	0[517]3[510]	TTCGCAACCGGCACTAGCACCATTACCATGAATAA
basic 6HB	0[601]3[594]	TACGGTGGGGCCTCATTGACGGAAATTAACGCTGA
basic 6HB	0[685]3[678]	AAGAGGTCACGACGCACAATCAATAGAAATAACTA
basic 6HB	0[769]3[762]	TAATTCGTAATCATTGTTAGCAAACGTACATCTTC
basic 6HB	1[763]3[776]	AGAAAGAACCTACCTGACCTAAATTTAA
basic 6HB	1[847]3[860]	AAGAGCAATATTCCGAAAAAGCCTGTTT
basic 6HB	0[559]3[552]	AATTCTGCGCCATTCGACTTGAGCCATTTCCCTTA
basic 6HB	0[454]3[447]	CATCAATTCTGCCAAATCAGTAGCGACACAAGCCC
basic 6HB	0[202]3[195]	GGCCGGATAAGCAAGCAGGTCAGACGATCCCCCTG
basic 6HB	1[784]3[797]	TTTAGGACTGAATAAAATACCGACCGTG
basic 6HB	0[496]4[483]	GTTTAGCGATCGCAGGCCGGAAACGTCA

Continued on next page

Type	Name	Sequence
basic 6HB	0[895]3[888]	GTTCAGAGCGTTGCCCGAAGCCCTTTTTATTCTTA
basic 6HB	5[98]0[98]	AAACAAGAGAATCGTACAAAGGCTATCA
basic 6HB	1[238]3[251]	GAAGGCATGTATCGAGGCTGAGACTCCT
basic 6HB	2[1266]0[1253]	CCATTAAAAATACCAGATAGAAAATCGG
basic 6HB	2[1287]0[1274]	ACTGATAGCCCTAAAAAGCGTGTCGAGG
basic 6HB	2[153]0[140]	GCATAACCGATATATCGGAACTTAATGC
basic 6HB	4[146]1[153]	GAATGGACAATCATATGTACCTGATAAAGAGGGTA
basic 6HB	4[1238]1[1245]	TTAGTTGTCAAAGGGCGAAAAAAGGGAGATTCTGG
basic 6HB	4[1259]1[1266]	TTTTGAAATCAGGGCGATGGCAAGCACTACCCTTC
basic 6HB	4[1217]1[1224]	GCTACAATAAAGAACGTGGACCTTGACGACGACCA
basic 6HB	4[167]1[174]	AATAAATATAATCAGAAAAGCATGATATGGCTTTG
basic 6HB	2[1245]0[1232]	CCACCAGCAGAAGAAAGGGACCCCCCGA
basic 6HB	2[174]0[161]	ATGACAACAACCATCTACAGATCAACCG
basic 6HB	2[1224]0[1211]	GAGGTGAGGCGGTCCAGTCACGGGAAAG
basic 6HB	2[1203]0[1190]	ACACCGCCTGCAACTATTTACGAGAAAG
basic 6HB	4[1196]1[1203]	TTACCAATTTGGAACAAGAGTACGTGGCATTGGCA
basic 6HB	2[951]0[938]	ATTAAATCCTTTGCGTAATAGGCGGAAT
basic 6HB	4[1112]1[1119]	AGAAACGTCCTGTTTGATGGTCCACACCATTACCG
basic 6HB	2[552]0[539]	ATCAAGAAAACAAAACCCAAAGTAGATT
basic 6HB	4[944]1[951]	AAGCCCATGCATTAATGAATCCAATACTTAAAATG
basic 6HB	4[629]1[636]	TCAACCGGAAAGGGGGATGTGCTGTAGCGGTTTAA
basic 6HB	2[720]0[707]	CGTAGATTTTCAGGTGGGAAGGAGAGTA
basic 6HB	4[587]1[594]	TTCATTAAGGGCGATCGGTGCTCTGGAATGACGAG
basic 6HB	2[1140]0[1127]	TGCTGAACCTCAAATTGCAACGCTGCGC
basic 6HB	2[1056]0[1043]	AAAATATCTTTAGGTAGTAATTGCTTTC
basic 6HB	4[713]1[720]	AAGACACCAAGCTTTGCCTGCAGGATTAAAAAATC
basic 6HB	4[1049]1[1056]	AAAACAGACCGCCTGGCCCTGTATAACGAACATCA
basic 6HB	4[1091]1[1098]	GTCAAAAGTTTGCCCCAGCAGCGCCGCTGCTGGTA
basic 6HB	4[608]1[615]	GGTAAATTTCGCTATTACGCCATATGCAGTAGTAA
basic 6HB	2[636]0[623]	TGCTTTGAATACCATTGAGATTCAACAT
basic 6HB	2[1077]0[1064]	AGGAATTGAGGAAGGAGTAGACTTTGAC
basic 6HB	4[797]1[804]	CATGATTGAAATTGTTATCCGAAGAGGAATTCAAC
basic 6HB	2[762]0[749]	ATATCAAAATTATTACATTATAAGCGAA
basic 6HB	4[860]1[867]	GAACAAAGGGGTGCCTAATGAAAAAATCCATAACC
basic 6HB	4[671]1[678]	AATTCATAGGGTTTTCCCAGTCATTTTTTTTTAAG
basic 6HB	4[818]1[825]	CGGAATATTCCACACAACATAGCGGATTAACGCCA
basic 6HB	4[419]1[426]	TCAGACTCACGTTGGTGTAGAAACATCCATCATAA
basic 6HB	2[405]0[392]	TCCAGACGTTAGTACTTAGCCAAAGAAT
basic 6HB	4[440]1[447]	GAATCAAATCGTAACCGTGCATCTACTACCAACTT
basic 6HB	2[594]0[581]	TTCATTTCAATTACCTTGCCCGTTTCAT
basic 6HB	5[959]0[959]	GCGCGGGGAGAGGCGAATCCTGAGAAGA
basic 6HB	1[490]0[476]	CTGGCTGTGGAAACAGTACCCAGTACAAACCAGGCTCATTTG
basic 6HB	4[545]1[552]	TGGGAATGGCAAAGCGCCATTCGAACGATCAACGT
basic 6HB	4[1007]1[1014]	CTGAACATTTCTTTTCACCAGAGGAGGCACGCAAA
basic 6HB	2[888]0[875]	ACAAAGAAACCACCACCAGACAATGACC
basic 6HB	4[923]1[930]	AAACAATCGGGAAACCTGTCGAATATTCTTTTGCC
basic 6HB	4[398]1[405]	TCGGCATATTGACCGTAATGGGCAAGGCGGAACGA
basic 6HB	4[902]1[909]	TATCTTAGCTCACTGCCCGCTAAATGCTGAGAGGC
basic 6HB	2[804]0[791]	TTTGGATTATACTTATACCACAGCCCGA
basic 6HB	4[1175]1[1182]	TTTCCAGTAGGGTTGAGTGTTAGAAAGCCGTCTGA
basic 6HB	2[1161]0[1148]	GAAAAATCTAAAGCTGGAAATGCTGGCA
basic 6HB	2[930]0[917]	TTATTAATTTTAAAAAAGAAGATTGAAT
basic 6HB	2[573]0[560]	AAAGAAGATGATGACTGCTCAGATTCCC
basic 6HB	2[783]0[770]	ATGGAAGGGTTAGATTCATCACGCGTTT
basic 6HB	2[867]0[854]	GCGGAATTATCATCACACTATAGGTCTT
basic 6HB	2[426]0[413]	GATCTAAAGTTTTGACGGTCAAATAAAT
basic 6HB	2[468]0[455]	CGCCTGTAGCATTCGGACAGAAAGGTGG
basic 6HB	4[524]1[531]	TCACCAGCGCTTCTGGTGCCGACCATTAACCGGAT

Continued on next page

Type	Name	Sequence
basic 6HB	2[741]0[728]	AAAACAGAAATAAAATAAAACAACTCCA
basic 6HB	4[566]1[573]	CCGTCACCAGGCTGCGCAACTAACAGTTTTCAGTG
basic 6HB	4[734]1[741]	GCAACATCTCTAGAGGATCCCGGAAGCAGAACTAA
basic 6HB	2[531]0[518]	ACATTTAACAATTTGACAAGAGATACAT
basic 6HB	2[615]0[602]	AAATCGCGCAGAGGAGAACGAACTAAAG
basic 6HB	2[1182]0[1169]	CGCTGAGAGCCAGCGCTCAATGAAAGGA
basic 6HB	4[776]1[783]	CGCAGTAGGTCATAGCTGTTTCAAATATGTTGAGA
basic 6HB	4[461]1[468]	GCACCGTGTTTGAGGGGACGAAGCTGAATGAACGG
basic 6HB	2[846]0[833]	TGATTATCAGATGATTACGAGTAGTCAG
basic 6HB	2[972]4[959]	ACAAACAATTCGACAACTCGTACATGTAATTTAGGATAACCC
basic 6HB	2[1014]0[1001]	TCAATAGATAATACGTCCATCCGATTAA
basic 6HB	4[839]1[846]	ACCGAGGGAAGCATAAAGTGTACTATTAGCATAGT
basic 6HB	4[1133]1[1140]	TTATTTAAAATCGGCAAAATCCGGTCACAGGAAAA
basic 6HB	2[825]0[812]	TCATCAATATAATCGATACATGCATCAA
basic 6HB	4[1154]1[1161]	GTTACAAAATCAAAAGAATAGCTAGGGCACCTACA
basic 6HB	4[482]1[489]	CCAATGAATCGGCCTCAGGAATATATTTGCATAGG
basic 6HB	2[678]0[665]	GTACCTTTTACATCTATGCGAGCGGATG
basic 6HB	2[993]0[980]	GATTTAGAAGTATTGGCCACCGGAACGG
basic 6HB	2[384]0[371]	TTTCTGTATGGGATTCCGCGAAATAAAG
basic 6HB	2[363]0[350]	AACAACTTTCAACAGCCTGATGCTAAAT
basic 6HB	2[447]0[434]	AGCCCTCATAGTTAGAACTGAATAGTAG
basic 6HB	2[699]0[686]	TCAGATGAATATACTCATTATTTTTGAT
basic 6HB	4[881]1[888]	AAGAAAAAACTCACATTAATTAAACGAGGACGATA
basic 6HB	4[986]1[993]	TTGAGCGGTATTGGGCGCCAGTTAGACAGAGTAAA
basic 6HB	4[650]1[657]	CCAAAGAGCGATTAAGTTGGGGCTTAATATTGTGA
basic 6HB	4[356]1[363]	TTTTCATCGAGTAACAACCCGGCATAAAAAATTGT
basic 6HB	1[952]1[972]	TTTAGACTGGTGTTTTTATAA
basic 6HB	2[657]0[644]	ACAATAACGGATTCTTTAATCTGCTGAA
basic 6HB	2[909]0[896]	GTAACATTATCATTAAATAGCTTAAACA
basic 6HB	2[1119]0[1106]	CCCTCAATCAATATGAACAATCGCCGCG
basic 6HB	4[377]1[384]	CCCTTATCTCCGTGGGAACAAATTAAGCCCTGCTC
basic 6HB	4[1028]1[1035]	CGGGAGAGGCAACAGCTGATTGAATCAGAATACTT
basic 6HB	4[503]1[510]	TTAGCAACTCCAGCCAGCTTTATGGTCATCAAGAG
basic 6HB	2[510]0[497]	ATTACCTTTTTTAAACCTTCAATAACCT
basic 6HB	4[755]1[762]	GAAAATACGAGCTCGAATTCGAGCTTCATACAGGT
basic 6HB	4[1070]1[1077]	CTTTACATGCAGCAAGCGGTCATGGTTGAGAACTC
basic 6HB	2[1098]0[1085]	GTTGGCAAATCAACCGGCCTTACAGGGC
basic 6HB	4[692]1[699]	ATTTTGTTTGTAAAACGACGGTTGCTCCACCAGTC
basic 6HB	2[1035]0[1022]	ACAACTAATAGATTTTGTAGCAGCGGGA
basic 6HB	3[100]3[125]	CATGGCTTTTGATGATACAGGAGTGT
basic 6HB	0[139]3[132]	CGGAGAGAGCATGTAAGCGCAGTCTCTGACTGGTA
basic 6HB	0[118]4[112]	AGAGATCATGAACGCGTTCCA
basic 6HB	4[125]1[132]	AATTTACGTAATCGTAAAACTGGTAGCTAGCGAAA
basic 6HB	2[132]2[115]	GCTGAGGCTTGCAGGGAG
basic 6HB	1[115]0[119]	TCACCCTCAGCATTTTTG
marker binder 6HB	1[1183]3[1196]	taagtgagacccgtacatatAATGGATAGTGCCACAAGAACGGGTATTtaagtgagacccgtacatat
marker binder 6HB	0[1189]3[1182]	taagtgagacccgtacatatGAAGGGAGTTCCAGCGCTAACGAGCGTCATCATTCtaagtgagacccgtacatat
marker binder 6HB	0[1168]3[1161]	taagtgagacccgtacatatGCGGGCGCCCGAGAAGCCTAATTTGCCAAATAATCtaagtgagacccgtacatat
marker binder 6HB	0[1126]3[1119]	taagtgagacccgtacatatGTAACCAGGTTCCGTCCCAATCCAAATAAATAATAtaagtgagacccgtacatat
marker binder 6HB	1[1120]3[1133]	taagtgagacccgtacatatCCAGCCATATCAAATCCCATCCTAATTTtaagtgagacccgtacatat
marker binder 6HB	1[1162]3[1175]	taagtgagacccgtacatatTTTTGACAGCAAATGGCTGTCTTTCCTTtaagtgagacccgtacatat
marker binder 6HB	0[1210]3[1203]	taagtgagacccgtacatatCCGGCGACCACTATTTTTATCCTGAATCAAACCAAtaagtgagacccgtacatat
marker binder 6HB	1[1141]3[1154]	taagtgagacccgtacatatACGCTCAATCACCTTGTAGAAACCAATCtaagtgagacccgtacatat
marker binder 6HB	0[1147]3[1140]	taagtgagacccgtacatatAGTGTAGCCTTATAAATAAACAGCCATAACGAGCAtaagtgagacccgtacatat
marker binder 6HB	1[1267]3[1280]	taagtgagacccgtacatatTGACCTGAACATCGCCAATAGCAAGCAAtaagtgagacccgtacatat
marker binder 6HB	1[1204]3[1217]	taagtgagacccgtacatatGATTCACAGTATTAGTACCGCACTCATCtaagtgagacccgtacatat
marker binder 6HB	1[1246]3[1259]	taagtgagacccgtacatatCCAACAGGAACGAATCGTAGGAATCATTtaagtgagacccgtacatat
marker binder 6HB	0[1273]3[1266]	taagtgagacccgtacatatTGCCGTACCACTACCGACTTGCGGGAGGACCGCGCtaagtgagacccgtacatat

Continued on next page

Type	Name	Sequence
marker binder 6HB	1[1225]3[1238]	taagtgagacccgtacatatGTAATAATAAAACAAGCAAGCCGTTTTTtaagtgagacccgtacatat
marker binder 6HB	0[1231]3[1224]	taagtgagacccgtacatatTTTAGAGTCCAACGCTATTTTGCACCCAGAGAACAtaagtgagacccgtacatat
marker binder 6HB	0[1252]3[1245]	taagtgagacccgtacatatAACCCTAACCGTCTGCCTTAAATCAAGAATTTTCAtaagtgagacccgtacatat
marker binder 6HB	1[1288]3[1310]	taagtgagacccgtacatatCGTGGCATGCGCGAATAGAAGGCTTATCCGGTATTTTtaagtgagacccgtacatat
marker binder 6HB	0[1296]1[1287]	taagtgagacccgtacatatTTAAGTTTTTTGGGAAGAATAtaagtgagacccgtacatat
marker binder 6HB	4[1310]3[1287]	taagtgagacccgtacatatTTTTCTAAGAACGCGAGGCGTTTTAGCGATCAGATtaagtgagacccgtacatat
marker binder 6HB	4[1280]5[1296]	taagtgagacccgtacatatAACCTCCGTGAACCATCACCCAAATCTTtaagtgagacccgtacatat
marker binder 6HB	2[1315]1[1315]	taagtgagacccgtacatatTTTGGCTATTAGTCTTTAACAGACAATATTTTTGAATTtaagtgagacccgtacatat
marker dye	-	ATTO655-TT-ATATGTACGGGTCTCACTTA

MNC binding staples for the multi-MNC MADONA

Staple sequences used for binding MNCs over the full length of the structure used for Multi-MNC design.

TABLE II: Multi-MNC binder staples

Type	Name	Sequence
Multi-MNC binder	4[377]8[377]	CACAGACAGTTGCAGCAAGCGGCCTCAGCCTGACTAAAAAAAA
Multi-MNC binder	4[398]8[398]	ACTACAACTGGTTTGCCCCAGGAGGGGACAAAGCGAAAAAAAA
Multi-MNC binder	2[405]7[399]	GCTGACCTTCATCATCAGAAGCGACGACAAAAAAAA
Multi-MNC binder	4[419]8[419]	TGAGTTTAAATCCTGTTTGATTAACCGTGATTAAGAAAAAAAA
Multi-MNC binder	2[426]7[420]	ACAGACCAGGCGCATCAAAAAGCATCTGAAAAAAAA
Multi-MNC binder	4[440]8[440]	GGAACCCCCGAAATCGGCAAATTGGTGTACTTCAAAAAAAAAA
Multi-MNC binder	2[447]7[441]	AAGAGGACAGATGACCGAAAGAGATGGGAAAAAAAA
Multi-MNC binder	4[461]8[461]	TCAGGGAATAAATCAAAAGAAACCGTAATTCGAGCAAAAAAAA
Multi-MNC binder	2[468]7[462]	AACCGAACTGACCAGTTTTAATGGGATAAAAAAAAA
Multi-MNC binder	4[482]8[482]	TCAGAGCAGATAGGGTTGAGTGTGGGAAGACCGGAAAAAAAAA
Multi-MNC binder	1[490]7[483]	AGCAAACGTCTTTCCTTATCGGTCAATCCGAACCACAAACGGAAAAAAAA
Multi-MNC binder	4[503]8[503]	CCACCCTCAGTTTGGAACAAGTAACAACGGTCAGGAAAAAAAA
Multi-MNC binder	2[510]7[504]	ATCAATAATCGGCTTCCAACACCGTCGGAAAAAAAA
Multi-MNC binder	4[524]8[524]	GTACCGCTATTAAAGAACGTGTCAACATTTAATTGAAAAAAAA
Multi-MNC binder	2[531]7[525]	TTTACGAGCATGTAAGTACCTTAAATGTAAAAAAAA
Multi-MNC binder	4[545]8[545]	TCACCGTACGTCAAAGGGCGACCTTCCTAGGTCATAAAAAAAA
Multi-MNC binder	2[552]7[546]	AAAAATAATATCCCTGATAAGGTAGCCAAAAAAAAA
Multi-MNC binder	2[573]7[567]	AATAGATAAGTCCTGATGGCTTAATTCGAAAAAAAA
Multi-MNC binder	4[566]8[566]	GTATAGCTCTATCAGGGCGATTCAAAAATAGAGCTAAAAAAAA
Multi-MNC binder	2[594]7[588]	AGAACGCGCCTGTTTGAATATCAATAGGAAAAAAAA
Multi-MNC binder	4[587]8[587]	CCGTCGATACGTGAACCATCATTTTAACAATGCTGAAAAAAAA
Multi-MNC binder	4[608]8[608]	TCAGTACCAAGTTTTTTGGGGTTTTTGTTTAAATAAAAAAAAA
Multi-MNC binder	2[615]7[609]	AACAACATGTTCAGACATGTTTAAATCAAAAAAAAA
Multi-MNC binder	2[636]7[630]	TCTGTCCAGACGACAAAGTACAAATTCGAAAAAAAA
Multi-MNC binder	4[629]8[629]	TTAGGATGCCGTAAAGCACTATTTGTTAGGTGTCTAAAAAAAA
Multi-MNC binder	2[657]7[651]	ACCGACAAAAGGTATCATTCCGTAAACGAAAAAAAA
Multi-MNC binder	4[650]8[650]	TGAGACTACCCTAAAGGGAGCTTAAATTATATAACAAAAAAAA
Multi-MNC binder	2[678]7[672]	AGTAATAAGAGAATTCCCAATGTATAAGAAAAAAAA
Multi-MNC binder	4[671]8[671]	AACATGATTAGAGCTTGACGGGAAGATTTCTGCGAAAAAAAAA
Multi-MNC binder	4[692]8[692]	TTTCGGACGGCGAACGTGGCGTCAGAAATTTGACCAAAAAAAA
Multi-MNC binder	2[699]7[693]	AGGCAGAGGCATTTGATTTAGAGCCCCAAAAAAAAA
Multi-MNC binder	4[713]8[713]	ACAGTTAAAGGGAAAGCGAAACATATGTGCAAATGAAAAAAAA
Multi-MNC binder	2[720]7[714]	AACAACGCCAACATACATTTCACCCCGGAAAAAAAA
Multi-MNC binder	2[741]7[735]	TAATTGAGAATCGCACCTGTTACTAGCAAAAAAAAA
Multi-MNC binder	4[734]8[734]	TGAGTAAGCGCTAGGGCGCTGTCGTAAATAGCTATAAAAAAAA
Multi-MNC binder	4[755]8[755]	GTTTTAATAGCGGTCACGCTGAAGAGAACGCGAGCAAAAAAAA
Multi-MNC binder	2[762]7[756]	CCAACGCTCAACAGTTTGGGGTCGATGAAAAAAAAA
Multi-MNC binder	4[776]8[776]	ACAGGAGCCACCACACCCGCCGCCTGAGCAATTCTAAAAAAAA
Multi-MNC binder	2[783]7[777]	CAAATTCTTACCAGGTGGCATAGTCTGGAAAAAAAA

Continued on next page

Type	Name	Sequence
Multi-MNC binder	2[804]7[798]	TTTAGTATCATATGGTAGTAGGCTATCAAAAAAAAA
Multi-MNC binder	4[797]8[797]	TCATACAATGCGCCGCTACAGTACAAAGCATTAACAAAAAAAA
Multi-MNC binder	2[825]7[819]	ATAATTACTAGAAAAAATCATATTTTTGAAAAAAAA
Multi-MNC binder	4[818]8[818]	AATTTACACTATGGTTGCTTTGGTAGCTACAGGCAAAAAAAAA
Multi-MNC binder	4[839]8[839]	GAATGGAACGTATAACGTGCTTGATAAACAAAATTAAAAAAAA
Multi-MNC binder	2[846]7[840]	ATAAGAATAAACACGAATTAGTTAATGCAAAAAAAA
Multi-MNC binder	2[867]7[861]	GTGTGATAAATAAGAAAGCCTTCAACCGAAAAAAAA
Multi-MNC binder	4[860]8[860]	AATAAATTTAGAATCAGAGCGATGATATCAGAGCAAAAAAAAA
Multi-MNC binder	4[881]8[881]	TGGCCTTAACAGGAGGCCGATGACAGTCTTGTACCAAAAAAAA
Multi-MNC binder	2[888]7[882]	TAATGGTTTGAAATAAATCGGAAATCACAAAAAAAA
Multi-MNC binder	2[909]7[903]	TTTCATCTTCTGACTTATGACTGAGAAAAAAAAAAA
Multi-MNC binder	4[902]8[902]	GGTTGAGATTTTAGACAGGAAAAAAGGGCCTGTAAAAAAAAAA
Multi-MNC binder	4[923]8[923]	CCGCCGCCCAGAATCCTGAGAGTAATGTAAGCCTTAAAAAAAA
Multi-MNC binder	2[930]7[924]	TTTCAAATATATTTGCGGGAGGTAGGTAAAAAAAAA
Multi-MNC binder	4[944]8[944]	CCACCAGTTATAATCAGTGAGTATTTTAGATAAAAAAAAAAAA
Multi-MNC binder	2[951]7[945]	ACAAAGAACGCGAGACGCAAGAATGCAAAAAAAAAA
Multi-MNC binder	5[959]7[966]	AGTAAAAGAGTCTGTTAGACTTTACAACAAAAAAAA
Multi-MNC binder	1[952]8[965]	ATTTTTAGAAACAATTCGACAAAAAAAAA
Multi-MNC binder	2[993]7[987]	TATATAACTATATGTTAAATCAGGATTTAAAAAAAA
Multi-MNC binder	4[986]8[986]	CCCTCAGCGCAAATTAACCGTACATTTGCTTTGCCAAAAAAAA
Multi-MNC binder	4[1007]8[1007]	CCGCCTCATACTTCTTTGATTTTAGAGCTTTAAAAAAAAAAAA
Multi-MNC binder	2[1014]7[1008]	AACCTCCGGCTTAGTATTAATCGTCAATAAAAAAAA
Multi-MNC binder	2[1035]7[1029]	GGTCTGAGAGACTATAACATTTAACAACAAAAAAAA

MNC binding staples of the 4-MNC MADONA

Staple sequences used for binding of MNCS on the 4-MNC structure.

TABLE III: 4 MNC-binding staples

Type	Name	Sequence
4-MNC binder	8[392]3[398]	AAAAAAAAATTATAGAGAGTAAAGCGCGAAACAAAG
4-MNC binder	7[384]3[384]	AAAAAAAAAGTATCGGTCCACGCGCCTGTAGCATTCTATACCA
4-MNC binder	7[405]3[405]	AAAAAAAACCAGTTTCAGGCGACGTCACCAGTACAATACAACG
4-MNC binder	8[413]3[419]	AAAAAAAAGATTGCATAGGCTGGAGATTTGTATCAT
4-MNC binder	8[434]3[440]	AAAAAAAAAGGAAGCACGGTGTTAAATTGTGTCGAA
4-MNC binder	7[426]3[426]	AAAAAAAACGCATCGGGTGGTTATGTACCGTAACACCGCCTGA
4-MNC binder	8[581]3[587]	AAAAAAAATAATTGCTATCAACCCAATAGCAAGCAA
4-MNC binder	7[573]3[573]	AAAAAAAAAACGCCAGGCCCACGAGGGTTGATATAAACCGCGC
4-MNC binder	7[594]3[594]	AAAAAAAAGCTCATTCCCAAATCAGGCGGATAAGTGATCAGAT
4-MNC binder	8[602]3[608]	AAAAAAAATAGCTCACTAATGCATAGAAGGCTTATC
4-MNC binder	8[623]3[629]	AAAAAAAATGCAACTGACAATACTAAGAACGCGAGG
4-MNC binder	7[615]3[615]	AAAAAAAACATTAAATCGAGGTTAGCGGGGTTTTGCCGGTATT
4-MNC binder	8[791]3[797]	AAAAAAAAACTAATACGTTATAATATTATTTATCCC
4-MNC binder	7[783]3[783]	AAAAAAAAGGTCATTGCGCTTATGGCTTTTGATGATAACAGCC
4-MNC binder	7[804]3[804]	AAAAAAAAAGAGATCGGCGCGTCGTTCCAGTAAGCGAATCCAA
4-MNC binder	8[812]3[818]	AAAAAAAAATCCAATAAGCCTGATAAGAAACGATTT
4-MNC binder	8[833]3[839]	AAAAAAAAAGGCAAACGGAATCAACGTCAAAAATGA
4-MNC binder	7[825]3[825]	AAAAAAAACGGAGAGGACGAGCAAGCGCAGTCTCTGTTTGTTT
4-MNC binder	8[980]3[986]	AAAAAAAAACTCGTATAAATGCGTTAAGCCCAATAA
4-MNC binder	7[972]3[972]	AAAAAAAAAGAAGTATCCATCAAACCGCCACCCTCAGAATTGA
4-MNC binder	7[993]3[993]	AAAAAAAAAGATAATTGTAGCACCTCAGAGCCGCCATAAGAGC
4-MNC binder	8[1001]3[1007]	AAAAAAAACGAACGTGTTGGGTAAGAAACAATGAAA
4-MNC binder	8[1022]3[1028]	AAAAAAAAGTTTGAGCCTTTTTAGCTATCTTACCGA
4-MNC binder	7[1014]3[1014]	AAAAAAAATAATAGAAGTAATAAGCCACCACCGGAATAGCAAT

Modifications staples

Sequences of modification staples for either blunt end or passivated 6HB variant as well as for the addition of the Biotin mid- and end pivot.

TABLE IV: Modification staples

Type	Name	Sequence
Blunt Endpivot		
Biotin-End Multi-MNC	3[98]3[125]	Biotin-TTCAATGACAACAACCATCGCCCACGCA
Endpivot Multi-MNC	5[86]0[86]	AGCTGTTTCCTGTGTGAAATTGTTATGAGCTCGAATTCGTAATCATGGTCAT
Endpivot Multi-MNC	0[118]4[100]	GGGTACCCCGCTCAATACCGATAGTTGCGCCGA
Endpivot Multi-MNC	2[132]2[103]	AAGATTCATCAGTTGAGATTTAGGAATACC
Endpivot Multi-MNC	1[103]0[119]	ACATTCAACTAATGCAGATACATGATCCCC
Midpivot		
Biotin-Mid 4-MNC	6[705]5[713]	Biotin-TTCTACAATTTTACAGTTAAAGGGAA
Midpivot 4-MNC	2[692]0[686]	GGCATTTGATTTAGAGCCCCA
Midpivot 4-MNC	0[706]3[703]	TTGATAAAGAAAGGATGCCCCCTGCCTATTTGCACCCAG
Midpivot 4-MNC	1[700]2[693]	ATTAGATGTAATTTAGGCAGA
Midpivot 4-MNC	5[714]1[720]	AGCGAAACATATGTGCAAATG
End-passivation		
End-passivation	5[80]0[80]	TTTTTTAGCTGTTTCCTGTGTGAAATTGTTATGAGCTCGAATTCGTAATCATGGTCATTTTTTT
End-passivation	0[118]4[94]	GGGTACCCCGCTCAATACCGATAGTTGCGCCGATTTTTT
End-passivation	1[97]0[119]	TTTTTTACATTCAACTAATGCAGATACATGATCCCC
End-passivation	2[132]2[97]	AAGATTCATCAGTTGAGATTTAGGAATACCTTTTTT
End-passivation	3[94]3[125]	TTTTTTCAATGACAACAACCATCGCCCACGCA

[1] Chowdhury, M. S. *et al.* Decoupling the characteristics of magnetic nanoparticles for ultrahigh sensitivity. *Nano Letters* **23**, 58–65 (2022).

[2] Lak, A. *et al.* Cooperative dynamics of dna-grafted magnetic nanoparticles optimize magnetic biosensing and coupling to dna origami. *Nanoscale* **16**, 7678–7689 (2024).

[3] Jin, Z. *et al.* Modification of poly(maleic anhydride)-based polymers with H_2N-R nucleophiles: Addition or substitution reaction. *Bioconjugate Chemistry* **30**, 871–880 (2019).

[4] Pellegrino, T. *et al.* Hydrophobic nanocrystals coated with an amphiphilic polymer shell: A general route to water soluble nanocrystals. *Nano Letters* **4**, 703–707 (2004).

[5] Yu, W. W. *et al.* Forming biocompatible and nonaggregated nanocrystals in water using amphiphilic polymers. *Journal of the American Chemical Society* **129**, 2871–2879 (2007).

[6] Douglas, S. M. *et al.* Rapid prototyping of 3d dna-origami shapes with cadnano. *Nucleic Acids Research* **37**, 5001–5006 (2009).

[7] Stahl, E., Martin, T. G., Praetorius, F. & Dietz, H. Facile and scalable preparation of pure and dense dna origami solutions. *Angewandte Chemie International Edition* **53**, 12735–12740 (2014).

[8] Rothfischer, F., Vogt, M., Kopperger, E., Gerland, U. & Simmel, F. C. From brownian to deterministic motor movement in a dna-based molecular rotor. *Nano Letters* **24**, 5224–5230 (2024).

[9] Büchl, A. *et al.* Energy landscapes of rotary dna origami devices determined by fluorescence particle tracking. *Biophysical Journal* (2022).

[10] Kopperger, E. *et al.* A self-assembled nanoscale robotic arm controlled by electric fields. *Science* **359**, 296–301 (2018).

[11] Schnitzbauer, J., Strauss, M. T., Schlichthaerle, T., Schueder, F. & Jungmann, R. Super-resolution microscopy with dna-paint. *Nature protocols* **12**, 1198–1228 (2017).

[12] Vogt, M. roboarmanalysis. `https://github.com/matvogt/roboarmanalysis` (2026). GitHub repository, accessed 2026-05-21.

[13] Garcıa-Otero, J., Garcıa-Bastida, A. & Rivas, J. Influence of temperature on the coercive field of non-interacting fine magnetic particles. *Journal of Magnetism and Magnetic Materials* **189**, 377–383 (1998).
